# Target enrichment sequencing coupled with GWAS identifies *MdPRX10* as a candidate gene in the control of budbreak in apple

**DOI:** 10.3389/fpls.2024.1352757

**Published:** 2024-02-21

**Authors:** Amy E. Watson, Baptiste Guitton, Alexandre Soriano, Ronan Rivallan, Hélène Vignes, Isabelle Farrera, Bruno Huettel, Catalina Arnaiz, Vítor da Silveira Falavigna, Aude Coupel-Ledru, Vincent Segura, Gautier Sarah, Jean-François Dufayard, Stéphanie Sidibe-Bocs, Evelyne Costes, Fernando Andrés

**Affiliations:** ^1^ UMR AGAP Institut, Univ Montpellier, CIRAD, INRAE, Institut Agro, Montpellier, France; ^2^ CIRAD, UMR AGAP Institut, Montpellier, France; ^3^ French Institute of Bioinformatics (IFB) - South Green Bioinformatics Platform, Bioversity, CIRAD, INRAE, IRD, Montpellier, France; ^4^ Genome Centre, Max Planck Institute for Plant Breeding Research, Cologne, Germany; ^5^ Centro de Biotecnología y Genómica de Plantas, Instituto de Investigación y Tecnología Agraria y Alimentaria, Universidad Politécnica de Madrid, Madrid, Spain

**Keywords:** capture, dormancy, budbreak, flowering, peroxidase, redox, CBF, cold-perception

## Abstract

The timing of floral budbreak in apple has a significant effect on fruit production and quality. Budbreak occurs as a result of a complex molecular mechanism that relies on accurate integration of external environmental cues, principally temperature. In the pursuit of understanding this mechanism, especially with respect to aiding adaptation to climate change, a QTL at the top of linkage group (LG) 9 has been identified by many studies on budbreak, but the genes underlying it remain elusive. Here, together with a dessert apple core collection of 239 cultivars, we used a targeted capture sequencing approach to increase SNP resolution in apple orthologues of known or suspected *A. thaliana* flowering time-related genes, as well as approximately 200 genes within the LG9 QTL interval. This increased the 275 223 SNP Axiom^®^ Apple 480 K array dataset by an additional 40 857 markers. Robust GWAS analyses identified *MdPRX10*, a peroxidase superfamily gene, as a strong candidate that demonstrated a dormancy-related expression pattern and down-regulation in response to chilling. *In-silico* analyses also predicted the residue change resulting from the SNP allele associated with late budbreak could alter protein conformation and likely function. Late budbreak cultivars homozygous for this SNP allele also showed significantly up-regulated expression of *C-REPEAT BINDING FACTOR* (*CBF*) genes, which are involved in cold tolerance and perception, compared to reference cultivars, such as Gala. Taken together, these results indicate a role for *MdPRX10* in budbreak, potentially via redox-mediated signaling and *CBF* gene regulation. Moving forward, this provides a focus for developing our understanding of the effects of temperature on flowering time and how redox processes may influence integration of external cues in dormancy pathways.

## 1 Introduction

Flowering in apple (*Malus domestica* Borkh.) occurs at the conclusion of floral bud dormancy, a protective mechanism that enables survival during the cold conditions of winter and resumption of growth when temperatures become more favorable. As the entrance and progression of floral bud dormancy are closely linked to seasonal temperatures, higher winter averages and unpredictable weather events present a significant challenge to maintaining fruit yield and quality.

In apple and many temperate fruit tree species, the winter dormancy period of floral buds proceeds in three parts: paradormancy, endodormancy, and finally ecodormancy, as described by Lang et al. (1987). Firstly, paradormancy inhibits budbreak of newly formed buds through internal factors, such as hormonal regulation, originating from other tissues. Cooler temperatures then induce entry into endodormancy, where, due to maintenance of this state by internal cues, buds are unable to develop further until a cultivar-specific chilling requirement has been met (Hauagge and Cummins, 1991). Following accumulation of this chilling, the bud begins ecodormancy and becomes competent to outgrow. It now relies on sufficient heating before budbreak and flowering can occur. High temperatures in spring can lead to rapid fulfilment of this heating requirement, triggering early flowering and exposure of new growth to damaging frosts (Cannell and Smith, 1986; Legave et al., 2008). As winter temperatures elevate, eventual inadequate chilling can delay the end of endodormancy, leading to late and desynchronized flowering, thus lowering yield and fruit quality (Petri and Leite, 2004; Atkinson et al., 2013). Already in many regions worldwide, chemical intervention with agents such as hydrogen cyanamide is required to overcome insufficient chilling and trigger budbreak (Carvajal-Millán et al., 2007).

Progression in our understanding of the genes involved in the dormancy cycle and budbreak timing could highlight avenues to improve climate resilience through targeted cultivar selection and advanced breeding techniques. Already, genetic dissection of bud dormancy has uncovered several key players, including *DORMANCY-ASSOCIATED MADS-BOX* (*DAM*) genes, a group of transcription factors first identified in the *evergrowing* peach (*Prunus persica* (L.) Batsch) mutant, where terminal vegetative growth was observed to continue even in the presence of normally dormancy-inducing conditions (Rodriguez et al., 1994; Bielenberg et al., 2008). In many rosaceous fruit tree species, the expression pattern of several *DAM* genes has been related to a role in establishment and continuance of endodormancy, with peak expression occurring during this time. A gradual reduction in expression follows, which reaches minimal levels corresponding to endodormancy release (Falavigna et al., 2019). *DAM* genes share high sequence homology with the *A. thaliana SHORT VEGETATIVE PHASE* (*SVP*) genes (Jiménez et al., 2009) although they are distinct from other *SVP*-like genes in apple, which also play a relevant role in dormancy progression (Wu et al., 2017). Missexpression of *DAM* and *SVP*-like genes in transgenic apple trees indicates they are both integral to dormancy and budbreak regulation (Wu et al., 2017; Moser et al., 2020; Wu et al., 2021). Furthermore, *SVP* and *DAM* genes are known to participate in gene networks that integrate distinct internal and environmental cues to regulate dormancy and budbreak timing (Falavigna et al., 2021; Lempe et al., 2022). In apple, these gene regulatory networks converge in the transcriptional regulation of *BRANCHED 1* (*MdBRC1*), which in homologous genes of many plant species encodes a potent bud growth repressor (Wang et al., 2019; Falavigna et al., 2021). Despite the importance of apple *SVP*, *DAM* and *BRC1* genes, a clear association between their genetic variability and the timing of floral or vegetative budbreak has yet to be demonstrated.

The utilization of a multifamily and pedigree-based studies has uncovered QTLs that co-localize with *DAM* genes on linkage groups (LG) 8 and 15, as reported by Allard et al. (2016). However, it is noteworthy that the most frequently observed QTL in apple is situated on LG9, as documented by van Dyk et al. (2010), Celton et al. (2011), and Urrestarazu et al. (2017). A region at the top of LG9 has been closely linked to budbreak dates in apple across multiple association studies, using both vegetative and floral budbreak phenotypes, which are thought to follow similar dormancy dynamics (Malagi et al., 2015). This QTL was first identified by Conner et al. (1998), when vegetative budbreak was linked to a region on LG3, which was later shown to be homologous to LG9 by Kenis and Keulemans (2004). Since then, the same region has been associated to budbreak in many bi-parental populations across different environments (van Dyk et al., 2010; Celton et al., 2011; Allard et al., 2016; Trainin et al., 2016; Miotto et al., 2019; Cornelissen et al., 2020) and in a large European association panel (Urrestarazu et al., 2017). A QTL in a homologous position has also been detected in pear (*Pyrus communis* L.) for vegetative budbreak (Gabay et al., 2017) and flowering time (Ntladi et al., 2018).

Yet, despite this ubiquitous detection, the gene(s) underlying this QTL remain elusive. A number of viable candidates have been proposed, aided by the presence of numerous known dormancy- and flowering-related genes in the interval. Amongst these are a *FLOWERING LOCUS C-like* (*MdFLC-like*, MD09G1009100) gene, which shows higher expression towards endodormancy release and into ecodormancy in vegetative and floral buds of apple and may act is a potential repressor of budbreak during this time (Porto et al., 2015; Miotto et al., 2019; Falavigna et al., 2021; Nishiyama et al., 2021). In *A. thaliana*, FLC inhibits flowering by repressing the expression of *FLOWERING LOCUS T* (*FT*), which encodes a phosphatidylethanolamine-binding protein (PEBP) that acts as a universal regulator of flowering in plants (Kardailsky et al., 1999; Kobayashi et al., 1999). The transcriptional upregulation of the homologous apple *FT* gene, *MdFT2*, after dormancy has been related to budbreak activation in apple (Lempe et al., 2022). Another previously proposed candidate is *PACLOBUTRAZOL RESISTANCE 1* (*MdPRE1*; MD09G1049300), a gene involved in gibberellin (GA) response and flower development in *A. thaliana* (Zhang et al., 2009). Differential expression of *MdPRE1* during dormancy in cultivars with contrasting chilling requirements suggests an equally important role in apple flowering (Porto et al., 2015). *MdPRE1* may affect the levels of GA (Lee et al., 2006), which in turn acts oppositely of abscisic acid (ABA) within a hormonal balance that halts bud growth at dormancy onset through increased levels of ABA and lower levels of GA, and promotes its subsequent renewal in spring, with rising GA and reduced ABA (reviewed by Liu and Sherif, 2019). Another candidate is *INDUCER OF C-REPEAT BINDING FACTOR* (*CBF*) *EXPRESSION 1* (*MdICE1*; MD09G1003800), a transcription factor that influences expression of *CBF* genes, which regulate cold response in apple via cold-regulated (*COR*) genes (Feng et al., 2012). Increased cold tolerance and early entry into dormancy were observed following overexpression of peach *CBF1* in apple (Wisniewski et al., 2011). This also resulted in delayed budbreak, likely via CBF regulation of *DAM* genes (Mimida et al., 2015; Wisniewski et al., 2015). These observations imply direct involvement by the CBF transcription factors in regulating the expression levels of *DAM* genes under cold conditions. Experimental validation of CBF transcription factor binding to the promoter regions of *DAM* genes has been documented in various temperate tree species (Falavigna et al., 2019). For instance, CBF transcription factors from Japanese pear (*Pyrus pyrifolia)* were shown to induce the expression of *PpyDAM1-1* and *PpyMADS13-3* genes in transient reporter assays (Saito et al., 2015; Niu et al., 2016). As chilling accumulation is key to endodormancy release, the signals by which temperature is able to trigger the *COR* response are of great interest. Reactive oxygen species (ROS) and bud redox state may act as this link. A lack of chilling in apple can be partially overcome through exogenous hydrogen peroxide (H_2_O_2_) application (Kuroda et al., 2005), which acts as a redox signaling molecule, and increased levels of H_2_O_2_ have been observed in the bud at the end of endodormancy and budbreak (Pérez et al., 2008; Sapkota et al., 2021).

Even with these potential candidate genes, and others, in this region, identifying which contribute to the detection of the LG9 QTL is difficult. The use of different populations and phenotypes across association studies could easily explain many of the differences in interval size and allele effect although it is also likely that multiple causative polymorphisms underlie this QTL. The direction of QTL effect can vary between populations, suggesting multiple genes or potentially multiple alleles within the same gene are responsible for the signal. For example, Trainin et al. (2016) found a haplotype within the QTL interval associated with early vegetative budbreak in the low chill cultivar, Anna, which was also only found in the early budbreak cultivars in their germplasm collection. Conversely, the QTL has also been found in populations from crosses between parents of moderate to high chilling requirement and in these cases does not appeared linked to early budbreak (Celton et al., 2011). In any event, within all these studies, the attribution of candidate gene has stemmed more from their location and potential roles in budbreak rather than any examination of their coding sequence or consequence of any identified polymorphisms.

Target capture is a method of enriching sequencing coverage in preselected regions of the genome (Albert et al., 2007; Gnirke et al., 2009). This enables higher resolution detection of polymorphisms present in genomic regions of interest and can provide greater power to determine the causative mutations underlying QTLs. In this study, we employed targeted capture sequencing to enhance marker coverage within the QTL interval on LG9, as well as in known dormancy- and flowering-related genes, using a large, diverse apple core collection and nine years of phenological data, to identify the key genetic driver(s) underlying this QTL. We identified a strong association between a SNP in the apple *PEROXIDASE 10* (*MdPRX10*) gene coding sequence and late budbreak, which has relevant effects on the MdPRX10 protein structure and functionality. We propose this gene provides a potential link between redox and cold signaling in the control of dormancy and floral budbreak timing in apple trees. These findings will advance our understanding of the genetics of budbreak in apple and potentially provide genetic tools to improve adaptation of dormancy to warmer climates.

## 2 Materials and methods

### 2.1 Plant material and experimental design

Plant material used consisted of 239 cultivars of a dessert apple (*Malus domestica* Borkh.) core collection, derived from a larger French collection of 2163 accessions and representing approximately 90% of total dessert apple allelic diversity (Lassois et al., 2016; list of cultivars given in **Supplementary Table S1**). All trees were grafted onto the M9 Pajam^®^2 rootstock, and planted in field conditions at the INRAE Diascope experimental unit (43°360N, 03°580E, near Montpellier, France). Planting occurred in 2014 for all but eleven trees, which required replacement in 2015. Four trees per cultivar (956 trees in total) were organized in 10 rows of 100 trees. Two cultivar replicates, placed opposite to each other in adjacent rows, were distributed randomly within the field.

### 2.2 Phenotyping

The timing of budbreak, specifically, stage C3 as defined by Baggiolini (1980), was recorded from 2015 (when most trees were one year old) and continued yearly until 2023. This stage was recorded as the number of days from January 1^st^ until when approximately 10% of floral buds had opened sufficiently for at least 10 mm of the leaf tips to have emerged past the bud scales. As our aim was to explore a QTL that has been detected across various phenotypes related to budbreak, and not an exploration of QTLs driven by specific environmental conditions, calendar days to budbreak was a simple measure that encompassed the many biological processes that contribute to dormancy and final budbreak.

However, to characterize chilling and heating conditions in the orchard, the accumulated Chilling Hours (CH) and accumulated Growing Degree Hours (GDH) were calculated from September till June for every year (2014/2015 – 2022/2023; **Supplementary Figure S1**). The number of CH accumulated up to the point of endodormancy release, identified by the Tabuenca test (described in section 2.6) performed on Gala, here used as a reference cultivar, was calculated using the ‘Chilling_Hours’ function of the chillR R package (Luedeling et al., 2023) and the data collected by the on-site weather station. This function is based on the method described by Bennett (1949), where each hour with a temperature between 0 and 7.2°C is considered one CH. GDH was calculated with the same R package using the ‘GDH’ function, which is based on the GDH model suggested by Anderson et al. (1986).

### 2.3 Calculation of genotypic values

To calculate the genotypic component of budbreak timing across all years for characterization of the trait and for use in the subsequent GWAS analyses, several linear mixed models were tested using R statistical software (R Core Team, 2023) and the lme4 R package (Bates et al., 2015). The full model included genotype (G) and genotype-year interaction (G:Y) as random effects and year (Y), row and planting date as fixed effects. Selection of fixed effects was applied based on the lowest Bayesian Information Criterion (BIC), to yield a final model (the full model), which was used to estimate variances and extract the random effects (Best Linear Unbiased Predictions, BLUPs) of G. The significance of the random effects was determined with the lmerTest R package (Kuznetsova et al., 2017) and ANOVA with the lmer R package for the fixed effect of Y. The broad-sense heritability ( $H^{2}$ ) of budbreak, adjusted for the number of replicates and years, was calculated from the variance components of the selected model as follows:


$$H^{2}=VarG/\left(VarG+VarG:Y/nyears+VarR/nrep\right)$$

where *VarG* is the genotypic variance, *VarG:Y* is the genotype-year interaction, *nyears* is the number of years of phenotypic records, *VarR* is the residual variance and *nrep* is the number of replicates per genotype. Bootstrapping was performed to calculate a 95% confidence interval for $H^{2}$ using the bootMer function in the lme4 R package with 1000 permutations.

Genotypic BLUPs were also calculated for each individual year to characterize the spread of budbreak of cultivars over time. The full model for each year included G, as a random effect, and row and planting date as fixed effects. A model per year was selected based on the lowest BIC and these are given in **Supplementary Table S2**. The BLUPs of G were extracted from each model.

### 2.4 Selection of genes, bait design, library preparation, and capture sequencing

Targeted capture sequencing is a method by which specific genes can be selectively amplified and sequenced, which, in this study, was used to enrich a SNP marker dataset with the sequence polymorphisms located in our genes of interest (reviewed in Andermann et al., 2020). The apple genes targeted by capture sequencing were defined from a list of gene families related to (1) flowering time control and previously described in *A. thaliana* (Bouché et al., 2016), and (2) apple genes found within the LG9 QTL, previously associated to budbreak (Trainin et al., 2016). The gene family evaluation consisted of phylogenomic and synteny analyses of 29 genomes, including apple (GDDH13 v1.1; Daccord et al., 2017), pear (RefSeq annotation release 101, assembly GCF_000413155.1_DPV01), peach (GCF_000442705.1_EG5), *Brassica napus* (GCF_001433935.1_IRGSP-1.0), olive tree (*Olea europaea*; vOE6; Cruz et al., 2016) and Arabidopsis TAIR10. These analyses were performed using GenFam tools (https://genfam.southgreen.fr), which relies on Galaxy workflows (https://github.com/SouthGreenPlatform/galaxy-wrappers) and the program IDEVEN (https://github.com/Delphine-L/IDEVEN), which parses synteny blocks predicted by CoGe Synmap (Lyons and Freeling, 2008) and RapGreen tools (Dufayard et al., 2021).

Probes targeting the apple genes were commercially synthetized by MYcroarray in a custom MYbaits kit (Daicel Arbor Biosciences, MI, USA). Total genomic DNA was extracted from dried leaves of all genotypes using an automated method, adapted from Risterucci et al. (2009), on a Biomek FXP (Beckman Coulter, CA, USA) and using the NucleoMag Plant Kit (Macherey–Nagel, Düren, Germany). DNA was quantified and normalized to 15 ng/µL with a Fluoroskan Ascent FL fluorometer (Thermo Fisher Scientific, MA, USA). The NGS sample library construction was performed with 1 µg of DNA per accession, using a preparation protocol developed at the GPTRG Facility at CIRAD (UMR-AGAP Institute, Montpellier, France) and adapted from Meyer and Kircher (2010) and Kircher et al. (2012). DNA samples were sheared to an average length of 300 bp on a Bioruptor^®^ Standard instrument (Diagenode, Liège, Belgium; Cat No. UCD-200). Sequence capture by hybridization was performed according to the manufacturer’s protocol (v.2) for the MYbaits target capture kit with the custom oligonucleotide library designed by Daicel Arbor Biosciences. Sequencing-by-synthesis was performed at the Max Planck Genome Centre (Cologne, Germany) on a HiSeq2500 with a HiSeq Rapid SBS Kit v2 from Illumina (San Diego, U.S.A.) in 2 x 250 bp paired-end read mode.

Resulting sequence data were treated with Cutadapt (v. 3.5; Martin, 2011) to remove remaining adapters and bases with a quality score of less than 30, at both the 5′ and 3′ read ends. Trimmed reads of less than 35 bp were discarded. The BWA-MEM software package (v.0.7.15; Li and Durbin, 2010) then mapped the reads onto the GDDH13 apple genome v1.1 (Daccord et al., 2017). Picard tools (v.2.24; https://github.com/broadinstitute/picard) were used to detect and remove PCR and optical duplicates. The Genome Analysis Toolkit (GATK; v.4.1.6; McKenna et al., 2010) function HaplotypeCaller was applied to perform variant calling. The resulting VCF file was filtered using vcftools to retain alleles located at the position of targeted flowering genes with a depth > 8, a minor allele count of 1 and a maximum of 90% missing data (corresponding to –minDP 8 –max-missing 0.9 –mac 1 parameters). Resulting variants were annotated using SNPeff (Cingolani et al., 2012; http://pcingola.github.io/SnpEff/#snpeff). The analysis workflow can be found at https://github.com/Alexandre-So/Workflow-snakemake-capture and https://github.com/SouthGreenPlatform/Workflow-snakemake-capture. The data have been deposited in the NCBI BioProject database (https://www.ncbi.nlm.nih.gov/bioproject), with BioProject accession number PRJNA1023873.

### 2.5 Genome-wide association studies (GWAS)

GWAS analyses were employed to identify SNP markers significantly associated to the budbreak timing phenotype with the aim of identifying genes involved in the control of this trait (reviewed Korte and Farlow, 2013; Alseekh et al., 2021). The SNP dataset derived from capture sequencing (116 296 SNPs) was merged with that of an Axiom^®^ Apple 480 K array, which consisted of 275 223 SNPs (Bianco et al., 2016; Denancé et al., 2022). From this, only bi-allelic SNPs and those that had been successfully mapped to the genome were kept. Markers with a minor allele frequency (MAF) of ≤0.05 or >95% heterozygosity were removed to yield a final dataset of 290 150 SNPs (40 857 from capture sequencing and 249 293 from the array).

Two mixed model GWAS approaches were employed to identify any significant associations between SNP markers and the timing of budbreak. Firstly, a single-locus method, performed by GEMMA software (v.0.97; Zhou and Stephens, 2012), which fitted the following model on a SNP-by-SNP basis:


Y=μ+Xβ+g+e,

where Y is the vector of the G BLUPs, μ is the overall mean, X is the vector of SNP dosage scores (0, 1 or 2 to denote the number of copies of the alternative allele), β is a vector of additive effect sizes, g is a vector of random polygenic effects and e is a vector of random residual effects. The distributions of g and e were assumed as $g\sim N\left(0,G\sigma_{g}^{2}\right)$ and $e\sim N\left(0,I\sigma_{e}^{2}\right)$ , where G is the realised genomic relationship (kinship) matrix, calculated using Method 1 described in VanRaden (2008), using the rutilstimflutre R package (Flutre, 2019), I is an identity matrix, $\sigma_{g}^{2}$ is the genetic variance and $\sigma_{e}^{2}$ is the residual variance. A SNP effect was considered significant when the Wald test *p*-value was smaller than the Bonferroni threshold to control for a family-wise error rate of 5%.

The second GWAS method was a multi-locus approach using the MLMM R package (Segura et al., 2012). This uses a step-wise regression process to identify SNPs of large effect while controlling for population structure and handling linkage disequilibrium. The procedure begins with a SNP-by-SNP model, after which each subsequent iteration includes the SNP with the lowest *p*-value from the previous iteration as a fixed effect. This continues for a fixed number of iterations (set here at seven) or until almost all variance is explained by the identified SNPs, rather than the polygenic effect. The model selected was that with the largest number of SNPs with an F test *p*-value smaller than the Bonferroni threshold (mBonf criterion; Segura et al., 2012).

The Bonferroni threshold, used for both GWAS analyses, was calculated based on the effective number of independent SNPs (M_eff_; Cheverud, 2001), estimated with the simpleM method (Gao et al., 2008, 2010), which uses a principal component approach to filter correlated SNPs. This resulted in an estimate of 85 159 independent SNPs and thus a significance threshold of -log_10_(*p*-value) = 6.23.

The narrow-sense heritability ( $h^{2}$ ) of budbreak timing was extracted from the MLMM output and was calculated as the ratio of the variance explained by G to the total BLUP variance in the first model iteration, where no SNPs were yet included as cofactors.

To test the significance of the differences in budbreak date between genotypes of the various allelic combinations of the SChr09_680633 SNP identified in the GWAS analyses, the emmeans R package (Lenth, 2022) was used to perform an ANOVA, followed by the Tukey method to account for multiple testing. The BLUPs generated from all the years of data together were used for this analysis.

### 2.6 Determination of end of endodormancy

The end of endodormancy was determined for four cultivars located at the Diascope experimental unit in 2023, following the 2022/2023 winter, using the Tabuenca test, a method of defining the date of endodormancy release based on the change in weight of floral primordia during this time (Tabuenca, 1964; Malagi et al., 2015). Selected cultivars included three homozygous for the alternative T allele of the SChr09_680633 SNP identified in the GWAS analyses: X9267, X8390 and X8717, and one, Gala, homozygous for the reference A allele of the same marker. Gala was not a member of the core collection but as a consequence of its common use as a reference, commercial cultivar, replicates were present within the Diascope orchard experimental design and it was included in the capture sequencing. The former three cultivars were homozygous for the allele associated to late budbreak and were selected as representatives of this genotype for subsequent analyses. Together, they will from now on be referred to as the late group. To perform the Tabuenca test, sufficient branches were collected from each cultivar to obtain a minimum 15 floral buds, on a weekly or fortnightly basis (due to fewer buds being available on some trees) and placed upright in containers with a regular supply of water. These were kept at 25°C for 7 days after which the buds were detached, scales removed and fresh weight taken of three replicates of five bud primordia. The date at which there was a 15% increase in mean bud fresh weight between successive samplings was considered the moment of transition from endodormancy to ecodormancy, as per Chuine et al. (2016). This date was identified through linear interpolation between the two sampling dates between which the first 15% weight increase was detected.

### 2.7 RNA extraction and gene expression analysis

In addition to the three cultivars of the late group, Gala and X2621 were also chosen for gene expression analysis. These two cultivars will from now on be referred to as the reference group and represent those homozygous for the reference A allele of the SChr09_680633 SNP. Bud sample collection for gene expression was carried out as follows: at four timepoints during endodormancy, specifically, December 16 (2022), January 26, February 22 and April 6 (2023), six floral buds were sampled from all replicates of each cultivar and immediately frozen in liquid nitrogen. To maximize the likelihood that sampled buds were floral, only those present on short bourse shoots and with a rounder appearance, a common characteristic of floral buds, were selected. Samples were later ground with a mortar and pestle in liquid nitrogen. Total RNA was extracted from 100 mg of ground tissue using the NucleoSpin^®^ RNA Plant kit (Macherey-Nagel, Düren, Germany). Synthesis of cDNA was performed using the SuperScript III First-Strand Synthesis System (Thermo Fisher Scientific, MA, USA). Expression of genes involved in the molecular control of the dormancy cycle and flowering was followed using RT-qPCR and these are listed in **Supplementary Table S3** with their primer sequences and efficiencies. The expression of MD09G1010000, which contains the SChr09_680633 SNP, was also followed. This gene is a peroxidase family gene and will now be referred to as *MdPRX10*. RT-qPCR was performed on the Roche LightCycler 480 instrument using SYBER Green (Roche, Basel, Switzerland) and consisted of 45 cycles and an annealing temperature of 60°C. Primer efficiencies were estimated with LinRegPCR software (v.2017.0; Ruijter et al., 2009). Relative gene expression was calculated using the $2^{-\Delta \Delta CT}$ method (Livak and Schmittgen, 2001). Target gene expression was normalized with the reference gene, *ACCUMULATION AND REPLICATION OF CHLOROPLAST5* (*MdARC5*; Perini et al., 2014). To test for significant differences (*p*-value ≤ 0.05) between cultivar expression levels within a timepoint and within a cultivar across timepoints, the emmeans R package (Lenth, 2022) was used to perform an ANOVA with Šidák adjustment to correct for multiple testing. All *p*-values for this analysis are given in **Supplementary Table S6**.

### 2.8 RNA-seq data from external studies

To validate RT-qPCR results of *MdPRX10* and determine its expression pattern during dormancy and in response to cold exposure, published RNA-seq datasets from two unrelated studies, Moser et al. (2020) and Takeuchi et al. (2018), were mined. RNA-seq expression data from Moser et al. (2020) was that of dormant terminal buds of Golden Delicious, sampled monthly from October until March, and thus spanning the period of dormancy until budbreak. In Takeuchi et al. (2018), RNA-seq libraries were generated from floral terminal buds of Fuji branches collected in late autumn, and subsequently incubated at 5°C in darkness for 0, 10, 25, 35 or 65 days.

### 2.9 DNA extraction and polymerase chain reaction

To examine the occurrence of transposons within the *MdFLC-like* gene, four cultivars were selected: Gala and the three of the late group. Young leaves were collected from one replicate of each cultivar in June 2020, immediately frozen in liquid nitrogen and then lyophilized for storage at -20°C. Genomic DNA was extracted using the DNeasy Plant Maxi kit (Qiagen, Hilden, Germany). A series of PCR primers were designed to span the length of the apple *MdFLC-like* gene, as given in the GDDH13 genome assembly (Daccord et al., 2017), using Primer3web v.4.1.0 (https://primer3.ut.ee; Rozen and Skaletsky, 2000) and the NCBI Primer-BLAST tool (https://www.ncbi.nlm.nih.gov/tools/primer-blast), to verify specificity. Primer positions and sequences are given in **Supplementary Figure S2** and **Supplementary Table S3**, respectively. PCRs were carried out using KOD Hot Start DNA polymerase (Sigma Aldrich, MO, USA) and undiluted DNA of each cultivar. Thermocycling conditions were as follows: activation at 95°C for 2 m; denaturing at 95°C for 20 s; and extension at 70°C for 25 s/kb, for 35 cycles. To determine any DNA fragment size differences due to the presence or absence of transposons, PCR products were run on a 1.4% agarose electrophoresis gel and visualized under UV light following staining in a 1 µg/mL ethidium bromide solution.

### 2.10 Protein structural studies

Protein sequences from members of the orthogroup to which MdPRX10 belongs (Orthogroup Prx31) were obtained from the peroxidase-specific database, Peroxibase (https://peroxibase.toulouse.inra.fr/orthogroups/view_orthogroup/Prx031). The protein sequence alignments were performed using the Clustal Omega tool (www.ebi.ac.uk) and configured with default parameters. Resulting protein sequence alignments were visualized using the MView tool on the same platform.

An initial study of the MdPRX10 orthogroup proteins was undertaken, and protein structures were predicted using the I-TASSER server (https://zhanggroup.org/I-TASSER). For MdPRX10, two different structures were predicted: one for MdPRX10-Phe156 and the other for MdPRX10-Tyr156. Hydrogen bond analysis was performed for both proteins between residue 156 and residues located in a 3.0 Å range. In addition, the CUPSAT server (https://cupsat.brenda-enzymes.org) was used to predict the effect of the residue change on protein stability, and total free energy was calculated using the Force Field method to assess the viability of both proteins.

To analyze the effect of the variation Phe156 to Tyr156 in the dimerization capabilities of MdPRX10, dimerization events were computed for both structures using the protein-to-protein docking software PyDock (https://life.bsc.es/pid/pydock). Dimerization models were then represented using PyMol (https://pymol.org/2).

## 3 Results

### 3.1 A capture sequencing approach increases available polymorphisms in a French apple core collection

Previous genetic studies have been unable to unambiguously identify genes related to dormancy and budbreak control within the QTL at the top of LG9 in apple. This is likely due, in part, to the lack of data on the DNA sequence of candidate genes in the studied cultivars in this region. These data could assist in the identification of polymorphisms within their coding sequences or regulatory regions that have an effect on the phenotype. In order to overcome this limitation, a target capture sequencing approach was implemented to obtain information on the coding sequence of potential candidate genes, including approximately 200 genes within the LG9 QTL (Trainin et al., 2016) and apple orthologs of known *A. thaliana* flowering time related genes (Bouché et al., 2016). A total of 275 377 104 paired end reads of 250 nucleotides were generated from Illumina Hiseq2500 for 284 samples (mean per sample: 969 637.7, standard error: 28 034.4). Seven samples with a very low read depth were removed from the analysis. Notably, 261 523 806 (94.97%) of these reads were mapped successfully to the reference genome, 233 610 014 (89.33%) of which remained after PCR/optical duplicate removal. After variant filtration, 116 296 polymorphisms were selected within the sequences of around 1072 apple gene *loci* (**Supplementary Table S4**).

### 3.2 Timing of budbreak showed a strong genetic component

**Figure 1:**
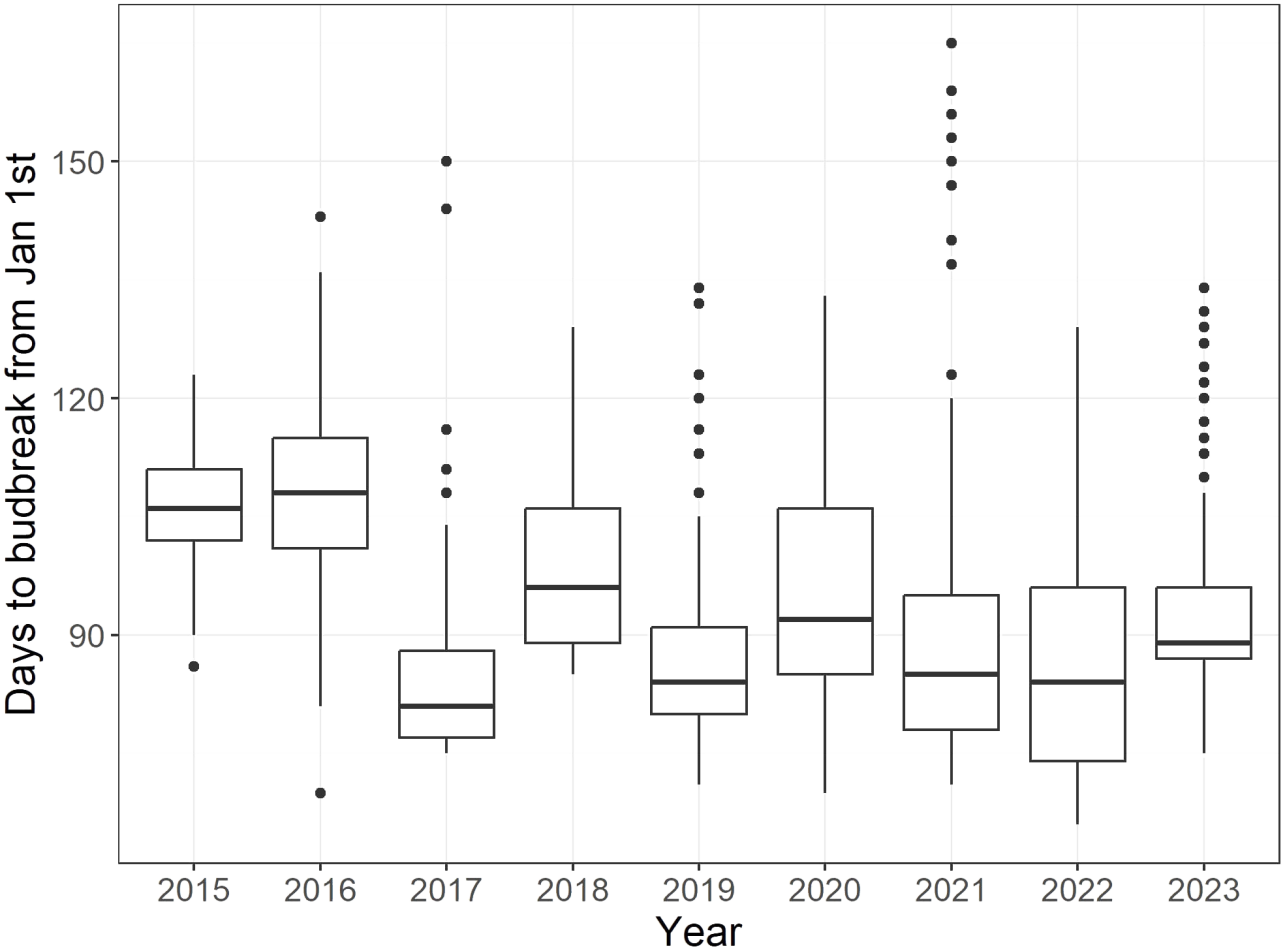
Boxplots representing days to budbreak from January 1^st^ for all 239 cultivars of the core collection from 2015 to 2023. Each boxplot includes the data of all four replicates (i.e. raw data) of each cultivar, with the exception of 2015 and 2016, where two replicates were recorded.

In the first two years following planting out, 2015 and 2016, the mean budbreak was 107 and 108 days, respectively, notably later than those of other years, which ranged from 83-99 days (**Figure 1**). In general, the budbreak period was relatively variable across years in the collection, ranging from 37-94 days. The contribution of year, genotype and their interaction to the date of budbreak were all highly significant (*p*-value< 0.001).

**Figure 2:**
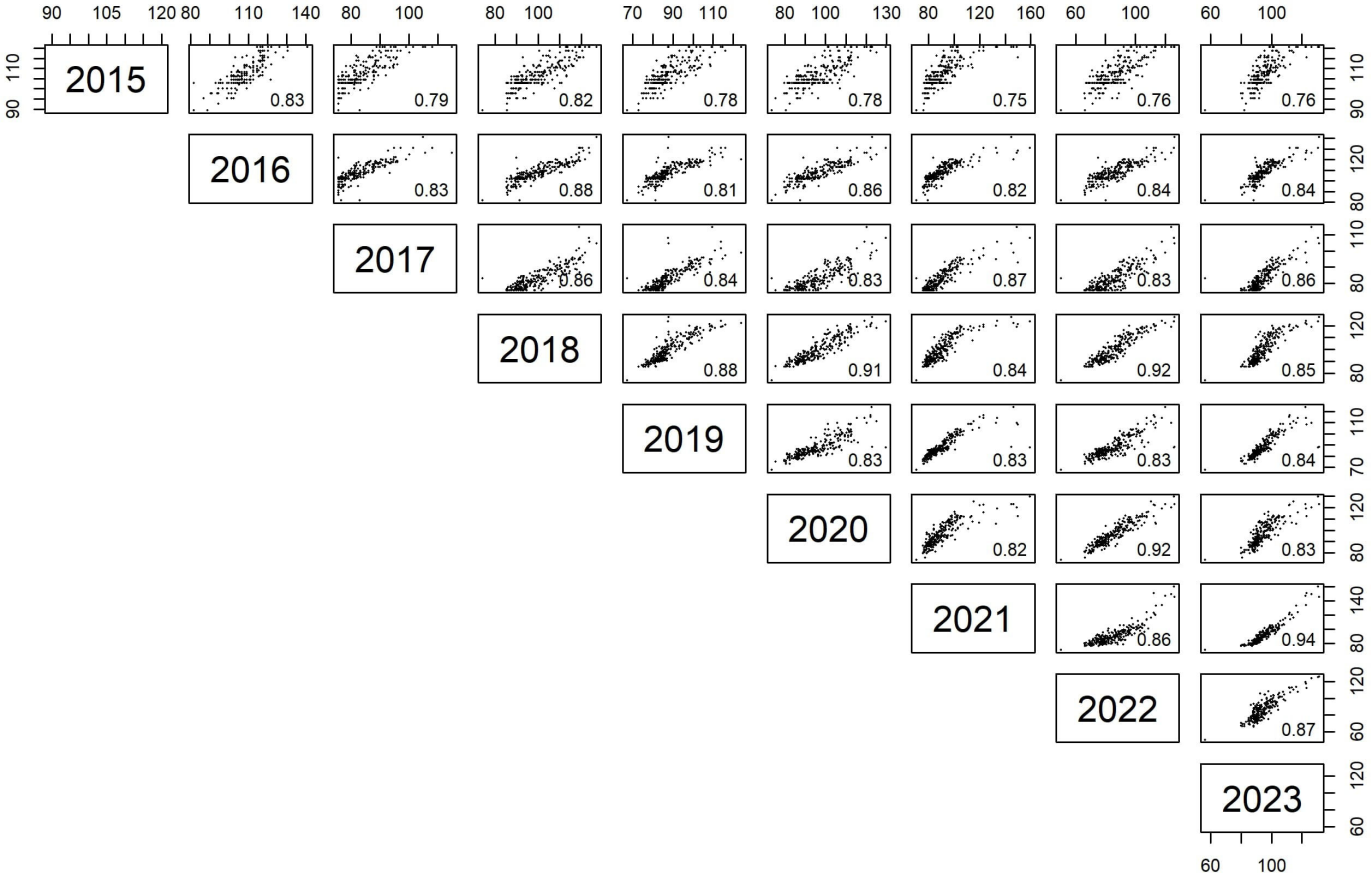
Pairwise scatter plots and Pearson correlation coefficients (r values) of budbreak timing between genotypic BLUPs of each year from 2015 to 2023 in the apple core collection.

Although mean budbreak varied between years, phenotypic correlations between all years were high (r = 0.75 - 0.94; **Figure 2**). The $H^{2}$ , calculated across all years, was estimated at 0.87 (95% confidence interval of 0.84-0.89) and narrow-sense heritability ( $h^{2}$ ), calculated using the G BLUPs, was 0.99.

### 3.3 A locus on LG9 showed a strong association with timing of budbreak

**Figure 3:**
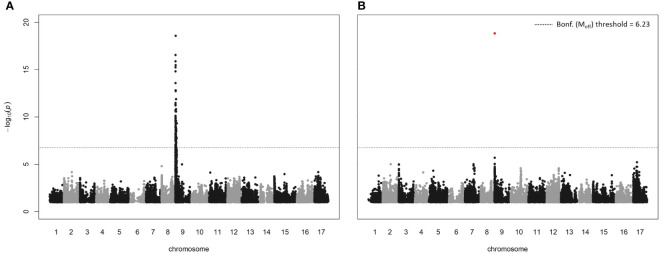
Manhattan plots from GWAS analyses on timing of budbreak using **(A)** a single-locus method, GEMMA, and **(B)** a multi-locus approach, MLMM. Bonferroni (Bonf.) threshold was calculated using the effective number of independent tests (M_eff_).

Both the GEMMA and MLMM GWAS methods detected a strong association between timing of budbreak in the core collection and a region on LG9 (**Figure 3**; further output given in **Supplementary Figure S3**; all significant associations are reported in **Supplementary Table S5**), coinciding with a QTL identified by previous studies on apple (Conner et al., 1998; van Dyk et al., 2010; Celton et al., 2011; Allard et al., 2016; Urrestarazu et al., 2017; Miotto et al., 2019; Cornelissen et al., 2020) and pear (Gabay et al., 2017; Ntladi et al., 2018). The most significant SNP in both the analyses was SCh09_680633, which originated from the capture SNP dataset and is located within the peroxidase superfamily gene, *MdPRX10*, near the top of LG9 (679 939 – 681 272 bp; -log_10_(*p*-value) = 19.74). While the genotypic dataset contained 20 SNPs located within this gene, only SCh09_680633 was significant. Calculation of the linkage disequilibrium between this SNP and all other SNPs within the QTL interval (the region between the first and last SNP with a significant *p*-value; 254 255 bp to 3 509 888 bp) revealed no r^2^ above 0.8 (**Supplementary Figure S4**), the generally accepted threshold of high linkage disequilibrium (Broekema et al., 2020). The SCh09_680633 SNP is a A/T polymorphism that results in an amino acid substitution of phenylalanine (Phe) to tyrosine (Tyr) at position 156 (Phe156 -> Tyr156), the latter of which had an estimated effect of +9.43 days on the timing of budbreak (GEMMA analysis). Within the core collection, eight cultivars were homozygous for the T allele of the SCh09_680633 SNP, 45 cultivars were heterozygous and the remainder were homozygous for the reference A allele. The percentage of genotypic variance explained by the SNP was estimated as 30% and 43% by the GEMMA and MLMM analyses, respectively. The protein encoded by this gene is a class III peroxidase and was assigned the annotation MdPRX10 by the RedOxiBase database (https://peroxibase.toulouse.inra.fr).

**Figure 4:**
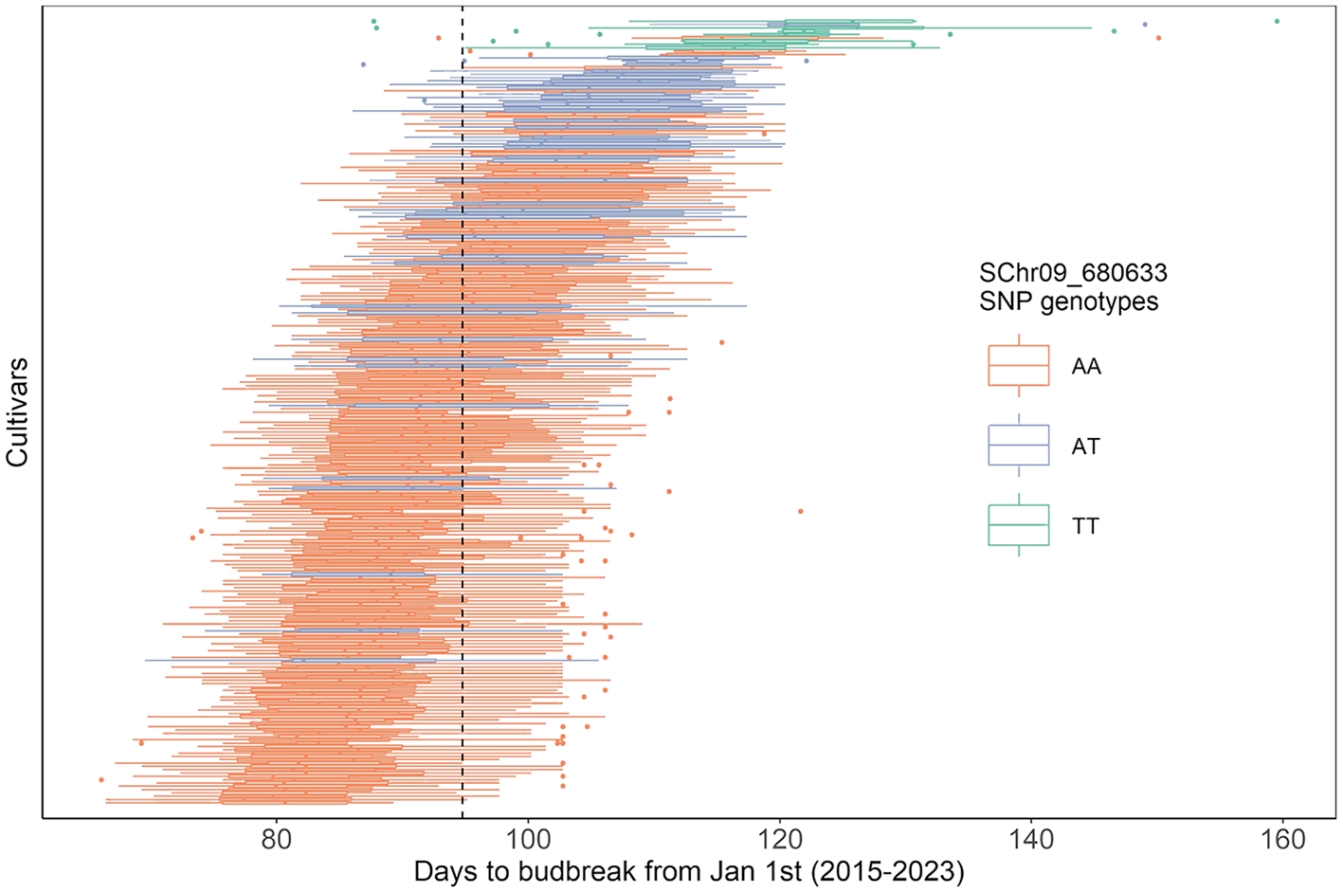
Stacked boxplots of genotypic BLUPs of days to budbreak from January 1^st^ of each cultivar of the core collection, within each year from 2015 to 2023 (i.e. nine BLUPs per cultivar). Plots are ordered by mean from earliest to latest budbreak. Color indicates the SChr09_680633 SNP alleles present in the *MdPRX10* gene, specifically, homozygous for the reference allele, A (orange; 186 cultivars), homozygous for the alternative T allele (green; 8 cultivars) or heterozygous with a copy of each (blue; 45 cultivars). Dashed line is the mean budbreak date across all years and cultivars (94 days).

The eight cultivars homozygous for the late budbreak-associated T allele of the SCh09_680633 SNP were consistently some of the latest cultivars in the collection (**Figure 4**). The heterozygous cultivars also tended to be later than the mean budbreak of the population, although this was more variable in these cultivars. Nevertheless, the G BLUPs calculated from all years together showed a highly significant difference in the budbreak timing between all the SNP allelic combinations (*p*-value< 0.0001). This relationship between the SCh09_680633 SNP genotype and late budbreak was consistent across all years (**Supplementary Figure S5**).

### 3.4 Endodormancy and/or ecodormancy were affected in late cultivars

**Figure 5:**
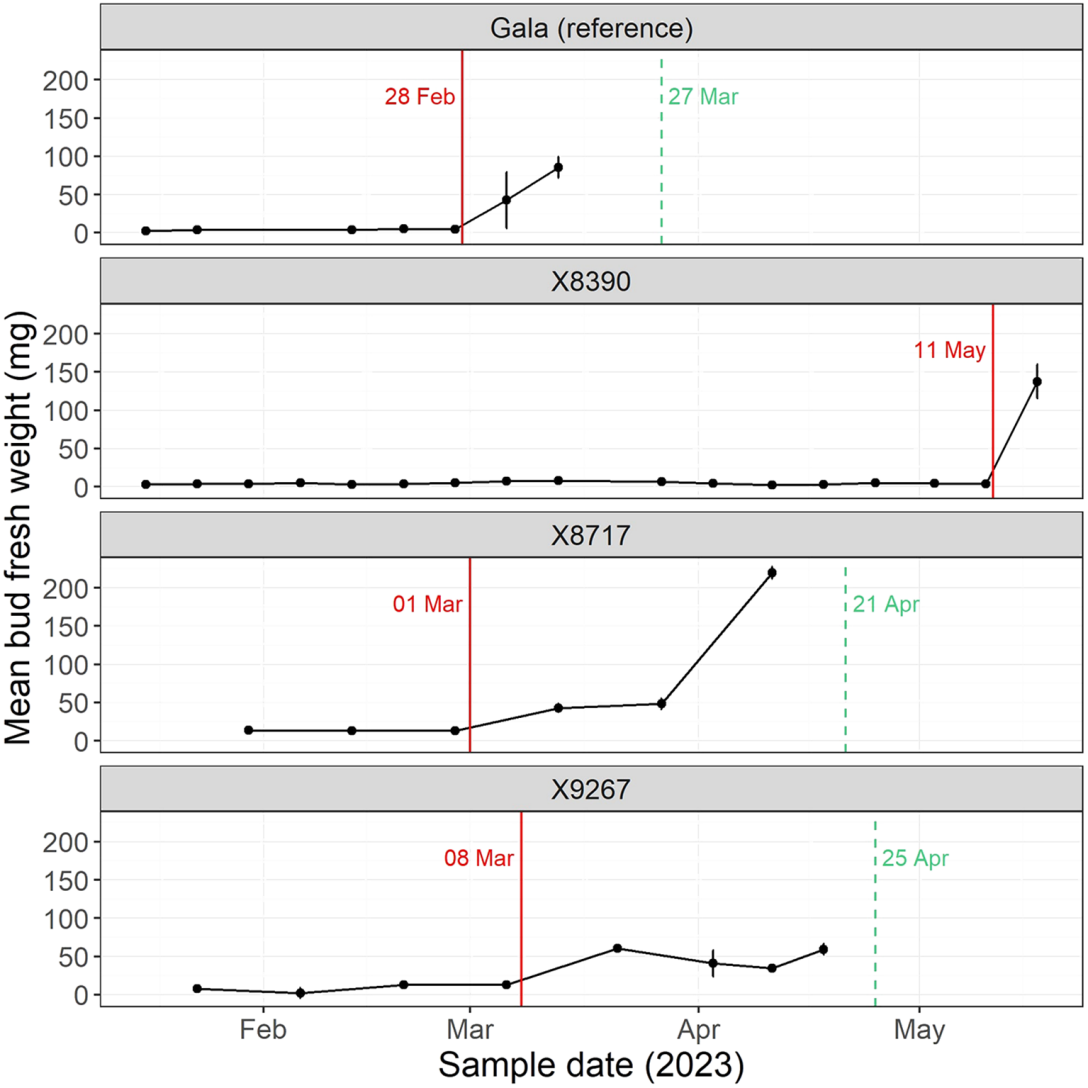
Mean bud fresh weight (scales removed) following forcing conditions, in accordance with the Tabuenca test in 2023. Gala is a reference cultivar (AA), while X8390, X8717 and X9267 are all late cultivars (TT). The red line indicates the date at which bud fresh weight had increased by 15% since the previous sampling, thus signaling the end of endodormancy as described by Chuine et al. (2016). The dashed green line indicates the mean date of budbreak of the four cultivar replicates in the field at the Diascope experimental orchard in 2023. Field budbreak in cultivar X8390 was highly desynchronized and so a single date for budbreak was unable to be determined. Vertical error bars are the standard error of the mean of three replicates.

The Tabuenca test was performed to determine which aspect of dormancy was affected in the late budbreak cultivars, endodormancy and/or ecodormancy. The test identified the date of endodormancy end and, with the timing of the in-field budbreak, the duration of ecodormancy could also be estimated. The level to which either dormancy contributed to the late budbreak varied between the three cultivars of the late group, X8390, X8717 and X9267 (**Figure 5**). Endodormancy of X8390 was more than two months longer than the other two cultivars, with budbreak finally occurring in May (1099 CH), although determination of ecodormancy duration was less clear. Several replicates of this cultivar had low numbers of floral buds as well as highly desynchronized budbreak, which hampered the ability to apply a specific date to budbreak in the field. The timing of the end of endodormancy in X8717 (914 CH) was similar to Gala (899 CH), while X9267 (989 CH) was slightly later. However, the length of ecodormancy in both these cultivars was approximately double that of Gala, resulting in the later budbreak.

### 3.5 *MdPRX10* shows dormancy- and cold-related expression patterns

**Figure 6:**
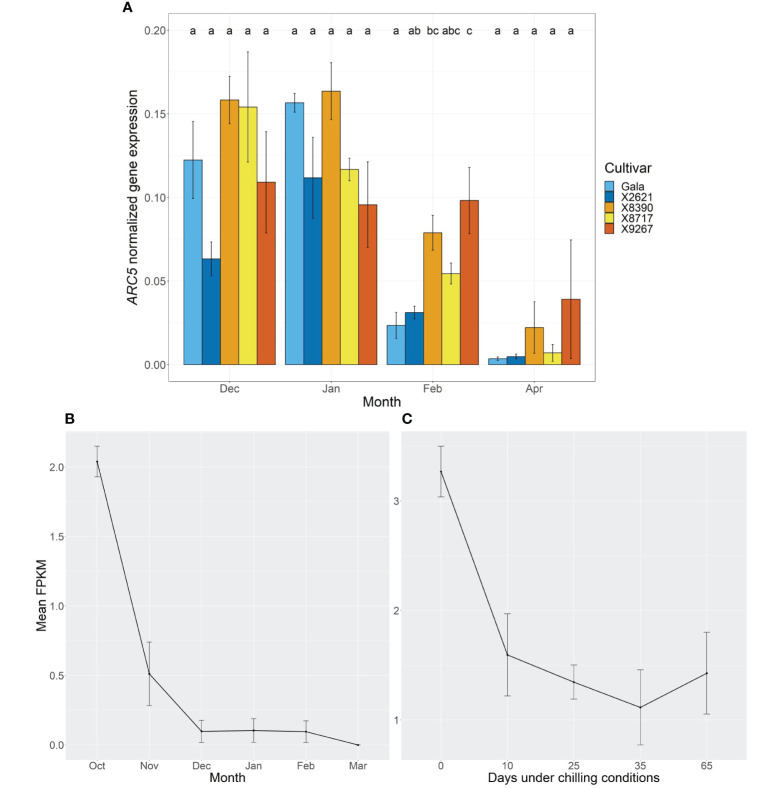
**(A)** *ARC5*-normalized expression of *MdPRX10* in bud tissue of two cultivars homozygous for the A allele at the SChr09_680633 SNP locus, Gala and X2621 (reference group, blue hues), and three cultivars homozygous for the T allele, X8390, X8717 and X9267 (late group, yellow-red hues). Sampling was carried out monthly from December 2022 to February 2023 and in April 2023. Bars represent mean normalized expression of three biological replicates. Error bars represent the standard error of the mean. Different letters within a month indicate a significant difference (*p*-value ≤ 0.05) between cultivars. **(B)** Mean Fragments Per Kilobase of transcript per Million mapped reads (FPKM) of *MdPRX10* from Golden Delicious terminal, floral buds sampled monthly from October to March. Error bars represent standard error of the mean of two biological replicates. Data extracted from published RNA-seq results from Moser et al. (2020). **(C)** Mean FPKM of *MdPRX10* from Fuji terminal, floral buds sampled as branches in November and subjected to no chilling (0 days), 10, 25, 35 or 65 days under chilling conditions (5°C). Error bars represent standard error of the mean of 2-3 biological replicates. Data extracted from published RNA-seq results from Takeuchi et al. (2018).

To examine the potential role of *MdPRX10* in budbreak, its expression was followed with RT-qPCR at four timepoints over the dormancy period. A common expression pattern was observed across both groups over this time (**Figure 6A**). Expression in January was significantly higher (*p*-value ≤0.05) than in April for all cultivars, with the exception of X9267, which had highly variable expression between replicates at this timepoint. Expression in April was generally very low for all cultivars. In the first two months and the final month, there were no differences in expression between cultivars, while in February, X9267 had significantly higher expression than the reference cultivars, with X8390 also showing higher expression than Gala. However, there was no definitive trend between the late and reference groups. All *p*-values from expression comparisons are given in **Supplementary Table S6**. A similar global expression pattern was evident in Golden Delicious floral bud RNA-seq data, published in Moser et al. (2020) (**Figure 6B**). Here, initial expression of *MdPRX10* in October, the probable beginning of endodormancy, was followed by a rapid fall in expression, continuing until the gene was unexpressed or only lowly expressed from December until budbreak in March. In RNA-seq data of Fuji, this progression of decreasing expression of *MdPRX10* was also observed in relation to cold exposure (**Figure 6C**; Takeuchi et al., 2018). Relatively high expression of *MdPRX10* occurred without chilling, however, it continued to decrease following longer durations under these conditions, reaching its lowest expression after 35 days at 5°C.

### 3.6 *MdFLC-like* might not be involved in the late budbreak phenotype

**Figure 7:**
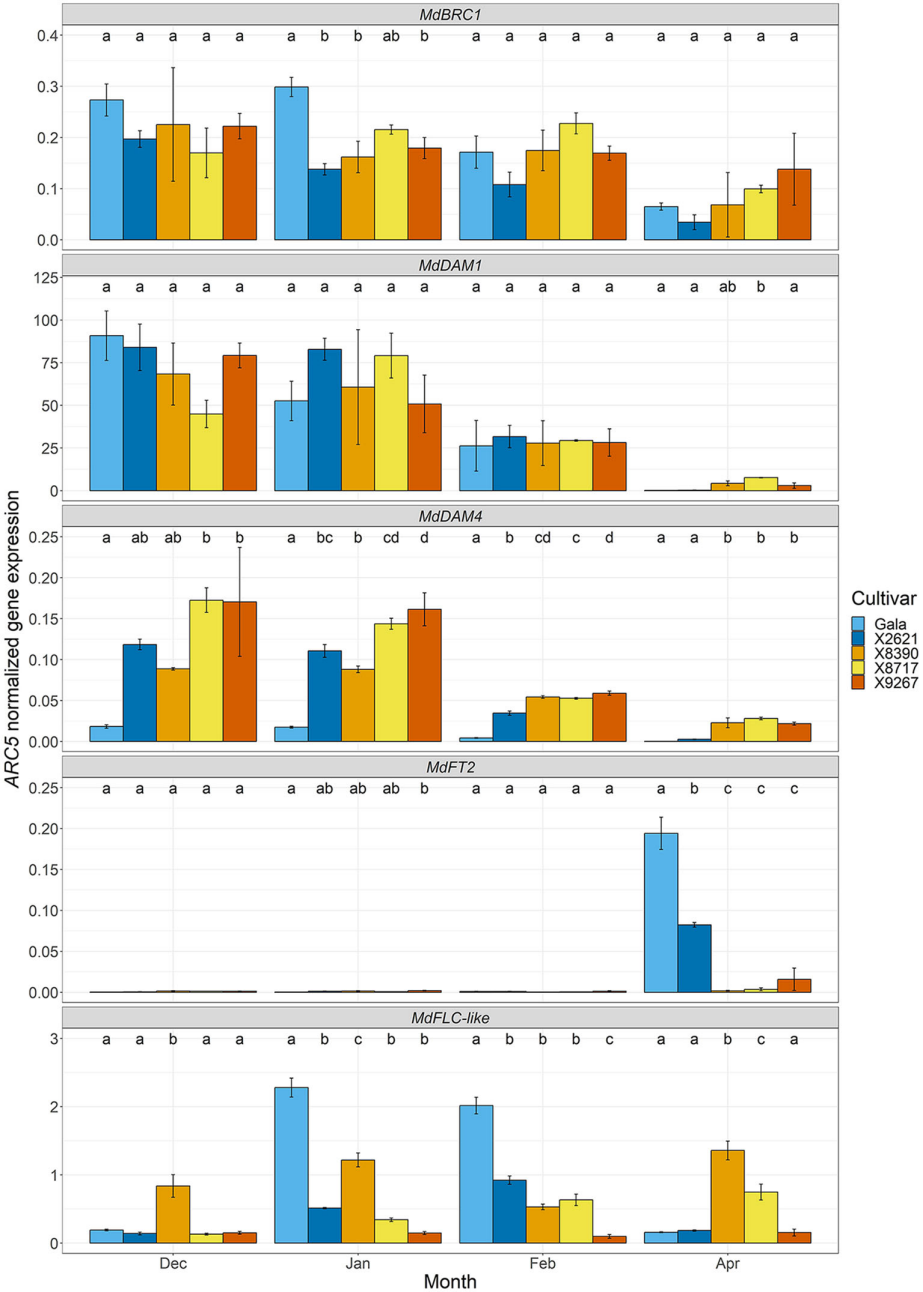
*ARC5*-normalized expression of dormancy cycle and flowering-related genes: *MdBRC1*, *MdDAM1*, *MdDAM4*, *MdFT2* and *MdFLC-like* in bud tissue of two cultivars homozygous for the A allele of the SChr09_680633 SNP, Gala and X2621 (reference group, blue hues), and three cultivars homozygous for the T allele, X8390, X8717 and X9267 (late group, yellow-red hues). Sampling was carried out monthly from December 2022 to February 2023 and in April 2023. Bars represent mean of three biological replicates. Error bars represent the standard error of the mean. Different letters within a month indicate a significant difference (*p*-value ≤ 0.05) between cultivars.

The expression of several dormancy cycle and flowering-related genes was followed, using RT-qPCR, over the course of dormancy to understand how underlying processes may differ in late budbreak cultivars and whether any could be directly involved in a mechanism involving *MdPRX10* (**Figure 7**). Expected dormancy related expression patterns were generally observed in *MdBRC1* and *MdDAM1* and *MdDAM4*, with down-regulation towards the end of dormancy, however as budbreak was delayed in the late cultivars, this reduction in expression occurred slightly later. *MdBRC1* expression dropped significantly in the reference cultivars between January and April, and although there may have been a similar trend in the late cultivars, it was not significant. *MdDAM1* and *MdDAM4* showed significantly lower expression in all cultivars by April (with the exception of X8390 in the case of *MdDAM1*) and while *MdDAM4* expression in the late cultivars was significantly higher than the reference group in April, the expected expression pattern was clear. Furthermore, the expression of *MdFT2*, a likely budbreak activator after dormancy (Lempe et al., 2022), was significantly higher in April in the reference cultivars compared to the late, in agreement with the delayed budbreak phenotype of the late group. Since *FT* is known to be transcriptionally regulated by FLC in *A. thaliana*, the expression of *MdFLC*-*like* was also investigated. This gene was also of particular interest owing to its known involvement in cold-mediated bud dormancy and location close to *MdPRX10* on LG9. The expression pattern of *MdFLC-like* in the reference cultivars approximately followed the expected pattern for a floral repressor that is responsive to temperature (Porto et al., 2015; Nishiyama et al., 2021), with a significant peak in expression during cold temperatures when the buds were dormant (January and February) and a lowering of expression as the temperatures increased towards final budbreak. This pattern was not so evident in two of the late cultivars, X8390 and X9267, which did not show this peak in expression. Despite not possessing any significant SNPs in the GWAS analyses or being in high linkage disequilibrium with SCh09_680633, the *MdFLC-like* gene is very close to *MdPRX10* and any potential influence of it in this QTL needed to be thoroughly investigated. Thirty-four SNPs within this gene were present in the dataset, so that with the lowest *p*-value from the GEMMA GWAS analysis (AX.115485819, -log_10_(*p*-value) = 2.73) was chosen to compare the budbreak phenotype between the various allelic genotypes (**Supplementary Figure S6**). This indicated no clear tendencies between cultivars with the various allelic combinations of this SNP with respect to budbreak timing.

Finally, to verify whether the function of *MdFLC-like* was potentially affected by the presence of transposable elements, of which the apple genome contains many (Daccord et al., 2017), the size of PCR products spanning the length of the gene, including regulatory regions, was compared between Gala and the cultivars of the late group. Visualization of the DNA fragments on a gel indicated they were each of a similar size in all cultivars, suggesting there are not significant changes in transposable element number or length. Gel photos are given in **Supplementary Figure S7**.

### 3.7 Expression of cold-receptive genes was up-regulated in late cultivars

**Figure 8:**
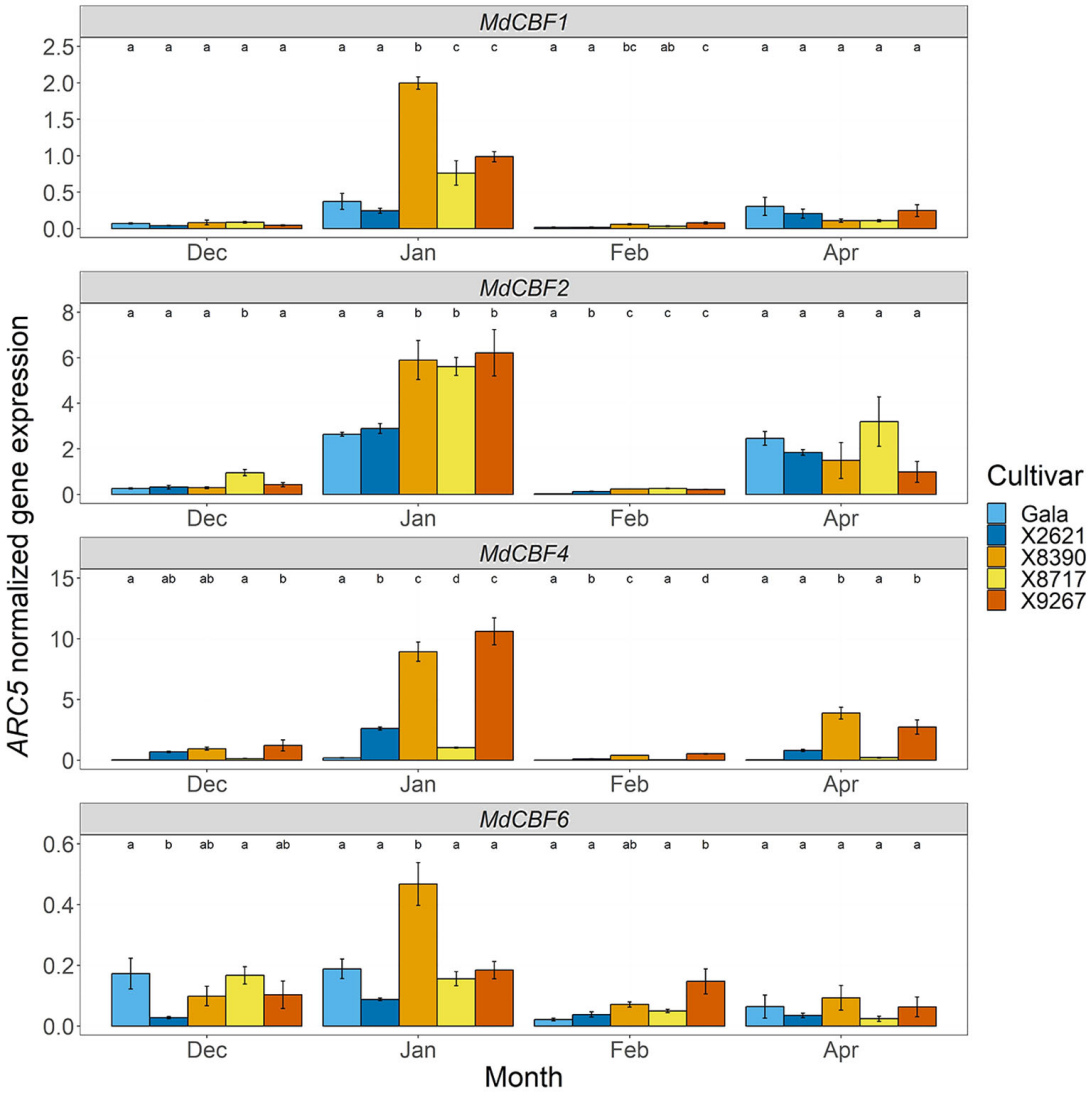
*ARC5*-normalized expression of *MdCBF1*, *MdCBF2*, *MdCBF4* and *MdCBF6* in bud tissue of two cultivars homozygous for the A allele of the SChr09_680633 SNP, Gala and X2621 (reference group, blue hues), and three cultivars homozygous for the T allele, X8390, X8717 and X9267 (late group, yellow-red hues). Sampling was carried out monthly from December 2022 to February 2023 and in April 2023. Bars represent mean of three biological replicates. Error bars represent the standard error of the mean. Different letters within a month indicate a significant difference (*p*-value ≤ 0.05) between cultivars.

As dormancy and flowering are closely connected to chilling requirement, expression of genes involved in cold perception was also examined. *MdCBF1*, *MdCBF2*, *MdCBF4* and *MdCBF6* showed differential expression at several timepoints between the late and reference groups (**Figure 8**). A peak in expression levels in January was clearest in *MdCBF1* and *MdCBF2* and, while also present in some cultivars in *MdCBF4* and *MdCBF6* expression, it was more variable. During this peak in January, the late group cultivars had significantly higher expression of *MdCBF1* (4-fold mean increase) and *MdCBF2* (2-fold mean increase) than the reference group. This also occurred in X8390 and X9267 with *MdCBF4*, and in X8390 with *MdCBF6*. These two cultivars maintained significantly higher expression levels of *MdCBF1*, *MdCBF2* and *MdCBF4* into February, and in the case of *MdCBF4*, into April. The other member of the late group, X8717, maintained higher expression than the reference group into February in only *MdCBF2*. The expression of *MdICE1* (LG9, 335 088 – 338 411 bp) was also quantified as it encodes a transcription factor that was proposed to regulate the transcription of *MdCBF* genes during dormancy progression (Miotto et al., 2019) and is located near the LG9 QTL interval. No clear differences in *MdICE1* expression levels were observed between late and early cultivars (**Supplementary Figure S8**). Altered expression of *CBF* genes could have a downstream effect on budbreak timing due to modification of chilling perception.

### 3.8 The MdPRX10 protein function may be perturbed in late cultivars

**Figure 9:**
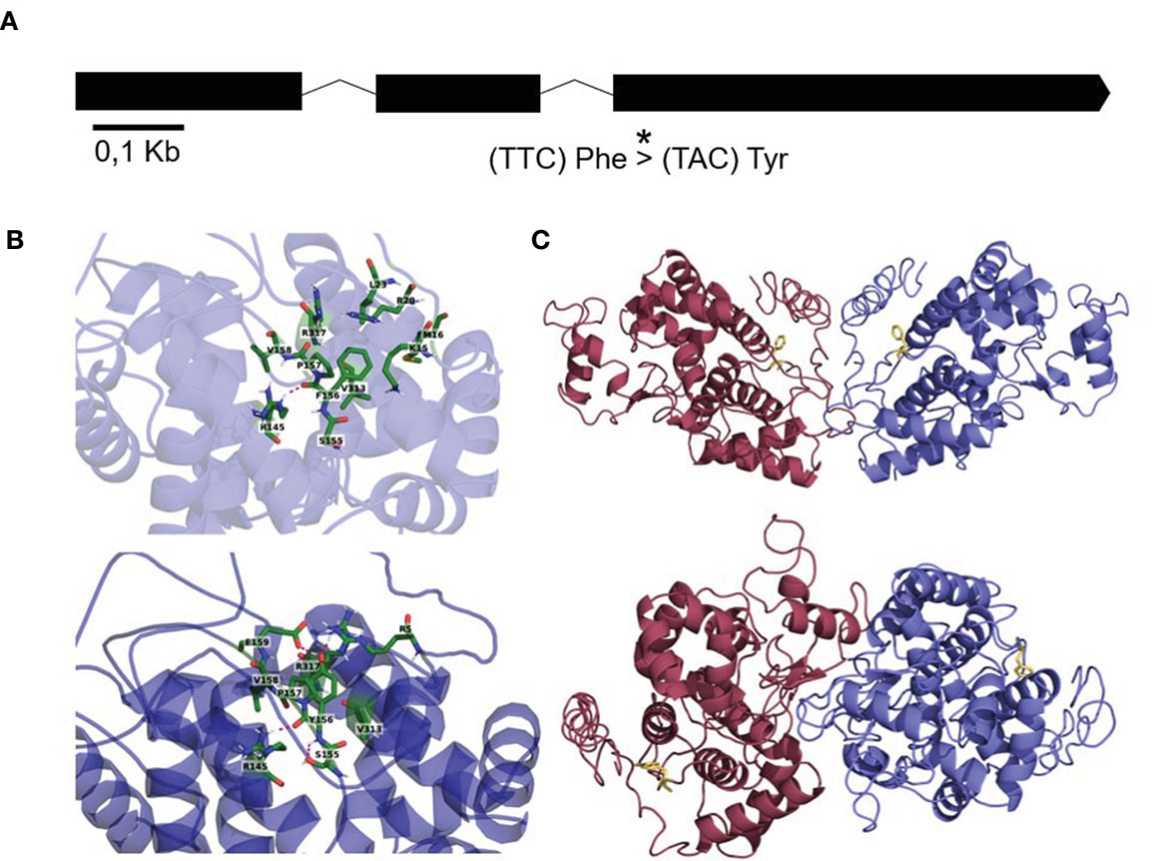
**(A)** Layout of the *MdPRX10* gene showing exons (thick lines) and introns (thin lines) and the position of the SCh09_680633 SNP (*), which causes the change from a Phe156 residue to a Tyr156 residue in the MdPRX10 protein **(A)**. **(B)** A zoomed schematic of the location on the MdPRX10 protein affected by the residue change and the resulting surrounding residue positioning in the presence of Phe156 (above) or Tyr156 (below). **(C)** Dimer configuration of MdPRX10 proteins (one blue, one red) in the case of the Phe156 residue (above) and in the case of the Tyr156 residue (below). The affected residue is shown in yellow.

A multispecies alignment of MdPRX10 orthologs indicated that Phe156 (F156) was the most frequent residue at this position (54%) within 91 protein sequences from 58 species. Tryptophan (Trp) (38%) and Tyr (8%) were also found in some protein sequences at this position (**Supplementary File 1**). We performed several computational simulations to estimate the potential effect of the Phe156 to Tyr156 variation on the MdPRX10 protein (**Figure 9A**). The 3D structure of the MdPRX10-Phe156 protein showed the formation of a hydrogen bond between Phe156 and a proximal Arg145 (**Figure 9B**). The MdPRX10-Tyr156 showed a formation of two hydrogen bonds between Tyr156 and an Arg317, as an electron acceptor, and a Glu159, as an electron donor. Application of the Force Field method showed that the structural energy of MdPRX10-Phe156 (-12049.269 kJ/mol) was slightly more negative than that of MdPRX10-Tyr156 (-12021.819 kJ/mol), indicating a destabilizing impact of the F156Y modification on MdPRX10. This change in protein stability is also confirmed through the CUPSAT prediction (**Supplementary Table S7**). Nonetheless, since both MdPRX10-Phe156 and MdPRX10-Tyr156 proteins present an overall negative free energy, a destabilizing point mutation would probably not affect protein assembly and thus, viability. Next, the ability of MdPRX10 protein alleles to dimerize was tested *in silico*. The performed simulations suggested Phe156 is involved in most of the dimerization models, which is not the case when this residue is replaced by Tyr156 (**Figure 9C**). This tendency was also observed in peroxidase proteins from other plant species (**Figures S9A-D**). Furthermore, the dimerization was affected in simulated heterocomplexes formed by Phe156 and Tyr156 proteins, a configuration that recreates the situation occurring in individuals heterozygous for this residue (**Supplementary Figure S9E**). The presence of the Tyr156 residue was not, however, thought to be large enough to prevent dimer formation but rather the change in conformation of the dimer may impact subsequent function of the homodimer.

## 4 Discussion

### 4.1 Capture sequencing enabled enhanced resolution of genes within the LG9 QTL interval and identification of *MdPRX10* as a key candidate

The ubiquitous nature of the LG9 QTL strongly hints at its importance within dormancy and budbreak regulation. However, until now, the attribution of ‘candidate’ to genes within the interval has principally been given based on the known or assumed role of certain genes in dormancy or flowering processes and their location in or near the QTL interval. Here, we took a targeted enrichment approach with respect to gene coding sequences, using capture sequencing, which increased the resolution of markers within genes known or suspected to be involved in flowering-time control in *A. thaliana* (Bouché et al., 2016), as well as approximately 200 genes located within the QTL itself (Trainin et al., 2016). The subsequent GWAS analyses were further strengthened by genotypic values derived from nine years of budbreak phenotypes from a large apple core collection and the use of MMLM, which accounts for the signal of other SNPs that are significant due to population structure (Segura et al., 2012). Accounting for the G:Y effect in the BLUPs model also allowed a focus on the stable aspect of this QTL, which occurs across years, populations and traits in the literature. This led to the identification of the peroxidase superfamily gene, *MdPRX10*, as a robust candidate underlying the LG9 QTL within this core collection. Specifically, the capture-derived SChr09_680633 SNP was of particular initial interest in this gene due to both the method of its discovery and the strength of its association to budbreak timing. The culmination of these approaches provides the most solid evidence to date for a candidate gene within the LG9 QTL and supports further efforts in ascertaining its specific role in the budbreak mechanism.

### 4.2 *MdPRX10* appears involved in dormancy release and responds to chilling

Beyond the clear statistical association of *MdPRX10* with budbreak, analysis of its expression profile in other RNA-seq studies supports a role in dormancy. Such data from Moser et al. (2020) showed a decreasing expression pattern over the course of dormancy, which reached its lowest levels at budbreak. We validated this dynamic by RT-qPCR, showing *MdPRX10* expression reduced as dormancy progressed for all but one, particularly variable cultivar. This may be due to chilling having a down-regulating effect on *MdPRX10*, as indicated by the RNA-seq data of Takeuchi et al. (2018), where expression was reduced in buds following increasing durations at low temperature. This may suggest a role in maintaining dormancy until a certain amount of exposure to cold temperatures is reached, in a similar vein to *MdDAM1* and *MdDAM4* (Falavigna et al., 2019; Vimont et al., 2019). In the current study, these two *DAM* genes also showed this expected expression pattern, as well as *MdBRC1*. *MdBRC1* and *MdDAM1* showed no clear differences between the late and reference groups, while *MdDAM4* showed higher expression in the late cultivars than the reference from February, which could correlate with its potential function as a budbreak repressor. Furthermore, low expression of *MdFT2* in the late cultivars in April compared to the reference cultivars, which also coincides with their late budbreak phenotype. Whether these differences are consequence of upstream effects by MdPRX10 on *MdDAM4* and *MdFT2* expression will be discussed further below.

Another dormancy-related gene, *MdFLC-like*, has been a popular candidate in other studies within the LG9 QTL. However, despite the presence of several capture SNPs within the gene, we detected no significant signal in the GWAS analyses and the expression pattern failed to indicate any clear differences between the reference and late cultivar groups. This is an undoubtably important gene in apple dormancy (Porto et al., 2015; Nishiyama et al., 2021), however we believe it is not a significant contributor to late budbreak in this core collection. Despite focusing on *MdPRX10*, we do not rule out other potential candidate genes underlying the LG9 QTL, especially considering the occurrence of several key dormancy-related genes in the region and the varying effects of the QTL in other studies, but these may be of smaller effect or depend on a polymorphism that only occurs in some populations. Indeed, there are many factors that influence the detection of QTLs in GWAS analyses, including genetic background of the population, population size, marker density, trait complexity, environmental interaction with the trait, etc. Potentially with a larger population or data from more diverse environments other minor QTL could be detected in the future.

### 4.3 MdPRX10 protein function may be altered by the SChr09_680633 SNP

In addition to potential effects on gene expression, our *in-silico* protein analysis indicated MdPRX10 function may be affected by the amino acid residue change caused by the SChr09_680633 SNP. Dimer formation has been shown to have significant effect on the stability and formation of the binding pocket in a similar protein in palm tree (*Chamaerops excelsa*; Bernardes et al., 2015), suggesting this process may also be important for MdPRX10. While the Phe to Tyr residue change was not predicted to prevent dimer formation, the conformation of the dimer was expected to be significantly altered, which could significantly alter ligand binding. The significance of this residue change is illustrated in its conservation across MdPRX10 orthologs in other species (Orthogroup Prx31). Within these orthologs across 58 species, the Phe residue is the most common at this position (54%), however, Tyr occurs with a frequency of 8%, indicating it is not a rare occurrence in this type of protein and may carry an important function. Further investigation to identify the binding target(s) of MdPRX10 would greatly aid in the understanding of both the effect of this residue change and the protein function.

### 4.4 Cold perception ability may underlie the late budbreak phenotype observed in this population

Transcription of *CBF* genes showed several clear differences between the late and reference cultivars, with higher expression evident in the late cultivars, especially in January, indicating altered cold sensitivity. *CBF* genes are involved in cold perception and tolerance in apple, as demonstrated by the over-expression of a peach *CBF* (*PpCBF1*) gene in apple, which resulted in early entry into dormancy and poor cold acclimation (Wisniewski et al., 2011). These genes may act via direct interaction with the promotor regions of *DAM* genes (Mimida et al., 2015; Wisniewski et al., 2015) and while a correlation with these genes was not evident here, a number of *COR* genes are thought to be activated by *CBF* genes within this pathway (Chinnusamy et al., 2003). The six *CBF* genes in *A. thaliana* are regulated each by specific transcription factors and hormonal signals (Wisniewski et al., 2014; Wi et al., 2022). For example, *AtICE1* is a transcription factor that was found to regulate transcription of *AtCBF3* (Chinnusamy et al., 2003), although this is still unclear in apple. Here, we looked at the expression of an apple *MdICE1-like* gene, located near the LG9 QTL (Miotto et al., 2019), and did not observe any difference in expression between late and reference cultivars. Other regulatory factors of *CBF* genes in apple remain to be explored and could point to how up-regulation of these genes fits within the late budbreak phenotype, potentially linking it to redox status. Recently, a mechanism including redox signaling and *CBF*-mediated cold tolerance in *A. thaliana* was described with respect to post-translational changes to CBF proteins via oxidative bursts, triggered by low temperatures, and leading to activation of downstream *COR* genes (Lee et al., 2021; Wi et al., 2022). While this is a mechanism by which the plant can rapidly respond to a cold snap, rather than the gradual descent into endodormancy in response to seasonal change, it illustrates how ROS and their associated enzymes, such as peroxidases, could be an effective regulator of the plant response to external temperature, as with dormancy.

### 4.5 *MdPRX10* may influence budbreak via a redox-mediated budbreak mechanism

There is increasing evidence of an important role for redox status in dormancy regulation, with the emerging physiological model for bud dormancy in fruit trees being that of a hormonally regulated process mediated by chill-induced stress and ROS production. ROS, particularly H_2_O_2_, are thought to be important cues in dormancy control (reviewed in Considine and Foyer, 2014 and Beauvieux et al., 2018) and peroxidase enzymes are crucial factors in this process. Endodormancy release and bud burst correlate with an increase in H_2_O_2_ and O_2_•− levels in grapevine buds (Pérez et al., 2008). In Japanese pear, chilling is necessary to elevate H_2_O_2_ levels in flower buds at the onset of endodormancy release (Kuroda et al., 2002) and peroxidase activity has been shown to be elevated during transition to ecodormancy (Takemura et al., 2015). Up-regulation of many peroxidase genes was observed in dormancy-released buds of peach (Leida et al., 2010) and Japanese apricot (*Prunus mume*; Zhuang et al., 2013). Plant peroxidases catalyze the reduction of H_2_O_2_ via a peroxidative cycle but can also generate H_2_O_2_ via a hydroxylic process, with both activities generating other ROS compounds, such as superoxide (O_2_^•−^; Passardi et al., 2005). They are therefore not only involved in regulating levels of H_2_O_2_ but are integral with regard to redox status. In order to determine how MdPRX10 contributes to redox-mediated budbreak it will be necessary to discover its intended ligand and, indeed, how redox signaling influences various stages in dormancy and budbreak. Recently, Sapkota et al. (2021) compared the levels of ROS and ROS-related compounds, O_2_^•−^ and NADPH oxidase, as well as H_2_O_2_, in the bud tissue of a late (Honeycrisp) and an early (Cripps Pink, also known as Pink Lady) budbreak cultivar. While all compounds demonstrated a distinctive peak in concentration in the early cultivar just prior to endodormancy release, which was absent in the late cultivar, budbreak itself appeared characterized by low levels of H_2_O_2_ and high levels of O_2_^•−^ and NADPH oxidase in both cultivars. A further transcriptomic study, conducted on the same contrasting cultivars, suggested that the delayed and lower levels of H_2_O_2_ in Honeycrisp compared to Cripps Pink were due to the transcriptional downregulation of genes encoding peroxidase and ascorbate oxidase enzymes (Sapkota et al., 2023). In sweet cherry (*Prunus avium* L.), Vimont et al. (2019) described how genes of two different peroxidases were specifically activated at endodormancy release and during ecodormancy, respectively, further indicating that these enzymes may perform specific tasks at precise moments during dormancy. In the current study, altered function of MdPRX10 in the late cultivars may be prolonging the presence of a certain ROS, or indeed preventing the accumulation of such a compound, that is required to progress a certain aspect of dormancy. The translation of these factors into the eventual time of budbreak must also be influenced by genetic background. For example, the late cultivar, X8390, demonstrated a much longer endodormancy than both the others, demonstrating that despite the large effect of this locus on budbreak, there are many other contributors along the path of dormancy. While these other loci were not identified in this study, they remain important to achieving a full picture of the genetic underpinnings of budbreak. Various types of environmental stresses lead to distinct ROS patterns. These patterns can be detected directly by redox-sensitive transcription factors and receptors to be incorporated into diverse signaling pathways (He et al., 2018). For example, H_2_O_2_ generated during plant shoot apical meristem development initiates the reversible protein phase separation of TERMINATING FLOWER (TMF), a transcription factor responsible for timing the flowering transition in tomato. Phase separation enables TMF to bind the promoter and repress the expression of the floral identity gene *ANANTHA* (*AN*) (Huang et al., 2021). In the case of late budbreak apple cultivars carrying the SChr09_680633 SNP T allele within *MdPRX10*, potential changes in H_2_O_2_ levels could affect the activity of unknown transcriptions factors that in turn, regulate the expression of dormancy-related genes. This could explain the lower and higher levels of *MdFT2* and *MdDAM4* expression, respectively, which correlate with the phenotype of the late cultivars. Further studies will be necessary to link MdPRX10 activity, ROS signaling and dormancy-related gene expression triggering budbreak in apple.

## 5 Conclusions

The LG9 QTL has long been associated with dormancy and budbreak in apple and in this study, we endeavored to identify its underlying genetic contributor(s) within the context of a diverse, dessert apple core collection. By enriching a large SNP dataset, using a novel capture sequencing pipeline, with polymorphisms present in dormancy and flowering-related genes as well as those within the QTL interval, we identified a peroxidase gene, *MdPRX10*, with a robust association to budbreak timing. Expression analysis indicated both a role in dormancy and a response to chilling, while the SChr09_680633 SNP was predicted to alter protein dimer conformation and potentially function. We propose *MdPRX10* plays a role in budbreak through alteration of cold perception, potentially via redox-mediated signaling and *CBF* gene regulation. These findings could provide further insight into our developing understanding of how ROS may influence important points during bud development and flowering and in understanding the effects of climate change on flowering time. This is crucial to being able to make targeted selections in breeding programs, potentially through the use of advanced breeding technologies and marker-assisted selection to develop cultivars better adapted to warmer temperatures.

## Data availability statement

The datasets presented in this study can be found in online repositories. The names of the repository/repositories and accession number(s) can be found below: https://www.ncbi.nlm.nih.gov/bioproject/PRJNA1023873, PRJNA1023873.

## Author contributions

AW: Conceptualization, Formal analysis, Investigation, Writing – original draft. BG: Conceptualization, Data curation, Formal analysis, Investigation, Methodology, Writing – review & editing. AS: Data curation, Formal analysis, Methodology, Software, Writing – review & editing. RR: Writing – review & editing, Methodology. HV: Writing – review & editing, Methodology. IF: Investigation, Writing – review & editing, Methodology. BH: Writing – review & editing, Methodology. CA: Investigation, Writing – review & editing, Methodology. VF: Investigation, Writing – review & editing. AC: Formal analysis, Investigation, Writing – review & editing. VS: Conceptualization, Methodology, Supervision, Writing – review & editing. GS: Data curation, Software, Writing – review & editing. JD: Data curation, Software, Writing – review & editing. SS: Writing – review & editing, Data curation, Software. EC: Conceptualization, Supervision, Writing – review & editing, Funding acquisition, Investigation. FA: Conceptualization, Funding acquisition, Investigation, Project administration, Supervision, Writing – review & editing.

## References

[B1] Albert T. J. Molla M. N. Muzny D. M. Nazareth L. Wheeler D. Song X.. (2007). Direct selection of human genomic loci by microarray hybridization. Nat. Methods 4, 903–905. doi: 10.1038/nmeth1111 17934467

[B2] Allard A. Bink M. C. A. M. Martinez S. Kelner J. J. Legave J. M. Di Guardo M.. (2016). Detecting QTLs and putative candidate genes involved in budbreak and flowering time in an apple multiparental population. J. Exp. Bot. 67, 2875–2888. doi: 10.1093/jxb/erw130 27034326 PMC4861029

[B3] Alseekh S. Kostova D. Bulut M. Fernie A. R. (2021). Genome-wide association studies: assessing trait characteristics in model and crop plants. Cell. Mol. Life Sci. 78, 5743–5754. doi: 10.1007/s00018-021-03868-w 34196733 PMC8316211

[B4] Andermann T. Torres Jiménez M. F. Matos-Maraví P. Batista R. Blanco-Pastor J. L. Gustafsson A. L. S.. (2020). A guide to carrying out a phylogenomic target sequence capture project. Front. Genet. 10. doi: 10.3389/fgene.2019.01407 PMC7047930 32153629

[B5] Anderson J. L. Richardson E. A. Kesner C. D. (1986). Validation of chill unit and flower bud phenology models for ’Montmorency’ sour cherry. Acta Hortic. 184, 71–78. doi: 10.17660/ActaHortic.1986.184.7

[B6] Atkinson C. J. Brennan R. M. Jones H. G. (2013). Declining chilling and its impact on temperate perennial crops. Environ. Exp. Bot. 91, 48–62. doi: 10.1016/j.envexpbot.2013.02.004

[B7] Baggiolini M. (1980). Stades reperes de la abricotier-stades reperes de la pecher. Stades reperes du ceresier-stades reperes du prunier. Guide pratique de defense des cultures (Paris: Acta editions).

[B8] Bates D. Mächler M. Bolker B. Walker S. (2015). Fitting linear mixed-effects models using lme4. J. Stat. Software 67, 1–48. doi: 10.18637/jss.v067.i01

[B9] Beauvieux R. Wenden B. Dirlewanger E. (2018). Bud dormancy in perennial fruit tree species: A pivotal role for oxidative cues. Front. Plant Sci. 9. doi: 10.3389/fpls.2018.00657 PMC5969045 29868101

[B10] Bennett J. P. (1949). Temperature and bud rest period: effect of temperature and exposure on the rest period of deciduous plant leaf buds investigated. Calif. Agric. 3, 9–12. doi: 10.3733/ca.v003n11p9

[B11] Bernardes A. Textor L. C. Santos J. C. Cuadrado N. H. Kostetsky E. Y. Roig M. G.. (2015). Crystal structure analysis of peroxidase from the palm tree *Chamaerops excelsa* . Biochimie 111, 58–69. doi: 10.1016/j.biochi.2015.01.014 25660651

[B12] Bianco L. Cestaro A. Linsmith G. Muranty H. Denancé C. Théron A.. (2016). Development and validation of the Axiom® Apple480K SNP genotyping array. Plant J. 86, 62–74. doi: 10.1111/tpj.13145 26919684

[B13] Bielenberg D. G. Wang Y. Li Z. Zhebentyayeva T. Fan S. Reighard G. L.. (2008). Sequencing and annotation of the *evergrowing* locus in peach [*Prunus persica* (L.) Batsch] reveals a cluster of six MADS-box transcription factors as candidate genes for regulation of terminal bud formation. Tree Genet. Genomes 4, 495–507. doi: 10.1007/s11295-007-0126-9

[B14] Bouché F. Lobet G. Tocquin P. Périlleux C. (2016). FLOR-ID: An interactive database of flowering-time gene networks in *Arabidopsis thaliana* . Nucleic Acids Res. 44, 1167–1171. doi: 10.1093/nar/gkv1054 PMC4702789 26476447

[B15] Broekema R. V. Bakker O. B. Jonkers I. H. (2020). A practical view of fine-mapping and gene prioritization in the post-genome-wide association era. Open Biol. 10, 190221. doi: 10.1098/rsob.190221 31937202 PMC7014684

[B16] Cannell M. G. R. Smith R. I. (1986). Climatic warming, spring budburst and frost damage on trees. J. Appl. Ecol. 23, 177–191. doi: 10.2307/2403090

[B17] Carvajal-Millán E. Gardea A. A. Rascón-Chu A. Orozco J. A. de León N. P. Márquez-Escalante J. A.. (2007). Respiratory response of apple buds treated with budbreaking agents. Thermochim Acta 457, 109–112. doi: 10.1016/j.tca.2007.03.004

[B18] Celton J. M. Martinez S. Jammes M. J. Bechti A. Salvi S. Legave J. M.. (2011). Deciphering the genetic determinism of bud phenology in apple progenies: A new insight into chilling and heat requirement effects on flowering dates and positional candidate genes. New Phytol. 192, 378–392. doi: 10.1111/nph.2011.192.issue-2 21770946

[B19] Cheverud J. M. (2001). A simple correction for multiple comparisons in interval mapping genome scans. Heredity 87, 52–58. doi: 10.1046/j.1365-2540.2001.00901.x 11678987

[B20] Chinnusamy V. Ohta M. Kanrar S. Lee B. Hong X. Agarwal M.. (2003). ICE1: A regulator of cold-induced transcriptome and freezing tolerance in Arabidopsis. Genes Dev. 17, 1043–1054. doi: 10.1101/gad.1077503 12672693 PMC196034

[B21] Chuine I. Bonhomme M. Legave J. M. García de Cortázar-Atauri I. Charrier G. Lacointe A.. (2016). Can phenological models predict tree phenology accurately in the future? The unrevealed hurdle of endodormancy break. Glob. Change Biol. 22, 3444–3460. doi: 10.1111/gcb.13383 27272707

[B22] Cingolani P. Platts A. Wang L. L. Coon M. Nguyen T. Wang L.. (2012). A program for annotating and predicting the effects of single nucleotide polymorphisms, SnpEff: SNPs in the genome of Drosophila melanogaster strain w1118; iso-2; iso-3. Fly (Austin) 6, 80–92. doi: 10.4161/fly.19695 22728672 PMC3679285

[B23] Conner P. J. Brown S. K. Weeden N. F. (1998). Molecular-marker analysis of quantitative traits for growth and development in juvenile apple trees. Theor. Appl. Genet. 96, 1027–1035. doi: 10.1007/s001220050835

[B24] Considine M. J. Foyer C. H. (2014). Redox regulation of plant development. Antioxid. Redox Signal. 21, 1305–1326. doi: 10.1089/ars.2013.5665 24180689 PMC4158970

[B25] Cornelissen S. Hefer C. A. Rees D. J. G. Burger J. T. (2020). Defining the QTL associated with chill requirement during endodormancy in *Malus* × *domestica* Borkh. Euphytica 216, 122. doi: 10.1007/s10681-020-02645-3

[B26] Cruz F. Julca I. Gómez-Garrido J. Loska D. Marcet-Houben M. Cano E.. (2016). Genome sequence of the olive tree, *Olea europaea* . Gigascience 5, 29. doi: 10.1186/s13742-016-0134-5 27346392 PMC4922053

[B27] Daccord N. Celton J. M. Linsmith G. Becker C. Choisne N. Schijlen E.. (2017). High-quality *de novo* assembly of the apple genome and methylome dynamics of early fruit development. Nat. Genet. 49, 1099–1106. doi: 10.1038/ng.3886 28581499

[B28] Denancé C. Muranty H. Durel C.-E. (2022). FruitBreedomics apple 275K SNP genotypic data. Portail Data INRAE, V1. Dataset data.inrae.fr. doi: 10.15454/F5XIVJ

[B29] Dufayard J. F. Bocs S. Guignon V. Larivière D. Louis A. Oubda N.. (2021). RapGreen, an interactive software and web package to explore and analyze phylogenetic trees. NAR Genom. Bioinform. 3. doi: 10.1093/nargab/lqab088 PMC8459725 34568824

[B30] Falavigna V. da S. Guitton B. Costes E. Andrés F. (2019). I want to (Bud) break free: The potential role of DAM and SVP-like genes in regulating dormancy cycle in temperate fruit trees. Front. Plant Sci. 9. doi: 10.3389/fpls.2018.01990 PMC6335348 30687377

[B31] Falavigna V. Severing E. Lai X. Estevan J. Farrera I. Hugouvieux V.. (2021). Unraveling the role of MADS transcription factor complexes in apple tree dormancy. New Phytol. 232, 2071–2088. doi: 10.1111/nph.17710 34480759 PMC9292984

[B32] Feng X. M. Zhao Q. Zhao L. L. Qiao Y. Xie X. Li H. F.. (2012). The cold-induced basic helix-loop-helix transcription factor gene MdCIbHLH1 encodes an ICE-like protein in apple. BMC Plant Biol. 12, 22. doi: 10.1186/1471-2229-12-22 22336381 PMC3352023

[B33] Flutre T. (2019). Timothee Flutre’s personal R code. Available online at: https://github.com/timflutre/rutilstimflutre (Accessed 1 May 2022).

[B34] Gabay G. Dahan Y. Izhaki Y. Isaacson T. Elkind Y. Ben-Ari G.. (2017). Identification of QTLs associated with spring vegetative budbreak time after dormancy release in pear (*Pyrus communis* L.). Plant Breed. 136, 749–758. doi: 10.1111/pbr.12499

[B35] Gao X. Becker L. C. Becker D. M. Starmer J. D. Province M. A. (2010). Avoiding the high bonferroni penalty in genome-wide association studies. Genet. Epidemiol. 34, 100–105. doi: 10.1002/gepi.20430 19434714 PMC2796708

[B36] Gao X. Starmer J. Martin E. R. (2008). A multiple testing correction method for genetic association studies using correlated single nucleotide polymorphisms. Genet. Epidemiol. 32, 361–369. doi: 10.1002/gepi.20310 18271029

[B37] Gnirke A. Melnikov A. Maguire J. Rogov P. LeProust E. M. Brockman W.. (2009). Solution hybrid selection with ultra-long oligonucleotides for massively parallel targeted sequencing. Nat. Biotechnol. 27, 182–189. doi: 10.1038/nbt.1523 19182786 PMC2663421

[B38] Hauagge R. Cummins J. N. (1991). Phenotypic variation of length of bud dormancy in apple cultivars and related *Malus* species. J. Am. Soc Hortic. Sci. 116, 100–106. doi: 10.21273/JASHS.116.1.100

[B39] He H. Van Breusegem F. Mhamdi A. (2018). Redox-dependent control of nuclear transcription in plants. J. Exp. Bot. 69, 3359–3372. doi: 10.1093/jxb/ery130 29659979

[B40] Huang X. Chen S. Li W. Tang L. Zhang Y. Yang N.. (2021). ROS regulated reversible protein phase separation synchronizes plant flowering. Nat. Chem. Biol. 17, 549–557. doi: 10.1038/s41589-021-00739-0 33633378

[B41] Jiménez S. Lawton-Rauh A. L. Reighard G. L. Abbott A. G. Bielenberg D. G. (2009). Phylogenetic analysis and molecular evolution of the dormancy associated MADS-box genes from peach. BMC Plant Biol. 9, 81. doi: 10.1186/1471-2229-9-81 19558704 PMC2713236

[B42] Kardailsky I. Shukla V. K. Hoon Ahn J. Dagenais N. Christensen S. K. Nguyen J. T.. (1999). Activation tagging of the floral inducer FT. Science 286, 1962–1965. doi: 10.1126/science.286.5446.1962 10583961

[B43] Kenis K. Keulemans J. (2004). QTL analysis of growth characteristics in apple. Acta Hortic. 663, 369–374. doi: 10.17660/ActaHortic.2004.663.63

[B44] Kircher M. Sawyer S. Meyer M. (2012). Double indexing overcomes inaccuracies in multiplex sequencing on the Illumina platform. Nucleic Acids Res. 40, 3. doi: 10.1093/nar/gkr771 PMC3245947 22021376

[B45] Kobayashi Y. Kaya H. Goto K. Iwabuchi M. Araki T. (1999). A pair of related genes with antagonistic roles in mediating flowering signals. Science 286, 1960–1062. doi: 10.1126/science.286.5446.1960 10583960

[B46] Korte A. Farlow A. (2013). The advantages and limitations of trait analysis with GWAS: a review. Plant Methods 9, 29. doi: 10.1186/1746-4811-9-29 23876160 PMC3750305

[B47] Kuroda H. Sugiura T. Ito D. (2002). Changes in hydrogen peroxide content in flower buds of Japanese Pear (*Pyrus pyrifolia* Nakai) in relation to breaking of endodormancy. J. Jpn. Soc Hortic. Sci. 71, 610–616. doi: 10.2503/jjshs.71.610

[B48] Kuroda H. Sugiura T. Sugiura H. (2005). Effect of hydrogen peroxide on Breaking endodormancy in flower buds of Japanese pear (*Pyrus pyrifolia* Nakai). J. Japan. Soc Hortic. Sci. 74, 255–257. doi: 10.2503/jjshs.74.255

[B49] Kuznetsova A. Brockhoff P. B. Christensen R. H. B. (2017). lmerTest package: tests in linear mixed effects models. J. Stat. Software 82, 1–26. doi: 10.18637/jss.v082.i13

[B50] Lang G. A. Early J. D. Martin G. C. Darnell R. L. (1987). Endodormancy, paradormancy, and ecodormancy: physiological terminology and classification for dormancy research. Hort Sci. 22, 371–377. doi: 10.21273/HORTSCI.22.5.701b

[B51] Lassois L. Denancé C. Ravon E. Guyader A. Guisnel R. Hibrand-Saint-Oyant L.. (2016). Genetic diversity, population structure, parentage analysis, and construction of core collections in the French apple germplasm based on SSR markers. Plant Mol. Biol. Rep. 34, 827–844. doi: 10.1007/s11105-015-0966-7

[B52] Lee S. Lee S. Yang K. Y. Kim Y. M. Park S. Y. Kim S. Y.. (2006). Overexpression of PRE1 and its homologous genes activates gibberellin-dependent responses in *Arabidopsis thaliana* . Plant Cell Physiol. 47, 591–600. doi: 10.1093/pcp/pcj026 16527868

[B53] Lee E. S. Park J. H. Wi S. D. Kang C. H. Chi Y. H. Chae H. B.. (2021). Redox-dependent structural switch and CBF activation confer freezing tolerance in plants. Nat. Plants 7, 914–922. doi: 10.1038/s41477-021-00944-8 34155371

[B54] Legave J. M. Farrera I. Almeras T. Calleja M. (2008). Selecting models of apple flowering time and understanding how global warming has had an impact on this trait. J. Hortic. Sci. Biotechnol. 83, 76–84. doi: 10.1080/14620316.2008.11512350

[B55] Leida C. Terol J. Martí G. Agustí M. Llácer G. Badenes M. L.. (2010). Identification of genes associated with bud dormancy release in *Prunus persica* by suppression subtractive hybridization. Tree Physiol. 30, 655–666. doi: 10.1093/treephys/tpq008 20231169

[B56] Lempe J. Peil A. Flachowsky H. (2022). Time-resolved analysis of candidate gene expression and ambient temperature during bud dormancy in apple. Front. Plant Sci. 12. doi: 10.3389/fpls.2021.803341 PMC8802299 35111181

[B57] Lenth R. (2022). emmeans: Estimated Marginal Means, aka Least-Squares Means. R package version 1.8.3. Available online at: https://CRAN.R-project.org/package=emmeans.

[B58] Li H. Durbin R. (2010). Fast and accurate long-read alignment with Burrows-Wheeler transform. Bioinform. 26, 589–595. doi: 10.1093/bioinformatics/btp698 PMC2828108 20080505

[B59] Liu J. Sherif S. M. (2019). Hormonal orchestration of bud dormancy cycle in deciduous woody perennials. Front. Plant Sci. 10. doi: 10.3389/fpls.2019.01136 PMC6759871 31620159

[B60] Livak K. J. Schmittgen T. D. (2001). Analysis of relative gene expression data using real-time quantitative PCR and the 2-ΔΔCT method. Methods 25, 402–408. doi: 10.1006/meth.2001.1262 11846609

[B61] Luedeling E. Caspersen L. Fernandez E. (2023). chillR: Statistical methods for phenology analysis in temperate fruit trees. R package version 0.75. Available online at: https://CRAN.R-project.org/package=chillR.

[B62] Lyons E. Freeling M. (2008). How to usefully compare homologous plant genes and chromosomes as DNA sequences. Plant J. 53, 661–673. doi: 10.1111/j.1365-313X.2007.03326.x 18269575

[B63] Malagi G. Sachet M. R. Citadin I. Herter F. G. Bonhomme M. Regnard J. L.. (2015). The comparison of dormancy dynamics in apple trees grown under temperate and mild winter climates imposes a renewal of classical approaches. Trees – Struct. 29, 1365–1380. doi: 10.1007/s00468-015-1214-3

[B64] Martin M. (2011). Cutadapt removes adapter sequences from high-throughput sequencing reads. EMBnet.journal 17, 10–12. doi: 10.14806/ej.17.1.200

[B65] McKenna A. Hanna M. Banks E. Sivachenko A. Cibulskis K. Kernytsky A.. (2010). The genome analysis toolkit: A MapReduce framework for analyzing next-generation DNA sequencing data. Genome Res. 20, 1297–1303. doi: 10.1101/gr.107524.110 20644199 PMC2928508

[B66] Meyer M. Kircher M. (2010). Illumina sequencing library preparation for highly multiplexed target capture and sequencing. Cold Spring Harb. Protoc. 2010. doi: 10.1101/pdb.prot5448 20516186

[B67] Mimida N. Saito T. Moriguchi T. Suzuki A. Komori S. Wada M. (2015). Expression of DORMANCY-ASSOCIATED MADS-BOX (DAM)-like genes in apple. Biol. Plant 59, 237–244. doi: 10.1007/s10535-015-0503-4

[B68] Miotto Y. E. Tessele C. Czermainski A. B. C. Porto D. D. Falavigna V. da S.. (2019). Spring is coming: Genetic analyses of the bud break date locus reveal candidate genes from the cold perception pathway to dormancy release in apple (*Malus* × *Domestica* borkh.). Front. Plant Sci. 10. doi: 10.3389/fpls.2019.00033 PMC6423911 30930909

[B69] Moser M. Asquini E. Miolli G. V. Weigl K. Hanke M. V. Flachowsky H.. (2020). The MADS-Box gene MdDAM1 controls growth cessation and bud dormancy in apple. Front. Plant Sci. 11. doi: 10.3389/fpls.2020.01003 PMC7358357 32733512

[B70] Nishiyama S. Matsushita M. C. Yamane H. Honda C. Okada K. Tamada Y.. (2021). Functional and expressional analyses of apple FLC-like in relation to dormancy progress and flower bud development. Tree Physiol. 41, 562–570. doi: 10.1093/treephys/tpz111 31728534

[B71] Niu Q. Li J. Cai D. Qian M. Jia H. Bai S.. (2016). Dormancy-associated MADS-box genes and microRNAs jointly control dormancy transition in pear (*Pyrus pyrifolia* white pear group) flower bud. J. Exp. Bot. 67, 239–257. doi: 10.1093/jxb/erv454 26466664 PMC4682432

[B72] Ntladi S. M. Human J. P. Bester C. Vervalle J. Roodt-Wilding R. Tobutt K. R. (2018). Quantitative trait loci (QTL) mapping of blush skin and flowering time in a European pear (*Pyrus communis*) progeny of ‘Flamingo’ × ‘Abate Fetel.’. Tree Genet. Genomes 14, 70. doi: 10.1007/s11295-018-1280-y

[B73] Passardi F. Cosio C. Penel C. Dunand C. (2005). Peroxidases have more functions than a Swiss army knife. Plant Cell Rep. 24, 255–265. doi: 10.1007/s00299-005-0972-6 15856234

[B74] Pérez F. J. Vergara R. Rubio S. (2008). H2O2 is involved in the dormancy-breaking effect of hydrogen cyanamide in grapevine buds. Plant Growth Regul. 55, 149–155. doi: 10.1007/s10725-008-9269-4

[B75] Perini P. Pasquali G. Margis-Pinheiro M. de Oliviera P. R. D. Revers L. F. (2014). Reference genes for transcriptional analysis of flowering and fruit ripening stages in apple (*Malus* × *domestica* Borkh.). Mol. Breed. 34, 829–842. doi: 10.1007/s11032-014-0078-3

[B76] Petri J. L. Leite G. B. (2004). Consequences of insufficient winter chilling on apple tree bud-break. Acta Hortic. 662, 53–60. doi: 10.17660/ActaHortic.2004.662.4

[B77] Porto D. D. Bruneau M. Perini P. Anzanello R. Renou J. P. Dos Santos H. P.. (2015). Transcription profiling of the chilling requirement for bud break in apples: A putative role for FLC-like genes. J. Exp. Bot. 66, 2659–2672. doi: 10.1093/jxb/erv061 25750421

[B78] R Core Team. (2023). R: A language and environment for statistical computing (Vienna, Austria: R Foundation for statistical Computing). Available at: https://www.R-project.org/.

[B79] Risterucci A. M. Hippolyte I. Perrier X. Xia L. Caig V. Evers M.. (2009). Development and assessment of diversity arrays technology for high-throughput DNA analyses in musa. Theor. Appl. Genet. 119, 1093–1103. doi: 10.1007/s00122-009-1111-5 19693484

[B80] Rodriguez J. Sherman W. B. Scorza R. Wisniewski M. Okie W. R. (1994). “Evergreen” Peach, its inheritance and dormant behavior. J. Amer. Soc Hortic. Sci. 119, 789–792. doi: 10.21273/JASHS.119.4.789

[B81] Rozen S. Skaletsky H. (2000). Primer3 on the WWW for general users and for biologist programmers. Methods Mol. Biol. 132, 365–386. doi: 10.1385/1-59259-192-2:365 10547847

[B82] Ruijter J. M. Ramakers C. Hoogaars W. M. H. Karlen Y. Bakker O. van den hoff M. J. B.. (2009). Amplification efficiency: Linking baseline and bias in the analysis of quantitative PCR data. Nucleic Acids Res. 37, 45. doi: 10.1093/nar/gkp045 PMC2665230 19237396

[B83] Saito T. Bai S. Imai T. Ito A. Nakajima I. Moriguchi T. (2015). Histone modification and signalling cascade of the dormancy-associated MADS-box gene, PpMADS 13-1, in Japanese pear (*Pyrus pyrifolia*) during endodormancy. Plant Cell Environ. 38, 1157–1166. doi: 10.1111/pce.12469 25311427

[B84] Sapkota S. Liu J. Islam M. T. Sherif S. M. (2021). Changes in reactive oxygen species, antioxidants and carbohydrate metabolism in relation to dormancy transition and bud break in apple (*Malus* × *domestica* borkh) cultivars. Antioxidants 10, 1549. doi: 10.3390/antiox10101549 34679683 PMC8532908

[B85] Sapkota S. Salem M. Jahed K. R. Artlip T. S. Sherif S. M. (2023). From endodormancy to ecodormancy: the transcriptional landscape of apple floral buds. Front. Plant Sci. 14. doi: 10.3389/fpls.2023.1194244 PMC10375413 37521930

[B86] Segura V. Vilhjálmsson B. J. Platt A. Korte A. Seren Ü. Long Q.. (2012). An efficient multi-locus mixed-model approach for genome-wide association studies in structured populations. Nat. Genet. 44, 825–830. doi: 10.1038/ng.2314 22706313 PMC3386481

[B87] Tabuenca M. C. (1964). Chilling requirements of apricot, peach and pear varieties. Aula Dei 7, 113–132.

[B88] Takemura Y. Kuroki K. Jiang M. Matsumoto K. Tamura F. (2015). Identification of the expressed protein and the impact of change in ascorbate peroxidase activity related to endodormancy breaking in *Pyrus pyrifolia* . Plant Physiol. Biochem. 86, 121–129. doi: 10.1016/j.plaphy.2014.11.016 25438144

[B89] Takeuchi T. Matsushita M. C. Nishiyama S. Yamane H. Banno K. Tao R. (2018). RNA-sequencing analysis identifies genes associated with chilling-mediated endodormancy release in apple. J. Amer. Soc Hortic. Sci. 143, 194–206. doi: 10.21273/JASHS04345-18

[B90] Trainin T. Zohar M. Shimoni-Shor E. Doron-Faigenboim A. Bar-Ya’akov I. Hatib K.. (2016). A Unique haplotype found in apple accessions exhibiting early bud-break could serve as a marker for breeding apples with low chilling requirements. Mol. Breed. 36, 158. doi: 10.1007/s11032-016-0575-7

[B91] Urrestarazu J. Muranty H. Denancé C. Leforestier D. Ravon E. Guyader A.. (2017). Genome-wide association mapping of flowering and ripening periods in apple. Front. Plant Sci. 8. doi: 10.3389/fpls.2017.01923 PMC5686452 29176988

[B92] van Dyk M. M. Soeker M. K. Labuschagne I. F. Rees D. J. G. (2010). Identification of a major QTL for time of initial vegetative budbreak in apple (*Malus* x *domestica* Borkh.). Tree Genet. Genomes 6, 489–502. doi: 10.1007/s11295-009-0266-1

[B93] VanRaden P. M. (2008). Efficient methods to compute genomic predictions. J. Dairy Sci. 91, 4414–4423. doi: 10.3168/jds.2007-0980 18946147

[B94] Vimont N. Fouché M. Campoy J. A. Tong M. Arkoun M. Yvin J. C.. (2019). From bud formation to flowering: Transcriptomic state defines the cherry developmental phases of sweet cherry bud dormancy. BMC Genomics 20, 974. doi: 10.1186/s12864-019-6348-z 31830909 PMC6909552

[B95] Wang M. Le Moigne M. A. Bertheloot J. Crespel L. Perez-Garcia M. D. Ogé L.. (2019). BRANCHED1: A key hub of shoot branching. Front. Plant Sci. 10. doi: 10.3389/fpls.2019.00076 PMC6379311 30809235

[B96] Wi S. D. Lee E. S. Park J. H. Chae H. B. Paeng S. K. Bae S.. (2022). Redox-mediated structural and functional switching of C-repeat binding factors enhances plant cold tolerance. New Phytol. 233, 1067–1073. doi: 10.1111/nph.17745 34537981

[B97] Wisniewski M. Nassuth A. Teulières C. Marque C. Rowland J. Cao P. B.. (2014). Genomics of cold hardiness in woody plants. CRC Crit. Rev. Plant Sci. 33, 92–124. doi: 10.1080/07352689.2014.870408

[B98] Wisniewski M. Norelli J. Artlip T. (2015). Overexpression of a peach CBF gene in apple: A model for understanding the integration of growth, dormancy, and cold hardiness in woody plants. Front. Plant Sci. 6. doi: 10.3389/fpls.2015.00085 PMC4343015 25774159

[B99] Wisniewski M. Norelli J. Bassett C. Artlip T. Macarisin D. (2011). Ectopic expression of a novel peach (*Prunus persica*) CBF transcription factor in apple (*Malus* × *domestica*) results in short-day induced dormancy and increased cold hardiness. Planta 233, 971–983. doi: 10.1007/s00425-011-1358-3 21274560

[B100] Wu R. Cooney J. Tomes S. Rebstock R. Karunairetnam S. Allan A. C.. (2021). RNAi-mediated repression of dormancy-related genes results in evergrowing apple trees. Tree Physiol. 41, 1510–1523. doi: 10.1093/treephys/tpab007 33564851

[B101] Wu R. Tomes S. Karunairetnam S. Tustin S. D. Hellens R. P. Allan A. C.. (2017). SVP-Like MADS box genes control dormancy and budbreak in apple. Front. Plant Sci. 8. doi: 10.3389/fpls.2017.00477 PMC5378812 28421103

[B102] Zhang L. Y. Bai M. Y. Wu J. Zhu J. Y. Wang H. Zhang Z.. (2009). Antagonistic HLH/bHLH transcription factors mediate brassinosteroid regulation of cell elongation and plant development in rice and Arabidopsis. Plant Cell 21, 3767–3780. doi: 10.1105/tpc.109.070441 20009022 PMC2814508

[B103] Zhou X. Stephens M. (2012). Genome-wide efficient mixed-model analysis for association studies. Nat. Genet. 44, 821–824. doi: 10.1038/ng.2310 22706312 PMC3386377

[B104] Zhuang W. Gao Z. Wang L. Zhong W. Ni Z. Zhang Z. (2013). Comparative proteomic and transcriptomic approaches to address the active role of GA4 in Japanese apricot flower bud dormancy release. J. Exp. Bot. 64, 4953–4966. doi: 10.1093/jxb/ert284 24014872 PMC3830480

