# Supplementary material for: Target enrichment sequencing coupled with GWAS identifies MdPRX10 as a candidate gene in the control of budbreak in apple
Source: Front Plant Sci. 2024 Feb 21;15:1352757. doi: 10.3389/fpls.2024.1352757 (PMC10918860; doi:10.3389/fpls.2024.1352757)
Supplement: Supplementary file 1 [file DataSheet_1.zip › Supplementary Material/Supplementary File 1.PDF]

Supplementary File 1. Alignment of Orthogroup Prx31 from Redoxibase Class III (https://peroxibase.toulouse.inra.fr/orthogroups/view_orthogroup/Prx031) containing 91 protein sequences from 58 species. The red square denotes the amino acid residues aligned to the Phe156 of MdPRX10.

```
                                cov    pid   1 [       .         .         .         .         :         .         .         . 80
 1 17735|MdPrx10|          100.0% 100.0%   --------------------------------------------------------------------------------
 2 16551|AtrPrx16|          94.4%  61.4%   --------------------------------------------------------------------------------
 3 16793|EferPrx37|         97.7%  58.0%   --------------------------------------------------------------------------------
 4 2273|AmajPrx06|          63.5%  31.0%   --------------------------------------------------------------------------------
 5 2275|AmajPrx08|          68.1%  31.8%   --------------------------------------------------------------------------------
 6 14835|AhalPrx54|         88.0%  37.0%   --------------------------------------------------------------------------------
 7 7489|AlyPrx55|           94.7%  58.5%   --------------------------------------------------------------------------------
 8 221|AtPrx55|             94.7%  59.1%   --------------------------------------------------------------------------------
 9 15900|BvPrx47|           94.2%  67.3%   --------------------------------------------------------------------------------
10 14968|BstrPrx41|         96.2%  60.2%   --------------------------------------------------------------------------------
11 1293|BnPrx55-1_other|    91.2%  61.3%   --------------------------------------------------------------------------------
12 6939|BnPrx55-2_other|    95.3%  61.4%   --------------------------------------------------------------------------------
13 3019|BoPrx07-1Ca_other|  51.2%  33.3%   --------------------------------------------------------------------------------
14 3018|BoPrx07-1Cb_other|  61.1%  36.4%   --------------------------------------------------------------------------------
15 3017|BoPrx07-2Ca_other|  88.0%  36.2%   --------------------------------------------------------------------------------
16 4252|BrPrx55-1Aa_other|  93.3%  65.2%   --------------------------------------------------------------------------------
17 11240|BrPrx55-1Ab_other| 95.3%  59.7%   --------------------------------------------------------------------------------
18 14884|CagraPrx38|        96.2%  60.1%   --------------------------------------------------------------------------------
19 15070|CrubPrx70|         96.5%  61.5%   --------------------------------------------------------------------------------
20 8748|CpapPrx14|          93.9%  67.7%   --------------------------------------------------------------------------------
21 11285|CroPrx27|          95.6%  65.3%   --------------------------------------------------------------------------------
22 8661|CclPrx100|          86.8%  37.9%   --------------------------------------------------------------------------------
23 8700|CclPrx43|           93.9%  69.8%   --------------------------------------------------------------------------------
24 8908|CsPrx20|            94.7%  68.9%   --------------------------------------------------------------------------------
25 8814|CsaPrx24|           94.4%  66.1%   --------------------------------------------------------------------------------
26 8815|CsaPrx25|           95.3%  67.6%   --------------------------------------------------------------------------------
27 8816|CsaPrx27|           96.2%  66.7%   --------------------------------------------------------------------------------
28 10296|EcamPrx156|        94.2%  67.3%   --------------------------------------------------------------------------------
29 10428|EcamPrx[P]183|     57.3%  26.3%   --------------------------------------------------------------------------------
30 10429|EcamPrx[P]99|      45.6%  35.4%   --------------------------------------------------------------------------------
31 10975|EglPrx156|         94.2%  66.7%   --------------------------------------------------------------------------------
32 10920|EglPrx[P]99|       39.5%  24.6%   --------------------------------------------------------------------------------
33 8120|EgrPrx156|          94.4%  67.1%   --------------------------------------------------------------------------------
34 8050|EgrPrx[P]99|        70.5%  35.4%   --------------------------------------------------------------------------------
35 11464|EguPrx156|         94.2%  67.0%   --------------------------------------------------------------------------------
36 11606|EguPrx[P]99|       70.5%  33.2%   --------------------------------------------------------------------------------
37 9008|GmPrx272|           95.3%  67.2%   --------------------------------------------------------------------------------
38 585|GmPrx56|             95.3%  67.6%   --------------------------------------------------------------------------------
39 9046|GmPrx[P]269|        90.9%  35.2%   --------------------------------------------------------------------------------
40 1478|HaPrx08|            94.2%  68.7%   --------------------------------------------------------------------------------
41 17498|IaquPrx45|         95.3%  70.2%   --------------------------------------------------------------------------------
42 17432|InPrx74|           94.2%  67.2%   --------------------------------------------------------------------------------
43 17614|InPrx[P]123|       92.1%  35.7%   --------------------------------------------------------------------------------
44 17640|KfedPrx47|         93.6%  61.5%   --------------------------------------------------------------------------------
45 17648|KfedPrx55|         93.9%  57.0%   --------------------------------------------------------------------------------
46 3911|LperPrx49|          79.2%  33.8%   --------------------------------------------------------------------------------
47 3912|LsPrx49|            71.9%  33.6%   --------------------------------------------------------------------------------
48 3487|LsaPrx49|           61.1%  30.7%   --------------------------------------------------------------------------------
49 3909|LsePrx49|           82.2%  33.0%   --------------------------------------------------------------------------------
50 3910|LvPrx49|            75.4%  33.9%   --------------------------------------------------------------------------------
51 12117|LuPrx176|          93.9%  59.7%   --------------------------------------------------------------------------------
52 12127|LuPrx191|          93.9%  59.6%   --------------------------------------------------------------------------------
53 12179|LuPrx72|           96.5%  57.5%   --------------------------------------------------------------------------------
54 636|LePrx38|             94.2%  70.7%   --------------------------------------------------------------------------------
55 9122|MePrx64|            94.2%  70.1%   --------------------------------------------------------------------------------
56 365|MtPrx41|             93.0%  67.0%   --------------------------------------------------------------------------------
57 9255|MguPrx98|           93.0%  65.0%   --------------------------------------------------------------------------------
58 18385|NoffPrx55-1A|      95.6%  60.3%   --------------------------------------------------------------------------------
59 18448|NoffPrx55-1B|      95.3%  60.6%   --------------------------------------------------------------------------------
60 17107|NnPrx59|           94.7%  70.4%   --------------------------------------------------------------------------------
61 8252|NtPrx44-1A|         94.2%  72.2%   --------------------------------------------------------------------------------
62 17698|NtPrx44-1B|        98.2%  66.9%   --------------------------------------------------------------------------------
63 3099|PtPrx21|            93.9%  67.3%   --------------------------------------------------------------------------------
64 3177|PtPrx82|            93.3%  67.3%   --------------------------------------------------------------------------------
65 3139|PtPrx[P]62|         72.8%  35.1%   --------------------------------------------------------------------------------
66 9283|PpePrx42|           95.0%  78.7%   --------------------------------------------------------------------------------
67 9398|RcPrx76|            95.3%  67.9%   --------------------------------------------------------------------------------
68 1942|StPrx39|            67.8%  31.1%   --------------------------------------------------------------------------------
69 15632|ThasPrx72|         96.8%  60.9%   --------------------------------------------------------------------------------
70 9772|TsPrx55|            96.2%  60.9%   --------------------------------------------------------------------------------
71 6634|VvPrx65|            93.6%  71.7%   --------------------------------------------------------------------------------
72 7877|BdiPrx111|          94.4%  53.0%   --------------------------------------------------------------------------------
73 18698|CescPrx56|         92.7%  62.1%   --------------------------------------------------------------------------------
74 18722|CescPrx80|         93.0%  61.8%   --------------------------------------------------------------------------------
75 18847|LminPrx49|         92.7%  64.5%   --------------------------------------------------------------------------------
76 18869|LminPrx71|         92.7%  64.8%   --------------------------------------------------------------------------------
77 1129|OsPrx119|           95.6%  52.9%   --------------------------------------------------------------------------------
78 1133|OsPrx123|           93.3%  54.5%   --------------------------------------------------------------------------------
79 9468|SiPrx123|           93.3%  54.9%   --------------------------------------------------------------------------------
80 9557|SiPrx64|            94.2%  50.0%   --------------------------------------------------------------------------------
81 970|SbPrx104|            94.4%  48.1%   --------------------------------------------------------------------------------
82 2756|SbPrx32|            94.4%  54.7%   --------------------------------------------------------------------------------
83 13194|SpolPrx07|         93.0%  61.9%   --------------------------------------------------------------------------------
84 17959|TaPrx109-1A|       93.9%  52.4%   --------------------------------------------------------------------------------
85 18096|TaPrx109-1B|       93.9%  52.1%   --------------------------------------------------------------------------------
86 18218|TaPrx109-1D|       93.9%  51.8%   --------------------------------------------------------------------------------
87 17919|TaPrx13-1A|        93.0%  55.0%   --------------------------------------------------------------------------------
88 452|TaPrx13-1B|          93.0%  55.4%   --------------------------------------------------------------------------------
89 17920|TaPrx13-1D|        98.0%  40.9%   MVHVTNGHFLCGNNIFQQIVLQIIQLPASIILSSIIICIIVRDQDEKERGKAVYIGRPIFAVPWPASHCQSLVAGVTSEA
90 725|ZmPrx29|             93.6%  54.1%   --------------------------------------------------------------------------------
91 790|ZmPrx80|             94.4%  51.5%   --------------------------------------------------------------------------------
   consensus/100%                          ................................................................................
   consensus/90%                           ................................................................................
   consensus/80%                           ................................................................................
   consensus/70%                           ................................................................................

                                cov    pid  81          .         1         .         .         .         .         :         . 160
 1 17735|MdPrx10|          100.0% 100.0%   -----------------------MEITRRERE---REREKKMKLWRR--GLLL-----VL--VMMFSQ------------
 2 16551|AtrPrx16|          94.4%  61.4%   -----------------------------------------MKVWRAGVFL-----------GILVIV------------
 3 16793|EferPrx37|         97.7%  58.0%   -------------------MTCGAGYIEGKRR-ERESIRSKMEDARV-----------YVF--LLVMM------------
 4 2273|AmajPrx06|          63.5%  31.0%   ---------------------------------------------MKSYGVL-------VMMMMMTVAVQ-----G----
 5 2275|AmajPrx08|          68.1%  31.8%   -----------------------ISYQSGSFI---*FVEWQWENFKIGFSLW-------V--FAFIATINAAGPLKLPVA
 6 14835|AhalPrx54|         88.0%  37.0%   --------------------------------------------------------------------------------
 7 7489|AlyPrx55|           94.7%  58.5%   ---------------------------MDTRRDDATKP---MTMW----F---------SGMLLFSMV------------
 8 221|AtPrx55|             94.7%  59.1%   ---------------------------MDIRSDDAKKP---MMMW----F---------LGMLLFSMV------------
 9 15900|BvPrx47|           94.2%  67.3%   -----------------------------------------MEVWKRLCS---------ILVIIVIMV------------
10 14968|BstrPrx41|         96.2%  60.2%   ---------------------MRKSIKMDTHSDDSTKP---MMVW----F---------LGMLLFYMV------------
11 1293|BnPrx55-1_other|    91.2%  61.3%   -----------------------------MCTASNSRK--HMMML----L---------LVGMLFFCM------------
12 6939|BnPrx55-2_other|    95.3%  61.4%   -----------------------------MYTGSDSRK--PMMLS----F---------LVGMLFFCM------------
```

```
13 3019|BoPrx07-1Ca_other|     51.2%   33.3%   --------------------------------------------------------------------------------
14 3018|BoPrx07-1Cb_other|     61.1%   36.4%   --------------------------------------------------------------------------------
15 3017|BoPrx07-2Ca_other|     88.0%   36.2%   --------------------------------------------------------------------------------
16 4252|BrPrx55-1Aa_other|     93.3%   65.2%   -----------------------------------------MMLW----F---------LVGMLFFCM------------
17 11240|BrPrx55-1Ab_other|    95.3%   59.7%   -----------------------------MDTSSDSKR--HMQTC----F---------FLGMLFFCM------------
18 14884|CagraPrx38|           96.2%   60.1%   ---------------------------MYSRGDDSTKPMM-MMMW----F---------LGMLLFYMV------------
19 15070|CrubPrx70|            96.5%   61.5%   ---------------------------MYSRGDDSTKPMMMMMMW----F---------LGMLLFYMV------------
20 8748|CpapPrx14|             93.9%   67.7%   ----------------------------------------MVEKWKRG-----------LILIVVCVI------------
21 11285|CroPrx27|             95.6%   65.3%   ------------------------------------MKVMVIRSCSS-WL---------LLLMVAMVV------------
22 8661|CclPrx100|             86.8%   37.9%   --------------------------------------------------------------------------------
23 8700|CclPrx43|              93.9%   69.8%   -----------------------------------------MGVMREMMVFL-------FT---ILLI------------
24 8908|CsPrx20|               94.7%   68.9%   ---------------------------------------MEMGVMREMMVFL-------FT---ILLI------------
25 8814|CsaPrx24|              94.4%   66.1%   --------------------------------------------MAGMAMRVA-----VLSLALLCML------------
26 8815|CsaPrx25|              95.3%   67.6%   ---------------------------MDEFG---GGRR----RRRMRWFL--------VFIILMGIF------------
27 8816|CsaPrx27|              96.2%   66.7%   --------------------------------------MGLRFLWKGLVLAVV-----VAW--AATGT------------
28 10296|EcamPrx156|          94.2%   67.3%   -----------------------------------------MARKLGLWL---------VVVFAVAAT------------
29 10428|EcamPrx[P]183|       57.3%   26.3%   -----------------------------------------MEVLASSVLFL-------VALHLVSAAVQAGGLLG----
30 10429|EcamPrx[P]99|        45.6%   35.4%   --------------------------------------------------------------------------------
31 10975|EglPrx156|           94.2%   66.7%   -----------------------------------------MARKLGLWL---------VVVFAVAAT------------
32 10920|EglPrx[P]99|         39.5%   24.6%   --------------------------------------------------------------------------------
33 8120|EgrPrx156|            94.4%   67.1%   -----------------------------------------MARKLGLWL---------AVVFAVAAT------------
34 8050|EgrPrx[P]99|          70.5%   35.4%   --------------------------------------------------------------------------------
35 11464|EguPrx156|           94.2%   67.0%   -----------------------------------------MARKLGLWL---------VVVFAVAAT------------
36 11606|EguPrx[P]99|         70.5%   33.2%   --------------------------------------------------------------------------------
37 9008|GmPrx272|             95.3%   67.2%   ----------------------------------------MEKSMRM----VL-----MA-LLAFTML------------
38 585|GmPrx56|               95.3%   67.6%   ----------------------------------------MEKIMRT----VL-----MALLMAFTML------------
39 9046|GmPrx[P]269|          90.9%   35.2%   -----------------------------------------------GSTTL-------PLSIIYGLSTQGNAP---KKS
40 1478|HaPrx08|              94.2%   68.7%   -----------------------------------------MAD---TPQRTI-----IYWLFIVAVL------------
41 17498|IaquPrx45|           95.3%   70.2%   ---------------------------------------MEMGRWKGGLHFF-------FIMIMITLI------------
42 17432|InPrx74|             94.2%   67.2%   ---------------------------------------MGKGRWKGGLRFF-------IIIIMITLI------------
43 17614|InPrx[P]123|         92.1%   35.7%   -----------------------------------------MSKATAAFTVV-------VAGLVFAA-------------
44 17640|KfedPrx47|           93.6%   61.5%   -----------------------------------------MAARTPFALVAF-----VTL--VVMGL------------
45 17648|KfedPrx55|           93.9%   57.0%   -----------------------------------------MAATSPFLFTAF-----MTL--VMLGL------------
46 3911|LperPrx49|            79.2%   33.8%   -----------------------------------------MKTGATILSFL-------SIVFFLSA-SASKLPLLLHPL
47 3912|LsPrx49|              71.9%   33.6%   -----------------------------------------MKTGATVFSFL-------AIMFFLSA-SASNLPSLLHPL
48 3487|LsaPrx49|             61.1%   30.7%   -----------------------------------------MNVSSFIITIL-------LILFSVSC-LADD--YSSTSL
49 3909|LsePrx49|             82.2%   33.0%   -----------------------------------------MKTGATVLSFL-------AIVFFLSA-SASNLPSLLHPL
50 3910|LvPrx49|              75.4%   33.9%   -----------------------------------------MKTSATVLSFL-------AIVFFLSA-SASNLPSLLHPL
51 12117|LuPrx176|            93.9%   59.7%   ---------------------------MGYFSS---ST--------------A-----AMIMIIVAIT------------
52 12127|LuPrx191|            93.9%   59.6%   ---------------------------MGYFSS---ST-------------SA-----AMIMIIVATT------------
53 12179|LuPrx72|             96.5%   57.5%   -----------------------MGSSRGCCSNGTTIT-------------AA-----MMIMVVVSIT------------
54 636|LePrx38|               94.2%   70.7%   -----------------------------------------MERLRGLYV----------LILAIILI------------
55 9122|MePrx64|              94.2%   70.1%   -----------------------------------------MEARRGLFLLMI-----------LMMI------------
56 365|MtPrx41|               93.0%   67.0%   -----------------------------------------MEKIRI----VF-----MAMVMAFTI-------------
57 9255|MguPrx98|             93.0%   65.0%   ---------------------------------------MEKNCKSTL------------WVLTILIL------------
58 18385|NoffPrx55-1A|        95.6%   60.3%   ---------------------------MDTRCDDSTQH---IMMW----F---------LGMMLFSMV------------
59 18448|NoffPrx55-1B|        95.3%   60.6%   ---------------------------MDTRSDDSTKT---IMVW----F---------LGMLFFSMV------------
60 17107|NnPrx59|             94.7%   70.4%   -----------------------------------------MEEV----IRLY-----VTLLVVLMGL------------
61 8252|NtPrx44-1A|           94.2%   72.2%   -----------------------------------------MERRRGLYV----------LIVAVIII------------
62 17698|NtPrx44-1B|          98.2%   66.9%   --MHKFVLLLQ-------PLDGREGKVKEKLK---KGQIFKMERRRGLYL----------LIVATLSI------------
63 3099|PtPrx21|              93.9%   67.3%   -----------------------------------------MERGRCMLL----------LMMVLIMD------------
64 3177|PtPrx82|              93.3%   67.3%   -----------------------------------------MERG--YML----------LLVVLIIA------------
65 3139|PtPrx[P]62|           72.8%   35.1%   --------------------------------------------------------------------------------
66 9283|PpePrx42|             95.0%   78.7%   -----------------------------------------MEACKGLLLLMV-----VL--VLMVMQ------------
67 9398|RcPrx76|              95.3%   67.9%   -----------------------------------------MEARRDLLMMKM-----IMIMIIVSSM------------
68 1942|StPrx39|              67.8%   31.1%   ---------------------------------------------MKSFVFL-------TAALFLVA------AVS----
69 15632|ThasPrx72|           96.8%   60.9%   -------------------------------M---PQKEEEMRKW----VVVL-----AMAVAMAMVM------------
70 9772|TsPrx55|              96.2%   60.9%   ---------------------------MDTRDNDSTKH--TMMMW----F---------YLGMLFFCM------------
71 6634|VvPrx65|              93.6%   71.7%   -----------------------------------------MQAWRRL------------CLVMVLLM------------
72 7877|BdiPrx111|           94.4%   53.0%   -----------------------------------------MEKRRRIS-LRA--VVAAMALMLSLGE------------
73 18698|CescPrx56|          92.7%   62.1%   -----------------------------------------MGYWRG---LCV-----VVL--L-MVV------------
74 18722|CescPrx80|          93.0%   61.8%   -----------------------------------------MEVGRG---LCV-----VVV--LLVTA------------
75 18847|LminPrx49|          92.7%   64.5%   -----------------------------------------MGLSKA---VSF-----VLV--F-LAA------------
76 18869|LminPrx71|          92.7%   64.8%   -----------------------------------------MGLSKA---VSF-----VLV--F-LAA------------
77 1129|OsPrx119|            95.6%   52.9%   -----------------------------------MFSSSAMERRRRSPCAAV--VMAAVAML---AV------------
78 1133|OsPrx123|            93.3%   54.5%   --------------------------------------------MERRGSLYGV---AAAAAVVVVVA------------
79 9468|SiPrx123|            93.3%   54.9%   -----------------------------------------MERWRKRGSLLAV---A------AVAL------------
80 9557|SiPrx64|             94.2%   50.0%   -----------------------------------------MERRRRS------VPLLVAAVFLAVAA------------
81 970|SbPrx104|             94.4%   48.1%   -----------------------------------------MEKRRRG--GVSSLM-VLAAALLTVAA------------
82 2756|SbPrx32|             94.4%   54.7%   -----------------------------------------ME-KRRRRHLLLA---AAAVV--LVAV------------
83 13194|SpolPrx07|          93.0%   61.9%   -----------------------------------------MESWRR---LSS-----LAV--LLLMA------------
84 17959|TaPrx109-1A|        93.9%   52.4%   -----------------------------------------MEKRRRSVCAVA--AAALVAAMLSSLT------------
85 18096|TaPrx109-1B|        93.9%   52.1%   -----------------------------------------MEKRRRSVCAVA--AVALVAAMLSSLT------------
86 18218|TaPrx109-1D|        93.9%   51.8%   -----------------------------------------MEKRRRSVCAVA--AVALVAAMLSSLG------------
87 17919|TaPrx13-1A|         93.0%   55.0%   -----------------------------------------MEQRRRGGCFCLL---AVA-----AAV------------
88 452|TaPrx13-1B|           93.0%   55.4%   -----------------------------------------MEQRRRGSCFCLL---AVT-----AVV------------
89 17920|TaPrx13-1D|         98.0%   40.9%   AAACSYSCLLPTGNQNSIPTRTRTRTRKHRTSERLSSRVRAMERRRRGGCFCLL---AVA-----VVV------------
90 725|ZmPrx29|              93.6%   54.1%   -----------------------------------------MEKWRRRRHLLLA---VAAVA--L--V------------
91 790|ZmPrx80|              94.4%   51.5%   -----------------------------------------MEKRRRGSGGVPSLAVAVLAALLAAAS------------
   consensus/100%                          ................................................................................
   consensus/90%                           ................................................................................
   consensus/80%                           ..................................................................h.............
   consensus/70%                           .........................................h..h..............hh..hhhhh............

                                cov     pid 161       .         .         .         2         .         .         .         . 240
 1 17735|MdPrx10|             100.0% 100.0%   -------GKESEGQLVENFYSASCP--NVESIVNQAVSRKR--DQTIITLAATLRLFLHDCFV--EGCDASIIIASPNGD
 2 16551|AtrPrx16|             94.4%   61.4%   -------FVASDAQLVENFYGLRCQ--TLEATVRQVVIMKL--NQTFVTVPSTLRLFFHDCFV--TGCDASVMISSPNRD
 3 16793|EferPrx37|            97.7%   58.0%   -------YGVANGQLVQNFYSSTCP--LVESIVRQAVVAKM--SQTFVTVPATLRLFFHDCFV--KGCDASIMIQSPKGD
 4 2273|AmajPrx06|             63.5%   31.0%   N------EYEYDDPLSSTYYHGSCP--QLEKIIYNKLHQWF--KKDSTLAPSLMKLHFHDCAI--RGCDGSILLDHDG--
 5 2275|AmajPrx08|             68.1%   31.8%   KVP----LVPASAFLSPFYYLKTCP--NLEGIIHQKVSDWI--KKDYTLAAAIIRLHFHDCAV--RGCDASILLDNRG--
 6 14835|AhalPrx54|            88.0%   37.0%   ------SWFPLDNLLSLNYYDKICP--DFEKIVDRKVREWT--KSDSSLGPALLRLIFHDCGV--TGCDASVLLDYEG--
 7 7489|AlyPrx55|              94.7%   58.5%   --------AESNAQLSENYYASTCP--NVELIVKQAVTTKF--QQTPTTAPATLRMFFHDCFV--EGCDASVFIASDNED
 8 221|AtPrx55|                94.7%   59.1%   --------AESNAQLSENYYASTCP--SVELIVKQAVTTKF--KQTVTTAPATLRMFFHDCFV--EGCDASVFIASENED
 9 15900|BvPrx47|              94.2%   67.3%   -------IEGGEAQLSENFYTTSCP--RVETIVKQAVAKKY--SQTFITAQATLRLFFHDCFV--EGCDASVMIFSPKGD
10 14968|BstrPrx41|            96.2%   60.2%   --------AESDAQLSENYYSSTCP--NVELMVQQAVTTKF--QQTPTTAPATLRIFFHDCFV--EGCDASVLIASENED
11 1293|BnPrx55-1_other|       91.2%   61.3%   -------VAESNAQLSENFYASTCP--NVELIVRQAVTTKY--QQTPITAPGTLRMFFHDCFV--SGCDASVLIASDNGD
12 6939|BnPrx55-2_other|       95.3%   61.4%   -------VTESNAQLSENFYASTCP--NVELIVRQAVTTKY--QQTPITAPGTLRMFFHDCFVDVYGCDASVLIASDNGD
13 3019|BoPrx07-1Ca_other|     51.2%   33.3%   --------------------------------------------------------------------------------
14 3018|BoPrx07-1Cb_other|     61.1%   36.4%   ------------------------------------------------------------------GCDASVLLDHKG--
15 3017|BoPrx07-2Ca_other|     88.0%   36.2%   ------VLVSFGHQLSLSYYDRICP--DFEKIVATKVREWT--KKDSSLGPALLRLLFHDCGV--TGCDASVLLDHEG--
16 4252|BrPrx55-1Aa_other|     93.3%   65.2%   -------VPESNAQLSENFYASTCP--NVELIVRQAVTTKY--QQTPITAPGTLRMFFHDCFV--GGCDASVLIASDNGD
17 11240|BrPrx55-1Ab_other|    95.3%   59.7%   -------VVESDAQLSENFYASTCP--NVELIVRQAVATKY--QQTTITAPATLRMFFHDCFV--GGCDASVLVTSDNGD
18 14884|CagraPrx38|           96.2%   60.1%   --------AESDAQLSENYYSSTCP--NVESIVQQAVATKF--QQTPTTAPATLRIFFHDCFV--EGCDGSVLIASENED
19 15070|CrubPrx70|            96.5%   61.5%   --------AESDAQLSENYYSSTCP--NVESIVQQAVATKF--QQTPTTAPATLRIFFHDCFV--EGCDGSVLIASENED
20 8748|CpapPrx14|             93.9%   67.7%   -------LQKGDGQLAENFYGVTCS--NVESIVKQAVLNKF--SQTFTTVPATLRLFFHDCFI--EGCDASILISSRNGD
21 11285|CroPrx27|             95.6%   65.3%   -------TGGGAQVLVENFYSSSCP--NVELIVQQSVASKMQATQTFTPIPATLRLFFHDCFV--EGCDGSVLIASPNGD
22 8661|CclPrx100|             86.8%   37.9%   -------------YLSLSHYHSTCP--DLEGIIHRQVQAWI--RKDYTFAASLIRLHFHDCVV--RGCDASILLSHKG--
23 8700|CclPrx43|              93.9%   69.8%   -------MQRGDGQLVENFYSSTCP--NVESIVNRVVSTKF--SQTFITVPATLRLFFHDCFI--VGCDASVLIQSPNGD
24 8908|CsPrx20|               94.7%   68.9%   -------MQRGDGQLVENFYSSTCP--NVESIVNRVVSTKF--SQTFITVPATLRLFFHDCFI--VGCDASVLIQSPNGD
25 8814|CsaPrx24|              94.4%   66.1%   -------IGVVHAQLSFNFYNSSCP--NVEQIVRQAVSLKI--NQTFVTIPATLRLFFHDCFV--QGCDASVMIASASGD
26 8815|CsaPrx25|              95.3%   67.6%   -------VEIGEGQLVKNFYKSTCP--NVEQIVTQAVRNKF--SQTIITISATLRLFFHDCFV--EGCDASVMIASPTGD
27 8816|CsaPrx27|              96.2%   66.7%   -------VKRGEAQLVENFYGSNCP--NLEQIVTQSVQTKF--AQTFVTIPATLRLFFHDCFV--EGCDASVLIASLNGD
28 10296|EcamPrx156|          94.2%   67.3%   -------AEVGRAQLTEGFYGQTCP--NVESIVKQAVTTKF--GQTFVTIPGTLRLFFHDCFI--EGCDASILISSPNGD
29 10428|EcamPrx[P]183|       57.3%   26.3%   T------VPIEEYGLSFQ*YQQSCP--QFEAFVGRKVKEWI--K-DYTLAPALMRLHFLDCVV--RGCNTSILLNHEG--
30 10429|EcamPrx[P]99|        45.6%   35.4%   ---------------------------------------------------ALMRLHFHDCVV--RGCDASILLNHEE--
31 10975|EglPrx156|           94.2%   66.7%   -------AEVGRAQLTEGFYGQTCP--NVESIVKQAVTTKF--GQTFVTIPGTLRLFFHDCFI--EGCDASILISSPNGD
32 10920|EglPrx[P]99|         39.5%   24.6%   --------------------------------------------------------------------------------
33 8120|EgrPrx156|            94.4%   67.1%   -------AEVGRAQLTEGFYGQTCP--NVESIVKQAVTTKF--GQTFITIPGTLRLFFHDCFI--EGCDASILISSPNGD
34 8050|EgrPrx[P]99|          70.5%   35.4%   ------------------------------------------------------------------------GVLPHN--
35 11464|EguPrx156|           94.2%   67.0%   -------AEVGRAQLTEGFYGQTCP--NVESIVKQAVTTKF--GQTFVTIPGTLRLFFHDCFI--EGCDASILISSPNGD
36 11606|EguPrx[P]99|         70.5%   33.2%   ------------------------------------------------------------------------GVxxxx--
37 9008|GmPrx272|             95.3%   67.2%   -------LSKGEGQLVENFYSLSCP--NVESIVKQAVTNKF--TQTITTGQATLRLFFHDCFV--EGCDASVIISSPNGD
38 585|GmPrx56|               95.3%   67.6%   -------ISKGEGQLVENFYSSSCP--NVESMVKQAVTNKF--TETITTGQATLRLFFHDCFV--EGCDASVIISSPNGD
```

```
39 9046|GmPrx[P]269|          90.9%  35.2%   FKP----LLPPEALLSIGHYHTTCP--DVEGIISQKVAAWV--KKDPTLAPAIIRLHFHDCAV--GGCDASILLNHPG--
40 1478|HaPrx08|              94.2%  68.7%   -------TGTAHGQLIENFYASTCP--MVEAIVKQTVNTKV--SQTFTTIPATLRLFFHDCFV--EGCDASVIIASPNGD
41 17498|IaquPrx45|           95.3%  70.2%   -------EWRAEGQLAENFYASTCP--NVEAIVQQVVTTKL--TQTFVTVPATLRLFFHDCFV--EGCDASVIIASPNGD
42 17432|InPrx74|             94.2%  67.2%   -------EWRAEGQLAENFYASTCP--NVELIVQQVVATKF--SQTFVTVPATLRLFFHDCFV--EGCDASVIIASPNGD
43 17614|InPrx[P]123|         92.1%  35.7%   ------LSGHCHGQLQAGFYNGKCGNNDVEGIVRGVVKSWY--KNDTTITAALLRMQFHDCFV--RGCDASILLDGSE--
44 17640|KfedPrx47|           93.6%  61.5%   -------TSPADAQLVENFYALSCP--LVEPIVRQAVAIKL--AQTFTTVPGTLRLFFHDCFV--EGCDASIMIASPNGD
45 17648|KfedPrx55|           93.9%  57.0%   -------CSPADARLEENFYGLRCP--LVETIVREAVAIKM--AQTFTTVPATLRLFFHDCFV--EGCDASIMIASPNGD
46 3911|LperPrx49|           79.2%  33.8%   KVPRLPDIPSFGDDLSYSLYQHSCS--GVEGIINRKVKEWV--KKDPTLAPSLMRLHFHDCAV--RGCDASILLDHKG--
47 3912|LsPrx49|             71.9%  33.6%   KVPRLPDISSFEDDLSYSLYQHSCS--GVEGIIYRKVKEWV--KKDPTLAPSLMRLHFHDCAV--RGCDASILLDHKG--
48 3487|LsaPrx49|            61.1%  30.7%   KVPCPSDLSALEDDLSYSLYHESCP--DLEGIIYRKVKEWV--AKDPTLAPSLLRLHYHDCVV--RGCDASILLDHEG--
49 3909|LsePrx49|            82.2%  33.0%   KVPRLPDISSFEDDLSYSLYQHSCR--GVEGIIYRKVKEWV--KKDPTLAPSLMRLHFHDCAV--RGCDASILLDHKG--
50 3910|LvPrx49|             75.4%  33.9%   KVPRLPDISSFEDDLSYSLYQHSCS--GVEGIIYRKVKEWV--KKDPTLAPSLMRLHFHDCAV--RGCDASILLDHKG--
51 12117|LuPrx176|           93.9%  59.7%   ---IAGGGGVSGQGLRENFYAGSCP--MVEAVVQQVVAAKA--GRQFPTLPATLRLFFHDCFV--TGCDASVMVASPNGD
52 12127|LuPrx191|           93.9%  59.6%   ---IAGGGGVSGQGLRENFYAGSCP--MVEPIVQQVVAAKA--GRQFPTLPATLRLFFHDCFV--TGCDASVMVASPNGD
53 12179|LuPrx72|            96.5%  57.5%   -------RTATGQQLTENYYIGSCP--NVEAVVQKVIAAKA--RKQFATIPGTLRLFFHDCFV--TGCDASVMVASPNGD
54 636|LePrx38|              94.2%  70.7%   -------LGGAEGELVENFYGFTCP--NVEFIVQQAVSLKF--SQTFVTIPATLRLFFHDCFV--EGCDASVMIASPNGD
55 9122|MePrx64|             94.2%  70.1%   -------TNNGEAQLTENFYSSSCP--AVEAIVKQAVSTKF--SQTFTTIPATLRLFFHDCFV--TGCDASIMVSSPNGD
56 365|MtPrx41|              93.0%  67.0%   -------FSSGECQLVENFYVSSCP--NVELVVAQAVTNKF--TQTITTGQATLRLFLHDCFV--EGCDASVMIASPNGD
57 9255|MguPrx98|            93.0%  65.0%   -------VRSGQGQLLLNFYGPRCP--NVEGIVRQAVLTKI--NQTFVTIPATVRLFFHDCFV--DGCDASVLIASPNND
58 18385|NoffPrx55-1A|       95.6%  60.3%   -------VVDSDAQLSENYYASTCP--NVELIVQQAVTTKF--QQTFTTAPATLRMFFHDCFV--EGCDASVLIVSDNED
59 18448|NoffPrx55-1B|       95.3%  60.6%   --------VESDAQLSENYYASTCP--NVELIVQQAVTTKF--QQTFTTAPATLRMFFHDCFV--EGCDASVLITSDNED
60 17107|NnPrx59|            94.7%  70.4%   -------AGRGEGQLAENFYAFTCP--NVEAIVRQTVSTKF--SQTFTTIPATLRLFFHDCFV--EGCDASVLIASPNGD
61 8252|NtPrx44-1A|          94.2%  72.2%   -------VSGAEGQLVENFYGFTCP--NVEFIVQQAVSTKF--SQTFVTIPATLRLFFHDCFV--EGCDASVLIASPNGD
62 17698|NtPrx44-1B|         98.2%  66.9%   -------VSGAEGQLVENFYGFTCP--NVEFMVQQAVSTKF--SQTFVTIPATLRLFFHDCFV--EGCDASVLIASPNGD
63 3099|PtPrx21|             93.9%  67.3%   -------IDRGEGQLVEDFYSLTCP--NVEALVKKAVSTKF--NQTFTTIPATLRLFFHDCFV--TGCDASTMVSSPNGD
64 3177|PtPrx82|             93.3%  67.3%   -------IGRGEGQLVENFYSSSCP--NVEGIVRQAVSTKF--RQTFTTIPATLRLFFHDCFV--TGCDASTMVSSPNGD
65 3139|PtPrx[P]62|          72.8%  35.1%   ------------------------------------------------------------------GCDGSILLNHDG--
66 9283|PpePrx42|            95.0%  78.7%   -------GRESEGQLAENFYSSTCP--NVEFIVKQAVSTKL--SQTPITIPATLRLFFHDCFV--EGCDASVMIASPNGD
67 9398|RcPrx76|             95.3%  67.9%   -------IGRGEGQLTENFYSSNCP--NVEAIVKQVVSTKF--RQTFTTIPATLRLFFHDCFV--TGCDASIMISSPNGG
68 1942|StPrx39|             67.8%  31.1%   A------SAYDEDDLPYDYYSKTCP--KLECIVHKKMEEWI--KKDYSLAPALMRLHFHDCFV--RGCDASILLDYEG--
69 15632|ThasPrx72|          96.8%  60.9%   -------ECGEAQQLREQFYGTRCP--NVEEIVKEAVSLKF--QQTFVTAPSTLRLFFHDCFV--QGCDASVLISSPNGD
70 9772|TsPrx55|             96.2%  60.9%   -------VAESDARLTENFYASTCP--NVELIVRQVVSTKI--EQTFTTAPATLRMFFHDCFV--GGCDASVMIASESGD
71 6634|VvPrx65|             93.6%  71.7%   -------VGQGEGQLAENFYSSSCP--NVEAIVKQEVSTKF--SQTFTTIPATLRLFFHDCFV--EGCDASVLISSPNGD
72 7877|BdiPrx111|           94.4%  53.0%   ---AASYDG-GSSGLSPGYYSKTCS--NLEKIVLREVTKKK--NETVVTIPAVLRLFFHDCLV--NGCDASVLIASHNND
73 18698|CescPrx56|          92.7%  62.1%   -------VGRGEAQLRQNFYRFTCP--NVENIVRRAVQAKL--QQTFVTVPATLRLFFHDCFV--EGCDASVLISSPNGD
74 18722|CescPrx80|          93.0%  61.8%   -------MGQGDAQLRQGFYGLTCP--LVEGIVRQTVATKI--SQTFTTVPATLRLFFHDCFV--EGCDASVLIASPNGD
75 18847|LminPrx49|          92.7%  64.5%   -------AEMVKSQLGPNFYSSSCP--NVESVVRQAVQTKV--SQTFVTIPATLRLFFHDCFV--EGCDASVVIFSPNGD
76 18869|LminPrx71|          92.7%  64.8%   -------AEMVKSQLRPNFYSSSCP--NVESVVRQAVQAKV--SQTFVTIPATLRLFFHDCFV--EGCDASVVIFSPNGD
77 1129|OsPrx119|            95.6%  52.9%   ---MPAFPG-VAADLSAGYYSSSCP--KLESIVRYEVSRKI--NETVVTIPAVLRLFFHDCLV--TGCDASALISSPNDD
78 1133|OsPrx123|            93.3%  54.5%   ---MAAAAGGEAARLSPEHYRSTCP--GVESVVRSVVARKV--KETFVTVPATLRLFFHDCFV--EGCDASVMIASRGND
79 9468|SiPrx123|            93.3%  54.9%   ---VAALLPSGEAKLSPDYYRSTCP--DVEAIVREAVAKKV--NETFVTVPATLRLFFHDCFV--EGCDASVIIASRDND
80 9557|SiPrx64|             94.2%  50.0%   ---MPAYPGEAATPLREDYYRYSCP--NLESIVRAEVARKI--NETVVTIPATLRQVFHDCMA--GGCDAAVLIASKKND
81 970|SbPrx104|             94.4%  48.1%   ---MPPPP-RVAAALSPDYYKDSCP--DLESIVRYEVTRKK--NETVVTIPATLRLAFHDCMV--GGCNAAVLIASKKND
82 2756|SbPrx32|             94.4%  54.7%   ---AAIPLPSAEAKLSPDYYRSTCP--DVEAIVRAVVAKKV--NETFVTVPATLRLFFHDCFV--EGCDASVMIASRDND
83 13194|SpolPrx07|          93.0%  61.9%   -------AGQVESQLRTDFYASTCP--DVESIVREEVRKKF--VQTFVTVPATLRLFFHDCFV--EGCDASVIIASPNGD
84 17959|TaPrx109-1A|        93.9%  52.4%   ---GAAYG----GGMSPGYYKTTCP--QLEDIVLKEVTRKK--NETIVTIPAVLRLFFHDCLV--NGCDASVLIASRNQD
85 18096|TaPrx109-1B|        93.9%  52.1%   ---EAAYG----GGMSPGYYKTTCP--QLEDIVLKEVTRKK--NETIVTIPAVLRLFFHDCLV--NGCDASVLIASRNED
86 18218|TaPrx109-1D|        93.9%  51.8%   ---GAAYG----GGMSPGYYKTTCP--QLEDIVLKEVTRKK--NETIVTIPAVLRLFFHDCLV--NGCDGSVLIASRNED
87 17919|TaPrx13-1A|         93.0%  55.0%   ---V-AVAAGGEAKLSPDHYRRTCP--RVESIVRSAVARKV--RATFVTVPATLRLFFHDCFV--QGCDASVMIASGSND
88 452|TaPrx13-1B|           93.0%  55.4%   ---VVAAASGGEAKLSPDHYRRTCP--RVESIVRSAVARKV--RATFVTVPATLRLFFHDCFV--QGCDASVMIASSSND
89 17920|TaPrx13-1D|         98.0%  40.9%   ---VAAAAAVGEAKLSPDHYRRTCP--RVESIVRSAVARKV--RATFVTVPATLRLFFHDCFV--QGCDASVMIASSSND
90 725|ZmPrx29|              93.6%  54.1%   ---AAITLPSAEAKLSPDYYRSTCP--DVEAIVQAVVAKKV--NETFVTVPATLRLFFHDCFV--EGCDASVIIASRDND
91 790|ZmPrx80|              94.4%  51.5%   ---MPSPP-RVAAALSPDYYKDSCP--GLESIVRYEVARKK--NETVVTIPATLRLVFHDCMV--GGCDAAVLIASKNND
   consensus/100%                        ................................................................................
   consensus/90%                         ............t.h..thY..pCs...hE.hl...l..hh..ttp..hh.uhhRhhFHDChl..tGCDuSlhltp.s..
   consensus/80%                         ...........tstL..saYt.oCP..tlE.lVtp.Vtthh..pps.shhPuhLRlaFHDChV..pGCDASlhlss.s..
   consensus/70%                         .......h..spupLs.saYtpoCP..sVEtIVpptVtpKh..ppThsThPATLRLFFHDCFV..pGCDASlhlsS.stD

                                 cov    pid 241       :         .         .         .         .    3    .         . 320
 1 17735|MdPrx10|               100.0% 100.0%   AEKNFADNLSLAGDGFDTVIKAKQAVEAQCPGVVSCADILAVAARDCVVLAGGPSEPVELGRRDGLISKASRVAGNLPEP
 2 16551|AtrPrx16|               94.4%  61.4%   AEKDASDNLSLAGDGFDTIVKAKAAVEALCPNVVSCADIMAIAARDVVVLAGGPNENVELGRRDNLQSKASLVAGNLPDP
 3 16793|EferPrx37|              97.7%  58.0%   AEKDAPDNLSLAGDGFDTVMKAKQAVEAVCPGIVSCADVLAIAARDVVSLSGGPVESVELGRRDGFVSLASNVPGNLPQP
 4 2273|AmajPrx06|               63.5%  31.0%   SERSSKASSAL--RGFEVIDDIKAEVEKQCPKVVSCADILTAAARDATVAVGGPYWTVPYGRKDGKVSTAKEADS-IPKG
 5 2275|AmajPrx08|               68.1%  31.8%   GERSAEASKTL--RGFTLIDEIKAAVEKQCPKIVSCADILTAAARDATVLVGGPHWEVPFGRKDGKVSIAKEANM-VPQG
 6 14835|AhalPrx54|              88.0%  37.0%   TERRSPASKSL--RGFELIDDIKSEMEKSCPGLVSCADILTSASRAATVQLGGPYWPNVYGRRDSKNSYARDVEK-VPSG
 7 7489|AlyPrx55|                94.7%  58.5%   AEKDAPDNKSLPGDGFDTVIKAKTAVESQCPGVVSCADILALAARDVVVIVGGPHEKVELGRRDGLVSQASRVTGKLPEP
 8 221|AtPrx55|                  94.7%  59.1%   AEKDADDNKSLAGDGFDTVIKAKTAVESQCPGVVSCADILALAARDVVVLVGGPHEKVELGRRDGLVSKASRVTGKLPEP
 9 15900|BvPrx47|                94.2%  67.3%   AEKDSPDDLSLAGDGFDTVIKAKQALEAACPGVVSCADILALAARDAVVLTGGPSESVELGRRDGLRSKASDVSGNLPEP
10 14968|BstrPrx41|              96.2%  60.2%   AEKDAPDNKSLAGDGFDTVIKAKTAVESQCPGVVSCADILALAARDVVVLVGGPHEKVELGRRDGLVSKASRVTGKLPEP
11 1293|BnPrx55-1_other|         91.2%  61.3%   AEKDAHDNISLAGDGFDTVAKAKLAVEAQCPGLVSCADILALAARDVVVLAGGPQEKVELGRRDGLVSHASSVDGKLPGP
12 6939|BnPrx55-2_other|         95.3%  61.4%   AEKDAHDNISLAGDGFDTVAKAKLAVEAQCPGLVSCADILALAARDVVVLAGGPQEKVELGRRDGLVSHASSVDGKLPGP
13 3019|BoPrx07-1Ca_other|       51.2%  33.3%   ------------------------------------------------YQLGGPYWPNAYGT----------------SG
14 3018|BoPrx07-1Cb_other|       61.1%  36.4%   SERRSPASKTL--RGFELIDDIKSEIEKSCPGLVSCADILTSASRSATYQLGGPYWPNAYGRRDSTNSYARDVEK-VPSG
15 3017|BoPrx07-2Ca_other|       88.0%  36.2%   SERRSPASKTL--RGFELIDDIKSEIEKACPGLVSCADILTAASRDATFQLGGPYWPNAYGRRDSTDSYARDVEK-VPSG
16 4252|BrPrx55-1Aa_other|       93.3%  65.2%   AEKDAHDNISLAGDGFDTVAKAKLAVEAQCPGLVSCADILALAARDVVVLAGGPQEKVELGRRDGLVSHASSVDGKLPGP
17 11240|BrPrx55-1Ab_other|      95.3%  59.7%   AEKDAQDNISLAGDGFDTVNKAKLAVEAQCPGRVSCADIMALAARDVVVLAGGPQEKVELGRRDGLVSNASRVDRKLPGP
18 14884|CagraPrx38|             96.2%  60.1%   AEKDAPDNKSLAGDGFDTVIKAKAAVEAQCPGVVSCADILALAARDVVVLVGGPQEKVELGRRDGLVSKASRVTGKLPEP
19 15070|CrubPrx70|              96.5%  61.5%   AEKDAPDNKSLAGDGFDTVIKAKAAVEAQCPGVVSCADILALAARDVVVLVGGPQEKVELGRRDGLVSKASRVTGKLPEP
20 8748|CpapPrx14|               93.9%  67.7%   AEKDAPDNLSLAGDGFDTIIKAKQAVEAQCPGVVSCADILALAAREVVGLAGGPSYSVELGRRDGLISQASRVRGALPEP
21 11285|CroPrx27|               95.6%  65.3%   AEKDAPDNLSLAGDGFDTVIKAKQAVEAQCPGIVSCADVLAIAARDVIVMAGGPSESVELGRKDGLVSKASRVAGNLPEP
22 8661|CclPrx100|               86.8%  37.9%   SERRAKVSKTL--RGFRIIDEIKAEVEKKCPKTVSCADILTAAARDATVVAGGPHWEVPFGRKDGKISSAREANRLVPQG
23 8700|CclPrx43|                93.9%  69.8%   AEKDAPDNLSLAGDGFDTVVQAKQAVEAQCPGVVSCADILAIAARDVVVLAGGASESVELGRRDGLVSRASSVKGNLPEP
24 8908|CsPrx20|                 94.7%  68.9%   AEKDAPDNLSLAGDGFDTVVQAKQAVEAQCPGVVSCADILAIAARDVVVLAGGAHESVELGRRDGLVSRASSVKGNLPEP
25 8814|CsaPrx24|                94.4%  66.1%   AEKDSEDNLSLAGDGFDTVIKAKQAVEAQCPGKVSCADILAIAARDVVVLAGGQNEAVELGRRDGLISKASLVAGNLPGP
26 8815|CsaPrx25|                95.3%  67.6%   AEKDAQDNLSLAGDGFDTVVKAKQAVEAACPGRASCADILALAARDVVVLAGGPNENVELGRRDGLISKASRVDGNLPSP
27 8816|CsaPrx27|                96.2%  66.7%   AEKDAKDNLSLAGDGFDTVVKAKQAVENVCPGLVSCADILALATRDVVNLAGGPQYSVELGRRDGLISQASRVAGNLPEP
28 10296|EcamPrx156|             94.2%  67.3%   AEKDAADNQSLAGDGFDTVIKAKQAVEASCQGVVSCADILAIAARDVVVLAGGPSENVELGRRDGLISKASQVAGKLPEP
29 10428|EcamPrx[P]183|          57.3%  26.3%   SERSAEVSKTL--RGFEIIDDIKTELEKSCLRTASCVDSLATAVRDATVSAGGPYWMVPYGCKDGRVSVAREADK-VPMG
30 10429|EcamPrx[P]99|           45.6%  35.4%   SERSAEASKTL--RGFEIINDINTELEKSCPRTALCADILTAATKDATVSAGGLY*MMPYGRKDGRVSVAGEADK-VPMG
31 10975|EglPrx156|              94.2%  66.7%   AEKDAPDNQSLAGDGFDTVIKAKQAVEASCQGVVSCADILAIAARDVVVLAGGPSENVELGRRDGLISKASLVAGKLPEP
32 10920|EglPrx[P]99|            39.5%  24.6%   ----------------------------------------------------------------GRVSVAGEADK-VPMG
33 8120|EgrPrx156|               94.4%  67.1%   AEKDAPDNQSLAGDGFDTVIKAKQAVEASCQGVVSCADILAIAARDVVVLAGGPSENVELGRRDGLISKASQVAGKLPEP
34 8050|EgrPrx[P]99|             70.5%  35.4%   NEKSAEASKTL--GGFEIIDDIKTELEKSCPRTTSCTDILTAAARDATVSAGGPYWMVPYGRKDGRVSVAGEADK-VPMG
35 11464|EguPrx156|              94.2%  67.0%   AEKDAPDNQSLAGDGFDTVIKAKQAVEASCQGVVSCADILAIAARDVVVLAGGPSENVELGRRDGLISKASLVAGKLPEP
36 11606|EguPrx[P]99|            70.5%  33.2%   NEKSAEVSKTL--KGFEIIDDIKTELEKSCPRTALCTDILTAAARDATVSAGGPYWMVPYGR*VGRVSVAGEANK-VPMG
37 9008|GmPrx272|                95.3%  67.2%   AEKDAEENISLPGDGFDTVIKAKQAVESSCPGVVSCADILALATRDVIGLLGGPSENVELGRKDGLISKASSVEGNLPKA
38 585|GmPrx56|                  95.3%  67.6%   TEKDAEENISLPGDGFDTVIKAKQAVEASCPGVVSCADILALATRDVIGLLGGPSENVELGRRDGLISKASSVEGNLPKA
39 9046|GmPrx[P]269|             90.9%  35.2%   SERTALESRTL--RGFQLIDNIKIELEKRCPRIVSCADILTAAARDATLMAGGPHWEVPFGRKDNKISLAREANM-VPHG
40 1478|HaPrx08|                 94.2%  68.7%   AEKDSSDNLSLAGDGFDTVIKAKQAVEAVCPGVVSCADVLAIAARDVVVAAGGPIENVQLGRRDGLISKASRVEGNLPLP
41 17498|IaquPrx45|              95.3%  70.2%   AEKDSSDNLSLAGDGFDTVVKAKQAVEAQCPGVVSCADILAIAARDVVVLAGGPAENVELGRRDGLVSKASRVEGNLPQP
42 17432|InPrx74|                94.2%  67.2%   AEKDSSDNLSLAGDGFDTVVKAKQAVEAQCPGVVSCADILAIAARDVVVMAGGPAENVELGRRDGLVSKASRVKGNLPEP
43 17614|InPrx[P]123|            92.1%  35.7%   SEKTAIPNLSV--RGYDLIDAIKEAVEKACTQVVSCADIIVMATRDAVSLSGGTRYIVQTGRKDGKVSLASNVDL--PKP
44 17640|KfedPrx47|              93.6%  61.5%   AEKDSETNLSLAGDGFDTVIKAKQAVEAVCPGIVSCADVLAIAARDVVTLSGGPSENVELGRRDGLISKAARVAGNLPEP
45 17648|KfedPrx55|              93.9%  57.0%   AEKDSDLNLTLPGDGFDTVIKAKQTVEAVCPGIVSCADVLAMATRDVVVLSGGPWEDVELGRRDGLVSEASRVAGKLPKP
46 3911|LperPrx49|              79.2%  33.8%   SEKTANVSKSL--RGFEVINDIKAAVEKKCPKTVSCADILTAAARDATVLAGGPHWTIPFGRKDGRVSLAKEAAK-VPMG
47 3912|LsPrx49|                71.9%  33.6%   SEKSANVSKSL--RGFEVINDIKAAVEKKCPKTVSCADILTAAARDATVLAGGPHWTIPFGRKDGRVSLAKEAAK-VPMG
48 3487|LsaPrx49|               61.1%  30.7%   SEKTSNMSKSL--RGFEVIDDIKAEIEKKCPKTVSCADILTTVARDATVLASGPYWTIPYGRKDGRVSLAKEAMI-VPKG
49 3909|LsePrx49|               82.2%  33.0%   SEKSANVSKSL--RGFEVINDIKAAVEKKCPKTVSCADILTAAARDATVLAGGPHWTIPFGRKDGRVSLAKEAAK-VPMG
50 3910|LvPrx49|                75.4%  33.9%   SEKSANVSKSL--RGFEVINDIKAAVEKKCPRTVSCADILTAAARDATVLAGGPHWTIPFGRKDGRVSLAKEAAK-VPMG
51 12117|LuPrx176|              93.9%  59.7%   AEKDAPDNLSLAGDGFDAVSRAKTAVEAICPGVVSCADIIAIAARDVVALAGGPTEAVELGRRDGMISSASLVAGNLPEP
52 12127|LuPrx191|              93.9%  59.6%   AEKDALDNLSLAGDGFDAVSRAKTAVEAICPGVVSCADIIAIAARDVVALAGGPTEAVELGRRDGMISSVSLVAGNLPEP
53 12179|LuPrx72|               96.5%  57.5%   AEKDAPENLSLAGDGFDTIIRAKTAVEAVCPGVVSCADIMAIAARDVLALVGGPSEKVELGRRDGVISSASLVAGNLPEP
54 636|LePrx38|                 94.2%  70.7%   AEKDSKDNLSLAGDGFDTVVKAKQAVEAQCPGVVSCADILAIATRDVVVLAGGPSYNVELGRRDGLISQASRVAGKLPEP
55 9122|MePrx64|                94.2%  70.1%   AEKDASDNLSLAGDGFDTVIKAKAAVEAQCPNVVSCADILAIAARDVVVLAGGPSESVELGRRDGLISKSSLVSGNLPEP
56 365|MtPrx41|                 93.0%  67.0%   AEKDAKENLSLPGDGFDTVIKAKQAVESVCPGVVSCADILAIATRDVIALLGGPSESVELGRRDGLNSKASNVEANLPKP
57 9255|MguPrx98|               93.0%  65.0%   AEKDSPDNLSLAGDGFDTVVKAKQAVEAQCPGIVSCADILALAARDVVLLAGGPAYNVELGRRDGLVSQASRVAGNLPDP
58 18385|NoffPrx55-1A|          95.6%  60.3%   AEKNAPDNISLAGDGFDTVIKAKTAVEALCPGVVSCADILALAARDVVVLAGGPQEKVELGRRDGLVSKASRVAGKLPGP
59 18448|NoffPrx55-1B|          95.3%  60.6%   AEKNAPDNISLAGDGFDTVIKAKTAVEALCPGVVSCADILALAARDVVVFAGGPQEKVELGRRDGLVSKASRVAGKLPGP
60 17107|NnPrx59|               94.7%  70.4%   AEKDAKDNLSLAGDGFDTVIKAKQAVEAVCPGVVSCADVLAIAARDVVVLAGGPSENVELGRRDGLISQASRVDGNLPQP
61 8252|NtPrx44-1A|             94.2%  72.2%   AEKDSSDNISLAGDGFDTVIKAKEAVEAQCPGVVSCADILAIATRDVVVLAGGPAYNVELGRRDGLISKASRVAGKLPEP
62 17698|NtPrx44-1B|            98.2%  66.9%   AEKDSSDNISLAGDGFDTVIKAKEAVEAQCPGVVSCADILAIATRDVVVLAGGPAYNVELGRRDGLISKASRVAGKLPEP
63 3099|PtPrx21|                93.9%  67.3%   AEKDAPDNLSLAGDGFDTVVKAKQKVEGACPGVVSCADILAIAARDVVVLAGGPSENVELGRRDGLVSKASLVKGNLPEP
64 3177|PtPrx82|                93.3%  67.3%   AEKDAPDNLSLAGDGFDTVVKAKQAVEAACPKVVSCADILALAARDVVVLAGGPSENVELGRRDGMVSQASLVKGNLPDP
```

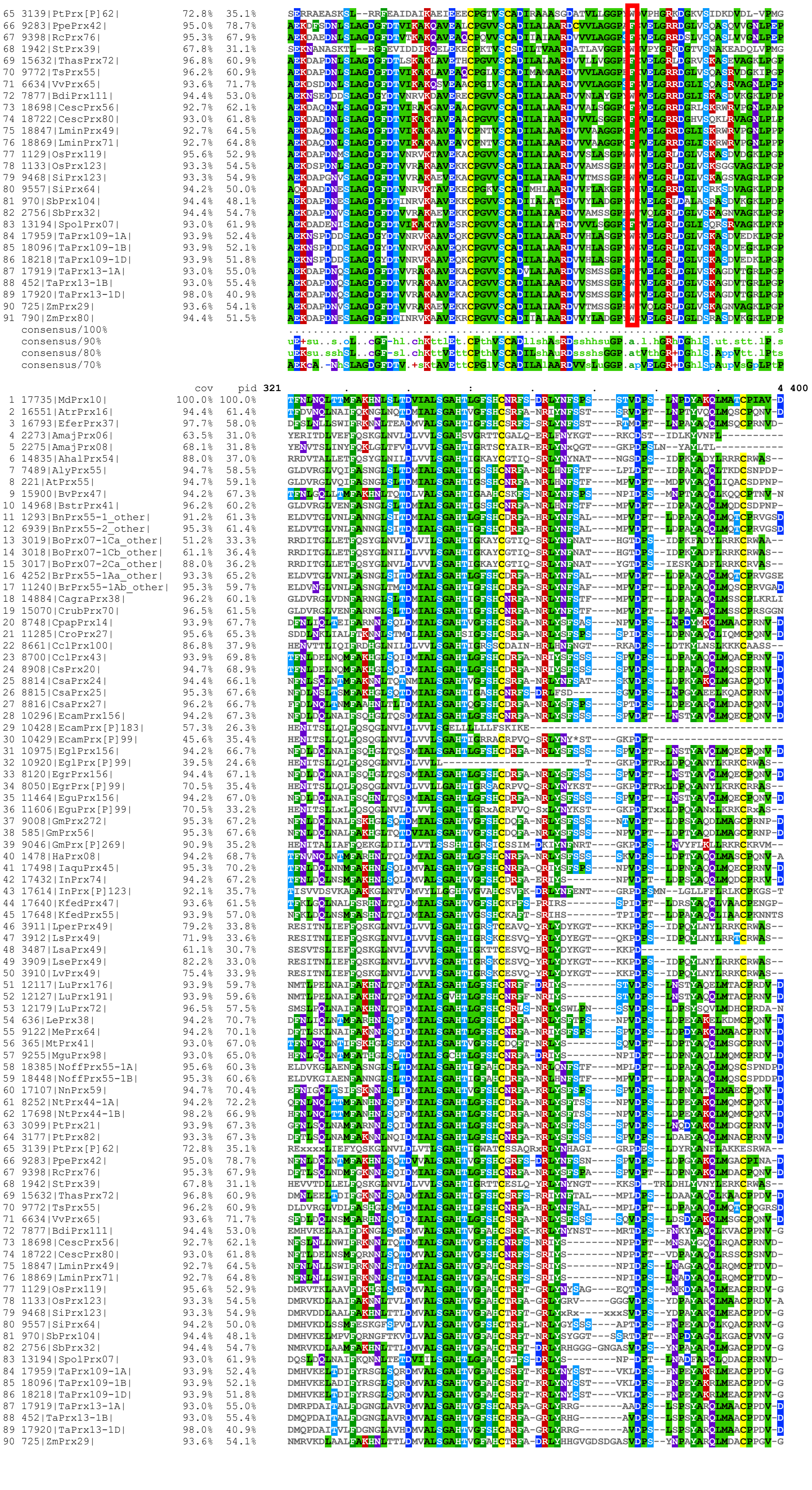

```
91 790|ZmPrx80|                 94.4%  51.5%      DMHVKDLTPVFQRNGFTEVDMVALSGAHTVGFAHCSRFT-DRLYSYGGA-----RTDPS--FNPAYAYQLKQACPIDV-G
   consensus/100%                                ..........a.t.shs..shlhL.......................................................
   consensus/90%                                 p.plttL.thFtppsLs.hDhlsLSGAHTlGhutCttht.tRlas..........DPo..hs.tYht.Lht.C...s..
   consensus/80%                                 phplptL.thFtppsLs.hDhlsLSGAHTlGhu+Cspht.pRlYsh.........sDPo..hsstYAp.LhttCs.ss..
   consensus/70%                                 phslspLsthFtppsLo.hDhlALSGAHTlGFoHCs+Fs.sRLYsaps.....t.sDPo..lsstYAppLhptCP.ss..

                              cov     pid 401        .         .         .         .         :         .         .         . 480
 1 17735|MdPrx10|           100.0% 100.0%      P-----NIVMTLDPDTPFTFDNAYYRNLVAGKGLLSSDQVLFSDSASRSTVLNFANNA-DNFNGAFVTAMRKLGRVGVKT
 2 16551|AtrPrx16|           94.4%  61.4%      P-----TIAINMDPITPRIFDNVYYQNLINGKGLFTSDEVLFTDARTSGVVRDFAANP-LDFSSAFAAAMIKLGRVGVKT
 3 16793|EferPrx37|          97.7%  58.0%      P-----TVAVQLDPVTGLMFDNVYYRNLVDGKGLLTSDQTLFNSRRTRKMVQDLANNQ-AKFFAAFASAMVKLGRLQVKT
 4 2273|AmajPrx06|           63.5%  31.0%      --------------------------------------------------------------------------------
 5 2275|AmajPrx08|           68.1%  31.8%      --------------------------------------------------------------------------------
 6 14835|AhalPrx54|          88.0%  37.0%      -------ETVELDAVTPAVFDNQYYINLQKHMGVLSTDQELVKDPRTAPLVKAFAEQPPQMFRQQFAVSMAKLVNVGVLT
 7 7489|AlyPrx55|            94.7%  58.5%      ------DFVVPLDPTTTDTFDNSYFQNLVARRGLLTSDQALFNDLSSQSTVMRFANNA-EEFYGAFSSAMRNLGRVGVKV
 8 221|AtPrx55|              94.7%  59.1%      ------DAVVDIDLTSRDTFDNSYYQNLVARKGLFTSDQALFNDLSSQATVVRFANNA-EEFYSAFSSAMRNLGRVGVKV
 9 15900|BvPrx47|            94.2%  67.3%      P-----NLVVFMDPRTPTTFDNKYYQDLVAGRGLFTSDQELYTNPASRPTVINFANNP-GNFNAAFIIGMKKLGRVGVKT
10 14968|BstrPrx41|          96.2%  60.2%      ------DTAISLDVATPNTFDNSYFQNLVARKGLLTSDQVLFNDLSSQSTVIRFANNA-GEFNSAFSSAMRKLGRVGVKV
11 1293|BnPrx55-1_other|     91.2%  61.3%      P-----NSVSNLDATTPDVFDNAYFLNLVARKGLFTSDQVLFNDFRSQAAVVRFANNA-EEFNNAFTSAMRKLGRVGVKV
12 6939|BnPrx55-2_other|     95.3%  61.4%      P-----NSVSNLDATTPDVFDNAYFLNLVARKGLFTSDQVLFNDFRSQAAVVRFANNA-EEFNNAFTSAMRKLGRVGVKV
13 3019|BoPrx07-1Ca_other|   51.2%  33.3%      -------ETVYLDVETPVVFDNQYYINLQKNMGVLTTDQELVKDPRTAPLVQAFPEQPAQMFRHQFAVSMAKLVNVGVIT
14 3018|BoPrx07-1Cb_other|   61.1%  36.4%      -------ETVYLDAVTPVVFDNQYYINLQKNMGVLTTDAELVKDPRTAPLLKAFAE------------------------
15 3017|BoPrx07-2Ca_other|   88.0%  36.2%      -------ETVYLDAETPVKFDNQYYINLQKNMGVLTTDQELVKDPRTAPLVKAFAEQPPHMFR*QFAVSMAKLVNVGVIT
16 4252|BrPrx55-1Aa_other|   93.3%  65.2%      P-----NVVSTLDATTPDVFDNAYFQNLVARKGLLTSDQVLFNDFRSQATVVRFANNA-EDFNSAFTSAMRKLGRVGVKV
17 11240|BrPrx55-1Ab_other|  95.3%  59.7%      P-----SVAVNLDLNTPNVFDNVYFQNLVARKGLFTSDQILFNDFRSQATVIRFANSA-EEFNRAFTSAMRKLGRVGVKV
18 14884|CagraPrx38|         96.2%  60.1%      LITFMSKSYTSLTLTTGRVFDNLYYQNLVARKGLFTSDQVLFDDLSTQSIVMRFANNA-GEFYAAFISAMRNLGRVGVKV
19 15070|CrubPrx70|          96.5%  61.5%      P-----AQVVEIDLTTGRVFDNLYYQNLVARKGLFTSDQVLFDDLSTQSIVMRFANNA-GEFYAAFISAMRNLGRVGVKV
20 8748|CpapPrx14|           93.9%  67.7%      P-----NIAIDMDPETPRTFDNVYYKNLVAGKGMFTSDQVLFTDSSTQPIVKDFAN---GDFSGAFVTAMTKLGRIGVKT
21 11285|CroPrx27|           95.6%  65.3%      P-----SIAVNIDPTTPTTFDNVYYQNLVGGKGLFTSDQELFTDPRTQSTVTDFANNP-LNFNAAFVTAMRNLGRVGVKT
22 8661|CclPrx100|           86.8%  37.9%      ------HVYVDLDATTPRIFDTAYYTNLGRNMGLLSTDQLLNSDARTGPFVSVLASQP-LVFSSQFAASMVKLGNIGVLA
23 8700|CclPrx43|            93.9%  69.8%      P-----QIAINMDPVTPRTFDNMYYQNLVAGKGLFTSDQVLFTDASSQPTVKDFARNP-LDFNAAFATAMRKLGRVGVKT
24 8908|CsPrx20|             94.7%  68.9%      P-----QIAINMDPVTPRTFDNMYYQNLVAGKGLFTSDQVLFTDASSQPTVNDFARNP-LDFNAAFATAMRKLGRVGVKT
25 8814|CsaPrx24|            94.4%  66.1%      P-----RIAVNMDPVTPRKMDNVYYQNLVNHKGLFTSDQVLYTDPLSQATVSGFANDR-SGFNKAFGEAMVQLGRVGVKT
26 8815|CsaPrx25|            95.3%  67.6%      P-----GVVVKLDPTTPDSFDNAYYRNLVEGKGLFRSDEVLFTNSASKGRVVGFANNK-GKFNGAFVKAMRKLGRVGVKT
27 8816|CsaPrx27|            96.2%  66.7%      P-----SVAINMDPITPQTFDNVYYQNLISGKGLFTSDQILFTESESQPTVSSFATNG-AEFNAAFITAMTKLGRVGVKT
28 10296|EcamPrx156|         94.2%  67.3%      P-----QIAINMDPVTPRTFDNMYYTNLVQGKGLFTSDETLFTDSASKPTVLNFANSP-GSFNQAFATAMRKLGRVGIKT
29 10428|EcamPrx[P]183|      57.3%  26.3%      --------------------------------------------------------------------------------
30 10429|EcamPrx[P]99|       45.6%  35.4%      --------------------------------------------------------------------------------
31 10975|EglPrx156|          94.2%  66.7%      P-----QIAINMDPVTPQTFDNMYYTNLVQGKGLFTSDETLFTDSASKPTVLNFANSP-GSFNQAFATAMRKLGRVGIKT
32 10920|EglPrx[P]99|        39.5%  24.6%      -------EYVDLDRTTPRAFDNAYYKNLQRKTGL----------------VAALATQP-DVFSYQFGVSMAKLGNVQVLT
33 8120|EgrPrx156|           94.4%  67.1%      P-----QIAINMDPVTPQTFDNMYYTNLVQGKGLFTSDETLFTDSASKPTVLNFANSP-GSFNQAFATAMRKLGRVGIKT
34 8050|EgrPrx[P]99|         70.5%  35.4%      -------EYVDLDRTTPRAFDNAYYKNLQRKTGLLSTDQYLYSDSRTGPLVAALATQP-DVFSYQFGVSMAKLGNVQVLT
35 11464|EguPrx156|          94.2%  67.0%      P-----QIAINMDPVTPRTFDNMYYTNLVQGKGLFTSDExLFTDSASKPTVLNFANSP-GSFNRAFATAMRKLGRVGIKT
36 11606|EguPrx[P]99|        70.5%  33.2%      -------EYVDLDRTTPRAFDNAYYKNLQRKTGLLSTDQYLxSDSRTGPLxAALATQP-DxFxYQFGVSMAKLGNVQVLT
37 9008|GmPrx272|            95.3%  67.2%      P-----TVAVALDPQSPAAFDNLYYQNLLSGKGLLTSDQVLFEDATSQPTVVRFANNV-ADFNDAFVAAIRKLARVGVKT
38 585|GmPrx56|              95.3%  67.6%      P-----AVVLPLDPQSPAAFDNAYYQNLLSGKGLLTSDQVLFEDATSQPTVVRFANSA-ADFNDAFVAAMRKLGRVGVKT
39 9046|GmPrx[P]269|         90.9%  35.2%      -------DLVHLDVITPRTFDTTYYTNLMRKVGLLSTDQSLFSDARTAPFVEAFAT*P-FLFTSQFSVSMVKLGNVHVLT
40 1478|HaPrx08|             94.2%  68.7%      P-----DIAINMDPVTAQTFDNVYYQNLVAGKGLFTSDQVLFTNPSTQSTVNDFANNP-GDFNGAFITAMRKLGQTGVKT
41 17498|IaquPrx45|          95.3%  70.2%      P-----TIAINMDPTTPRDFDNVYYQNLVAGKGLFTSDQVLFTDTNSRSTVVDFANNP-LDFSGAFVSAMRKLGRVGVKT
42 17432|InPrx74|            94.2%  67.2%      P-----TIAINMDPTTPRAFDNVYYQNLVAGKGLFTSDQVLFTDTNSRTTVMDFANNP-LDFNGAFASAMRKLGRVGVKI
43 17614|InPrx[P]123|        92.1%  35.7%      F-----DNTANLDQLTPKRVDNSFYQQLLLRNGVLQFDQQMALDSLTRDTVKAIAxxxxxxxxxxxxxxxxLGAVDVLT
44 17640|KfedPrx47|          93.6%  61.5%      A-----EVAVDMDPQTPRMFDNLYYQGLVQGKGMFTSDQDLFEDGDSKSTVVEFANSV-GEFYGAFGAAMRKLGRVGVKV
45 17648|KfedPrx55|          93.9%  57.0%      A-----DTIVDMDPQTPRKFDSLYYQELVQRKGLFTSDQDLFENGNSKGTVVDFASNG-GEFERAFAAAMRKLGRVGVKV
46 3911|LperPrx49|           79.2%  33.8%      -------ENVYLDATSPHKFDTQYYQNLKKKMGLLSTDQLLYGDSGLNDC------------------------------
47 3912|LsPrx49|             71.9%  33.6%      -------EDVYLDATTPHKFDTQYY-------------------------------------------------------
48 3487|LsaPrx49|            61.1%  30.7%      --------------------------------------------------------------------------------
49 3909|LsePrx49|            82.2%  33.0%      -------EDVYLDATTPHKFDTQYYQNLKRRWGLLSTDQLLYGDSGLNPDADALSFDS-SL-------------------
50 3910|LvPrx49|             75.4%  33.9%      -------EDVYLDATTPHKFDTQYYQNLKKKMGLLST-------------------------------------------
51 12117|LuPrx176|           93.9%  59.7%      P-----SIAIDMDPTTPRAFDNVYYQNLVEGKGMFSSDQVLFTDGRSRATVVDFARSA-ADFNGGFAAAMRKLGRVGVKT
52 12127|LuPrx191|           93.9%  59.6%      P-----SIAIDMDPTTPRAFDNVYYQNLVEGKGMFSSDQVLFTDGRSRATVVDFARSA-AAFNGGFVAAMRKLGRVGVKT
53 12179|LuPrx72|            96.5%  57.5%      P-----EAIADMDPTTPRRFDNAYYQNLVGGKGMFTSDQVLFTDSRSQPTVLDFANNA-GDFNAAFTVAMRKLGRAGVKT
54 636|LePrx38|              94.2%  70.7%      P-----TIAINMDPATPRTFDNEYYKNLVKGKGLFTSDQVLFTDESSQGTVSDFANNA-FDFNGAFVTAMRKLGRVGVKT
55 9122|MePrx64|             94.2%  70.1%      P-----VIAIDMDPTTPRTFDNVYYQNLVAGKGLFTSDEVLFTDPASKPMVVDFANSP-GDFNGAFVTAMRKLGRVGVKT
56 365|MtPrx41|              93.0%  67.0%      P-----NIVLALDTQTEHTFDNLYYKNLVNGKGLLSSDQVLFTDDASRSTVVEFANDG-SKFFEAFVVAIKKLGRVGVKT
57 9255|MguPrx98|            93.0%  65.0%      P-----RIAIDMDPVTPRTFDNVYYQNLVGGKGLFTSDQVLFTDPASQPTVLGFAQNP-GDFNGAFITAMTKLGRVGVKT
58 18385|NoffPrx55-1A|      95.6%  60.3%      LA----NVAVNLDLNTPNTFDNLYYQNLVARRGLFTSDQALFNDFSSQSTVIRFANNA-GDFNGAFISAMRKLGRVDVKV
59 18448|NoffPrx55-1B|      95.3%  60.6%      LA----NVAVNLDLSTPNTFDNLYYQNLVARRGLFTSDQALFNDFSSQSTVIRFANNA-GDFNGAFISAMRKLGRVGVKV
60 17107|NnPrx59|            94.7%  70.4%      P-----TIAVNMDPVTPNKFDNVYYQNLVAGKGLFTSDEVLFTNPASQSTVKDFASNP-FNFNAAFATAMVNLGRVGVKT
61 8252|NtPrx44-1A|          94.2%  72.2%      P-----SIAINMDPVTPRTFDNEYYKNLVGGKGLFTSDQVLFTDEASQRTVNDFANNA-FDFNGAFVTAMRKLGRVDVKT
62 17698|NtPrx44-1B|         98.2%  66.9%      P-----SIAINMDPVTPRTFDNEYYKNLVGGKGLFTSDQVLFTDEASQRTVNDFANNA-FEFNGAFVTAMRKLGRVGVKT
63 3099|PtPrx21|             93.9%  67.3%      P-----SIAINMDPVTPQTFDNVYFQNLVNGKGLFTSDEVLFTDPASQPTVKDFANSS-SDFNGAFATAMRKLGRVRVKT
64 3177|PtPrx82|             93.3%  67.3%      P-----SIAIDMDPVTSRTFDNVYFQNLVSGKGLFTSDEVLFSDPASQPTVNDFAKNS-GDFNGAFATAMRKLGRVGVKT
65 3139|PtPrx[P]62|          72.8%  35.1%      ------FEYVDRDARTPQTFDSVCFKNLQDHTRLLSTDQSLYSDPRTSPIVDASADGP-EFFNHQFAVSMTKLGNILVPT
66 9283|PpePrx42|            95.0%  78.7%      Q-------VINLDPETPDTFDNAYYRNLVAGKGLLSSDQVLFSDSASRPTVIDFANNP-GNFNGAFITAMRKLGRVGVKT
67 9398|RcPrx76|             95.3%  67.9%      P-----VIAVDMDPTTPRIFDNVYYQNLVAGKGLFTSDQVLFTDPSSKSTAIDFANSE-GEFNGAFVTAMRKLGRVGIKT
68 1942|StPrx39|             67.8%  31.1%      -------GIWLILMLL----------------------------------------------------------------
69 15632|ThasPrx72|          96.8%  60.9%      P-----TIAIDMDPTTPRVFDNVYYQNLVARKGLFSSDQILFTHPASQPTVFTFSNNP-DAFNAAFASAMRKLGRVGVKS
70 9772|TsPrx55|             96.2%  60.9%      P-----EIALDLDITTPRVFDNVYFQNLVARKGLFTSDQVLFNDFRSQATVVRFANSA-EEFNSAFISAMRKLGRVGVKV
71 6634|VvPrx65|             93.6%  71.7%      P-----SIAIDMDPVTPRTFDNEYYQNLVAGKGLFTSDEALFSDPSSQPTVTDFANSP-GEFNGAFITAMRKLGRVGVKT
72 7877|BdiPrx111|           94.4%  53.0%      P-----TIAVNMDPLSPVTFDNKYYNNLVNGLGLFTSDQVLYTDVASKKTVEEFNASQ-DQFFKAFVDSMIKLGRVDVK-
73 18698|CescPrx56|          92.7%  62.1%      P-----RVAINMDPTTPNVFDNAYFRNLVNGQGLFTTDQVLFDNTQSRGTVLNFANSR-TNFFNAFAAAMVRLGRTGVKT
74 18722|CescPrx80|          93.0%  61.8%      P-----SVAVNMDPTTPNVFDNAYYSNLVARKGMFTSDEVLFSSSQSRGTVVDFANNP-ADFATAFAAAMVKLGRTGLKT
75 18847|LminPrx49|          92.7%  64.5%      P-----SIAINMDPTTPNAFDNVYFKNLVDGKGLFSSDQVLFTNPISRRTVVNFANSQ-ASFFNAFTSAMVKLGRTGVKT
76 18869|LminPrx71|          92.7%  64.8%      P-----SIAINMDPTTPNAFDNVYFKNLVDGKGLFSSDQVLFTNPISRRTVVNFANSQ-ASFFNAFTSAMVKLGRTGVKT
77 1129|OsPrx119|            95.6%  52.9%      K-----TIAVNMDPVSPIVFDNVYYSNLVNGLGLFTSDQVLYTDGASRRTVEEFAVNQ-TAFFDAFVSSMVRLGRLGVK-
78 1133|OsPrx123|            93.3%  54.5%      P-----TIAVNMDPITPAAFDNAYYANLAGGLGLFTSDQELYTDAASRPAVTGFAKNQ-TLFFEAFKEAMVKLGRVGVK-
79 9468|SiPrx123|            93.3%  54.9%      A-----TIAVDMDPVTPTAFDNAYYGNLAGGMGLFTSDQALYSDGASRPAVGDFAKNQ-TRFFEAFKDAMVKLGRVGVK-
80 9557|SiPrx64|             94.2%  50.0%      E-----TIAVNMDPVSPITFDNKYYSNLQYGLGLFTSDQVLYTDDSTKPIVNKFAADQ-KAFFDAFGLAMVKLGRLGVK-
81 970|SbPrx104|             94.4%  48.1%      P-----TIAVNMDPVSPIKFDNIYFINLQYGLGLFTSDQVLYTDETTRPIVDKFAASQ-KEFFDAFVAAMIKLGRLGVK-
82 2756|SbPrx32|             94.4%  54.7%      A-----DIAVDMDPITPTAFDNAYYANLAGGLGLFASDQALYSDGASRPAVRGFAGNQ-TRFFEAFKDAMVKLGSVGVKT
83 13194|SpolPrx07|          93.0%  61.9%      P-----LVAINLDVNTPRTFDNVFFSNLVEGKGLLRSDQVLFSDPRSRSKVVEFAGNA-AGFGAAFADAMVRLGRVGVKT
84 17959|TaPrx109-1A|        93.9%  52.4%      P-----TIAVNMDPFTPVVFDNIYYQNLRNGIGLFTSDQALFTDGGSRKTVEEFADSE-PRFFQAFVESMMKVGRLGVK-
85 18096|TaPrx109-1B|        93.9%  52.1%      P-----TIAVNMDPFSPVLFDNIYYQNLRNGLGLFTSDQALFTDGGSRKTVEEFADSE-PRFFQAFVESMMKVGRLGVK-
86 18218|TaPrx109-1D|        93.9%  51.8%      P-----TIAVNMDPFSPVVFDNIYYQNLRNGIGLFTSDQALFTDGGSRKTVEEFADSE-PRFFQAFVESMMKVGRLGVK-
87 17919|TaPrx13-1A|         93.0%  55.0%      P-----TIAVDMDPVTPTVFDNKYYANLAAGLGLFASDQALHDGAASRPAVEGFAGNQ-TLFFEAFKEAMVKLGRVGVK-
88 452|TaPrx13-1B|           93.0%  55.4%      P-----TIAVDMDPVTPTVFDNKYYANLAAGLGLFASDQALHDGAASRPAVEGFAGNQ-TLFFEAFKEAMVKLGRVGVK-
89 17920|TaPrx13-1D|         98.0%  40.9%      P-----TIAVDMDPVTPTVFDNKYYANLAAGLGLFASDQALHDGAASRPAVQGFAGNQ-TLFFEAFKEAMVKLGRVGVK-
90 725|ZmPrx29|              93.6%  54.1%      A-----DIAVNMDPITPTAFDNAYYANLAGGLGLFTSDQALYSDGASQPAVRDFAKNQ-TRFFEAFKDAMVKLGSVGVK-
91 790|ZmPrx80|              94.4%  51.5%      P-----TIAVNMDPVSPIRFDNAYYANLQDGLGLFTSDQVLYADEATRPIVDMFAASQ-KDFFDAFVAAMLKLGRLGVK-
   consensus/100%                                ................................................................................
   consensus/90%                                 ........hh.hD..os.hFDs.Ya.sL..thGhhtoDt.h..t...t.....s.........................
   consensus/80%                                 ........slphDs.TP.hFDN.YatNLhtthGLhooDp.Lass.top.hV.tFAtp....F.ttFh.uMhpLGplsVh.
   consensus/70%                                 ......phslshDshTPpsFDNhYYpNLhtthGLhoSDQsLasDstopstV.tFAss..ttF.tAFhsAMhKLGRlGVKs

                              cov     pid 481        .         5         .    ] 516
 1 17735|MdPrx10|           100.0% 100.0%      GN--QGEIRRDCTTFNS*------------------
 2 16551|AtrPrx16|           94.4%  61.4%      GS--QGQVRKDCTVFNS*------------------
 3 16793|EferPrx37|          97.7%  58.0%      GS--EGEIRSNCAAFN--------------------
 4 2273|AmajPrx06|           63.5%  31.0%      ------------------------------------
 5 2275|AmajPrx08|           68.1%  31.8%      ------------------------------------
 6 14835|AhalPrx54|          88.0%  37.0%      GEDRIGEIRKVCSKSNSRRY*---------------
 7 7489|AlyPrx55|            94.7%  58.5%      GS--EGEIRRDCSAFN--------------------
 8 221|AtPrx55|              94.7%  59.1%      GN--QGEIRRDCSAFN--------------------
 9 15900|BvPrx47|            94.2%  67.3%      GN--QGEIRRNCAAFNS-------------------
10 14968|BstrPrx41|          96.2%  60.2%      GN--QGEIRRDCSAFN*-------------------
11 1293|BnPrx55-1_other|     91.2%  61.3%      ------------------------------------
12 6939|BnPrx55-2_other|     95.3%  61.4%      GN--RGEIRKDCAAFN--------------------
13 3019|BoPrx07-1Ca_other|   51.2%  33.3%      AEDRIGEIRRVCSKSNSRP-----------------
14 3018|BoPrx07-1Cb_other|   61.1%  36.4%      ------------------------------------
15 3017|BoPrx07-2Ca_other|   88.0%  36.2%      GEERVGVIRKVCSKSNSRPY----------------
16 4252|BrPrx55-1Aa_other|   93.3%  65.2%      GK--QGEIRKDCSAFN--------------------
17 11240|BrPrx55-1Ab_other|  95.3%  59.7%      GS--QGEIRRDCSAFN--------------------
18 14884|CagraPrx38|         96.2%  60.1%      GN--QGEIRRDCSAFN*-------------------
19 15070|CrubPrx70|          96.5%  61.5%      GN--QGEIRRDCSAFN*-------------------
```

```
20 8748|CpapPrx14|            93.9%  67.7%   GT--EGEIRKDCTAFNS*------------------
21 11285|CroPrx27|            95.6%  65.3%   NNQ-QGEIRRDCTTFNS-------------------
22 8661|CclPrx100|            86.8%  37.9%   RPN-EGEIRFKCSSVNRAY*----------------
23 8700|CclPrx43|             93.9%  69.8%   GN--QGEIRRDCTAFN--------------------
24 8908|CsPrx20|              94.7%  68.9%   GN--QGEIRRDCTAFN*-------------------
25 8814|CsaPrx24|             94.4%  66.1%   GA--AGEIRKDCTAFN*-------------------
26 8815|CsaPrx25|             95.3%  67.6%   GK--AGEIRRDCTAFN--------------------
27 8816|CsaPrx27|             96.2%  66.7%   GN--DGEIRRDCTAFNS*------------------
28 10296|EcamPrx156|          94.2%  67.3%   GS--QGEIRRDCTAFNS-------------------
29 10428|EcamPrx[P]183|       57.3%  26.3%   ------------------------------------
30 10429|EcamPrx[P]99|        45.6%  35.4%   ------------------------------------
31 10975|EglPrx156|           94.2%  66.7%   GS--QGEIRRDCTAFNS-------------------
32 10920|EglPrx[P]99|         39.5%  24.6%   G-D-EGEVRTEARSAPTATPAIATNYFSKFIRICLL
33 8120|EgrPrx156|            94.4%  67.1%   GS--QGEIRRDCTAFNS*------------------
34 8050|EgrPrx[P]99|          70.5%  35.4%   G-D-EGEVRTEARSAPTATPAIATNYFSKFIRICLL
35 11464|EguPrx156|           94.2%  67.0%   GS--QGEIRRDCTAFNS-------------------
36 11606|EguPrx[P]99|         70.5%  33.2%   x-D-EGEVRTEARSAPTATPAIATNYFSKFIRICLL
37 9008|GmPrx272|             95.3%  67.2%   GN--DGEIRRDCTTFNS*------------------
38 585|GmPrx56|               95.3%  67.6%   GK--DGEIRRDCTTFNS*------------------
39 9046|GmPrx[P]269|          90.9%  35.2%   RPN-EGEIRVNCNYVNIV------------------
40 1478|HaPrx08|              94.2%  68.7%   GN--QGEIRRDCTAFN--------------------
41 17498|IaquPrx45|           95.3%  70.2%   GS--QGEIRLDCTKFNS-------------------
42 17432|InPrx74|             94.2%  67.2%   GS--QGEIRLDCIKFNS-------------------
43 17614|InPrx[P]123|         92.1%  35.7%   GS--YGEIRKSCRLVNT-------------------
44 17640|KfedPrx47|           93.6%  61.5%   GA--EGEIRRDCAAFNG-------------------
45 17648|KfedPrx55|           93.9%  57.0%   GA--EGEIRRDCAAFNG-------------------
46 3911|LperPrx49|            79.2%  33.8%   ------------------------------------
47 3912|LsPrx49|              71.9%  33.6%   ------------------------------------
48 3487|LsaPrx49|             61.1%  30.7%   ------------------------------------
49 3909|LsePrx49|             82.2%  33.0%   ------------------------------------
50 3910|LvPrx49|              75.4%  33.9%   ------------------------------------
51 12117|LuPrx176|            93.9%  59.7%   GS--QGRIRSDCSNVNS*------------------
52 12127|LuPrx191|            93.9%  59.6%   GS--QGRIRSDCSNVNS-------------------
53 12179|LuPrx72|             96.5%  57.5%   GG--EGSIRSDCTIMN*-------------------
54 636|LePrx38|               94.2%  70.7%   GG--QGEIRLDCTRFNS*------------------
55 9122|MePrx64|              94.2%  70.1%   GS--QGRIRTDCTILNS-------------------
56 365|MtPrx41|               93.0%  67.0%   GK--EGEIRRDCSKFN--------------------
57 9255|MguPrx98|             93.0%  65.0%   GD--QGEIRRDCSAFNS-------------------
58 18385|NoffPrx55-1A|        95.6%  60.3%   GK--QGEIRRDCSAFN--------------------
59 18448|NoffPrx55-1B|        95.3%  60.6%   GK--QGEIRRDCSAFN--------------------
60 17107|NnPrx59|             94.7%  70.4%   GA--QGQIRRDCTAFNS-------------------
61 8252|NtPrx44-1A|           94.2%  72.2%   GN--QGEIRLDCTRFNS*------------------
62 17698|NtPrx44-1B|          98.2%  66.9%   GN--QGEIRLDCTRFNS-------------------
63 3099|PtPrx21|              93.9%  67.3%   GS--QGSIRTDCTVINS-------------------
64 3177|PtPrx82|              93.3%  67.3%   GS--QGTIRTDCTVINS-------------------
65 3139|PtPrx[P]62|           72.8%  35.1%   VQD-GGEIRTRCYSVNSNY-----------------
66 9283|PpePrx42|             95.0%  78.7%   GD--QGQIRTDCTTFNS*------------------
67 9398|RcPrx76|              95.3%  67.9%   GN--QGRIRTDCTNIDS-------------------
68 1942|StPrx39|              67.8%  31.1%   ------------------------------------
69 15632|ThasPrx72|           96.8%  60.9%   VP--LGEIRTDCSSFNS-------------------
70 9772|TsPrx55|              96.2%  60.9%   GS--QGEIRRDCSAFN*-------------------
71 6634|VvPrx65|              93.6%  71.7%   GD--QGEIRKDCTAFNS-------------------
72 7877|BdiPrx111|            94.4%  53.0%   TGS-AGEIRRDCTAFNH-------------------
73 18698|CescPrx56|           92.7%  62.1%   GN--QGEIRRDCSAFN--------------------
74 18722|CescPrx80|           93.0%  61.8%   GE--QGEIRRDCAAFN--------------------
75 18847|LminPrx49|           92.7%  64.5%   GS--QGEIRRDCTRFN--------------------
76 18869|LminPrx71|           92.7%  64.8%   GS--QGEIRRDCTRFN--------------------
77 1129|OsPrx119|             95.6%  52.9%   AGK-DGEVRRDCTAFNH-------------------
78 1133|OsPrx123|             93.3%  54.5%   SGK-HGEIRRDCTAFN--------------------
79 9468|SiPrx123|             93.3%  54.9%   TGR-HGEIRRDCTAFNK-------------------
80 9557|SiPrx64|              94.2%  50.0%   TGT-DGEIRRVCTAFNHY*-----------------
81 970|SbPrx104|              94.4%  48.1%   TGK-DGEIRRVCTAFNH-------------------
82 2756|SbPrx32|              94.4%  54.7%   TGR-HGEIRRDCTAFNE-------------------
83 13194|SpolPrx07|           93.0%  61.9%   GS--GGEIRRDCAAFN*-------------------
84 17959|TaPrx109-1A|         93.9%  52.4%   TGG-GGEIRRDCTAFNH-------------------
85 18096|TaPrx109-1B|         93.9%  52.1%   TGS-GGEIRRDCTAFNH-------------------
86 18218|TaPrx109-1D|         93.9%  51.8%   TGS-GGEIRRDCTAFNH-------------------
87 17919|TaPrx13-1A|          93.0%  55.0%   SGG-DGEIRRDCTAFN--------------------
88 452|TaPrx13-1B|            93.0%  55.4%   SGG-DGEIRRDCTAFN--------------------
89 17920|TaPrx13-1D|          98.0%  40.9%   SGG-DGEIRRDCTAFNSK------------------
90 725|ZmPrx29|               93.6%  54.1%   TGR-HGEIRSDCTAFN--------------------
91 790|ZmPrx80|               94.4%  51.5%   TGK-DGEIRRVCTAFNH-------------------
   consensus/100%                            ....................................
   consensus/90%                             ....................................
   consensus/80%                             st..tGpIRhsCs.hN....................
   consensus/70%                             ss..pGEIRpDCoshN....................
```
