# Supplementary material for: Target enrichment sequencing coupled with GWAS identifies MdPRX10 as a candidate gene in the control of budbreak in apple
Source: Front Plant Sci. 2024 Feb 21;15:1352757. doi: 10.3389/fpls.2024.1352757 (PMC10918860; doi:10.3389/fpls.2024.1352757)
Supplement: Supplementary file 1 [file DataSheet_1.zip › Supplementary Material/Supplementary Tables S2 and S7 and Supplementary Figures S1 - S9.PDF]

Supplementary Material

Table S2. Linear mixed models selected for extraction of genotypic BLUPs of days to budbreak from Jan 1st for each year from 2015 to 2023, based on lowest BIC of tested models. Green factors are random and red are fixed.

**Year**	**Mixed model components**
2015	Budbreak = Genotype
2016	Budbreak = Genotype
2017	Budbreak = Genotype
2018	Budbreak = Genotype + Row + Planting date
2019	Budbreak = Genotype + Row + Planting date
2020	Budbreak = Genotype + Row + Planting date
2021	Budbreak = Genotype + Row + Planting date
2022	Budbreak = Genotype + Row + Planting date
2023	Budbreak = Genotype + Row + Planting date

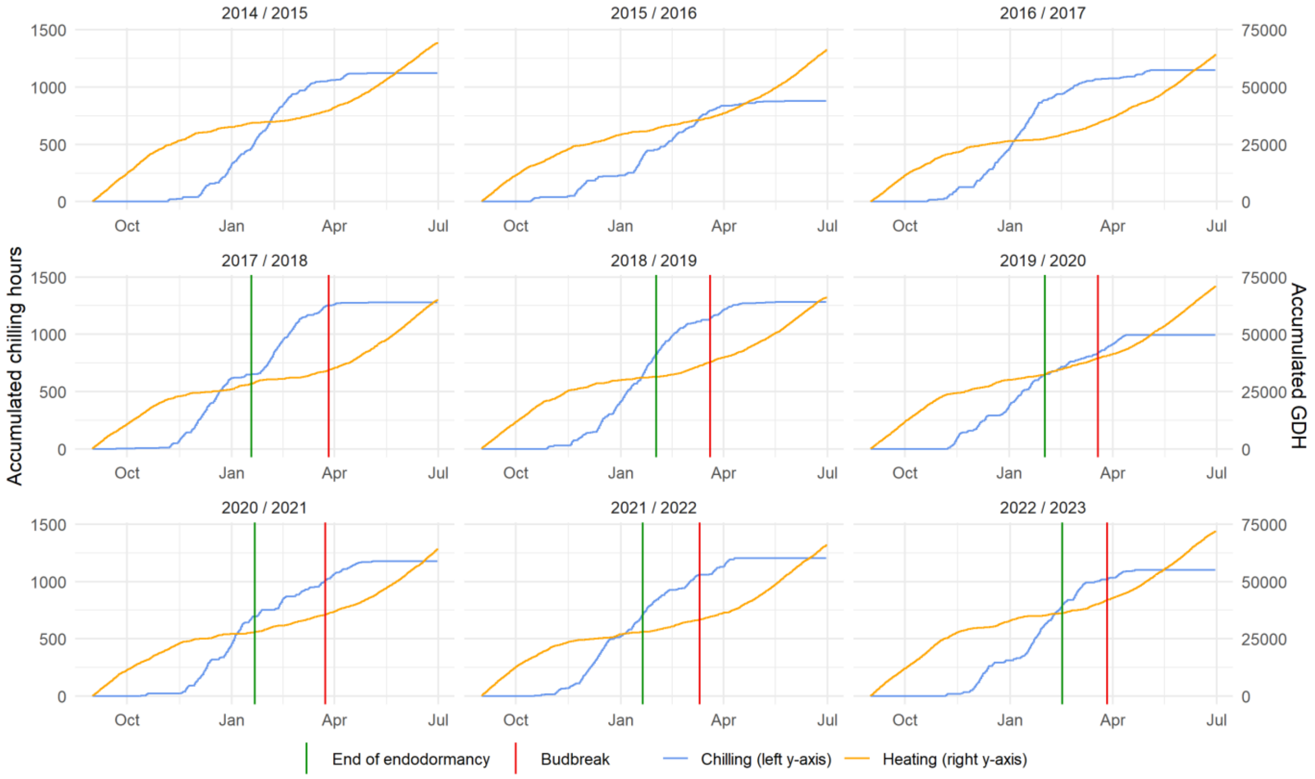

Figure S1. Accumulated chilling hours (CH, left axis, blue line) and growing degree hours (GDH, right axis, orange line) from September 1st till June 30th for each year from 2014 – 2023 at the Diascope experimental orchard. Mean timing of endodormancy release (and satisfaction of the chilling requirement) from all replicates of Gala in the orchard, as determined with the Tabuenca test in 2018 to 2023, is indicated by the green vertical line. Mean timing of budbreak (and satisfaction of the heating requirement of ecodormancy) from all Gala replicates in the orchard is shown by the red vertical line. CH were calculated with the chillR R package, which uses the method proposed by Bennett (1949), where any hour with a temperature between 0 and 7.2 °C is considered one CH. GDH were calculated with the same package, using the GDH model suggested by Anderson et al. (1986).

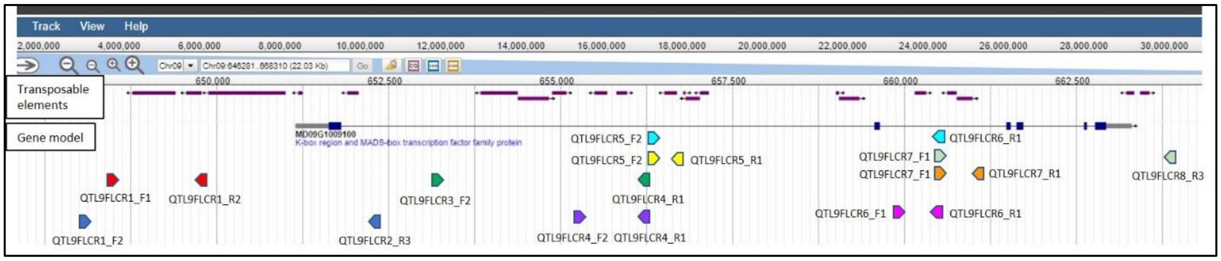

Figure S2. *MdFLC-like* (MD09G1009100; Chr09: 651155 – 663313 bp), as in the GDDH13 genome v1.1 browser (https://iris.angers.inra.fr/gddh13), showing the locations of the primer pairs used to amplify regions containing transposons. Primers of the same colour represent a pair. Primer sequences are given in Table S3.

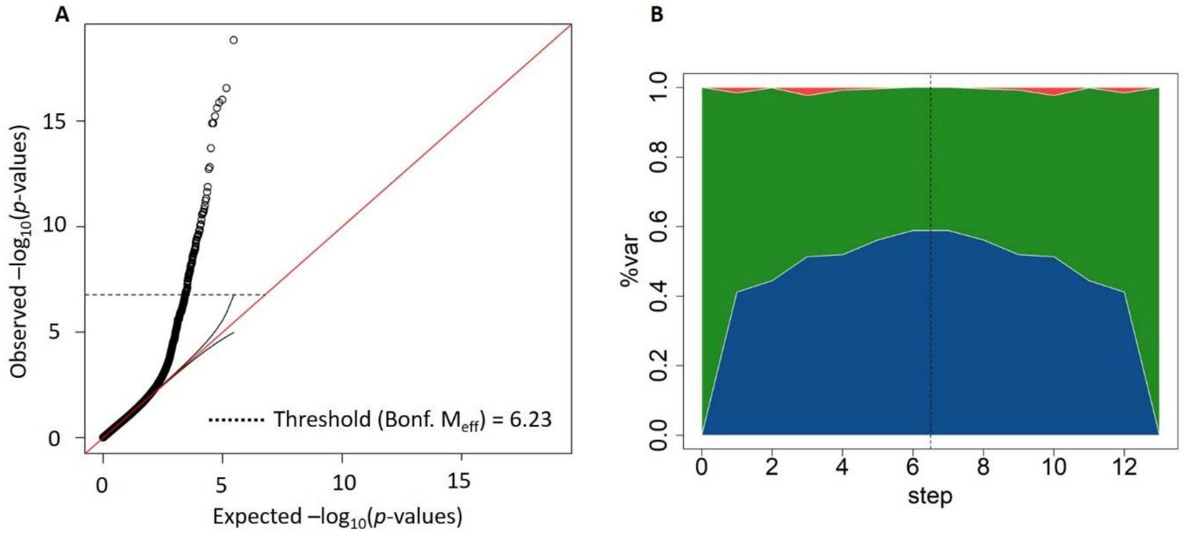

Figure S3. A. Q-Q plot from the GEMMA GWAS analysis, where the Bonferroni (Bonf.) threshold was calculated with the effective number of independent tests (M_{eff}). **B.** Partition of variance plot from the MLMM GWAS analysis with genotypic BLUPs of days to budbreak. Plot indicates percentage variance explained by SNP(s) (blue), kinship (green) and error (red).

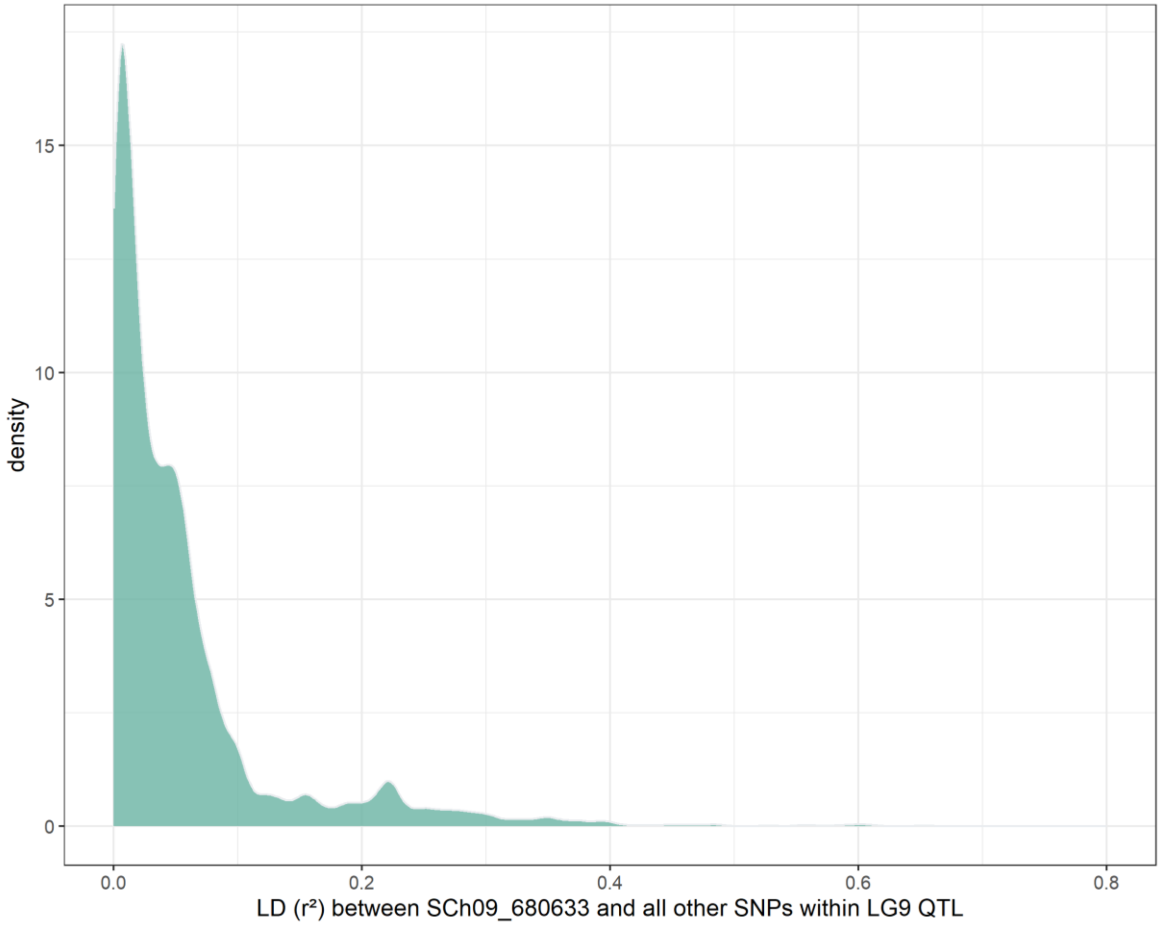

Figure S4. Density plot showing the linkage disequilibrium (LD; r^2) between the SChr09_680633 SNP and all other SNPs within the LG9 QTL interval (254 255 bp – 3 509 888 bp). The interval is defined as the region containing significant associations between a SNP and timing to budbreak according to the GEMMA GWAS analysis with threshold $-\log_{10}(p\text{-value}) = 6.23$.

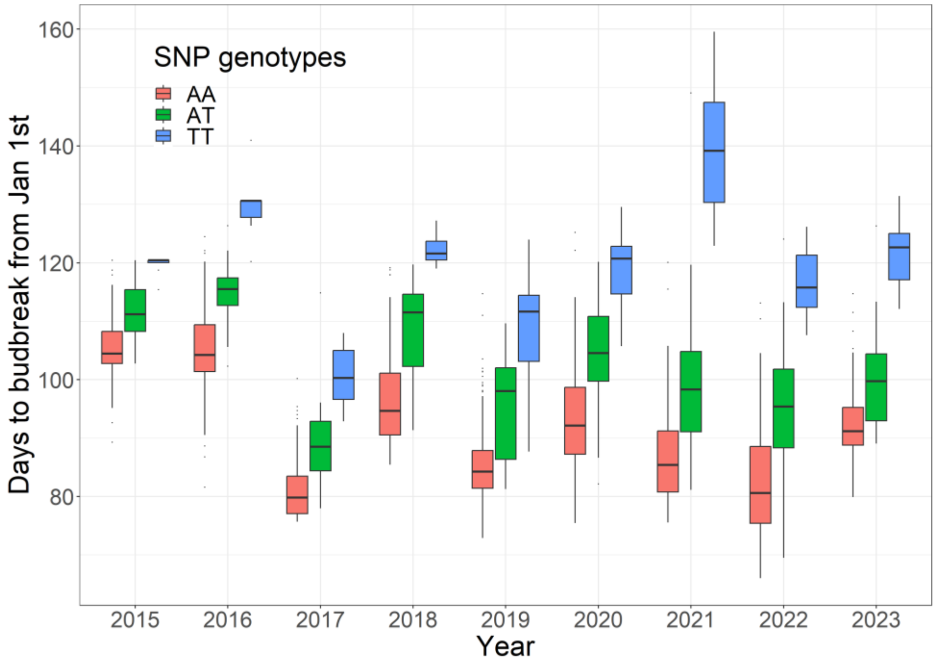

Figure S5. Boxplots of genomic BLUPs of days to budbreak from January 1st for each year from 2015 to 2023 for the three different allele genotypes of the SChr09_680633 SNP in the 239-cultivar apple core collection. Color indicates the alleles present at the position of the SNP, specifically, homozygous for the reference allele, A (red), homozygous for the alternative T allele (blue) or heterozygous with a copy of each allele (green).

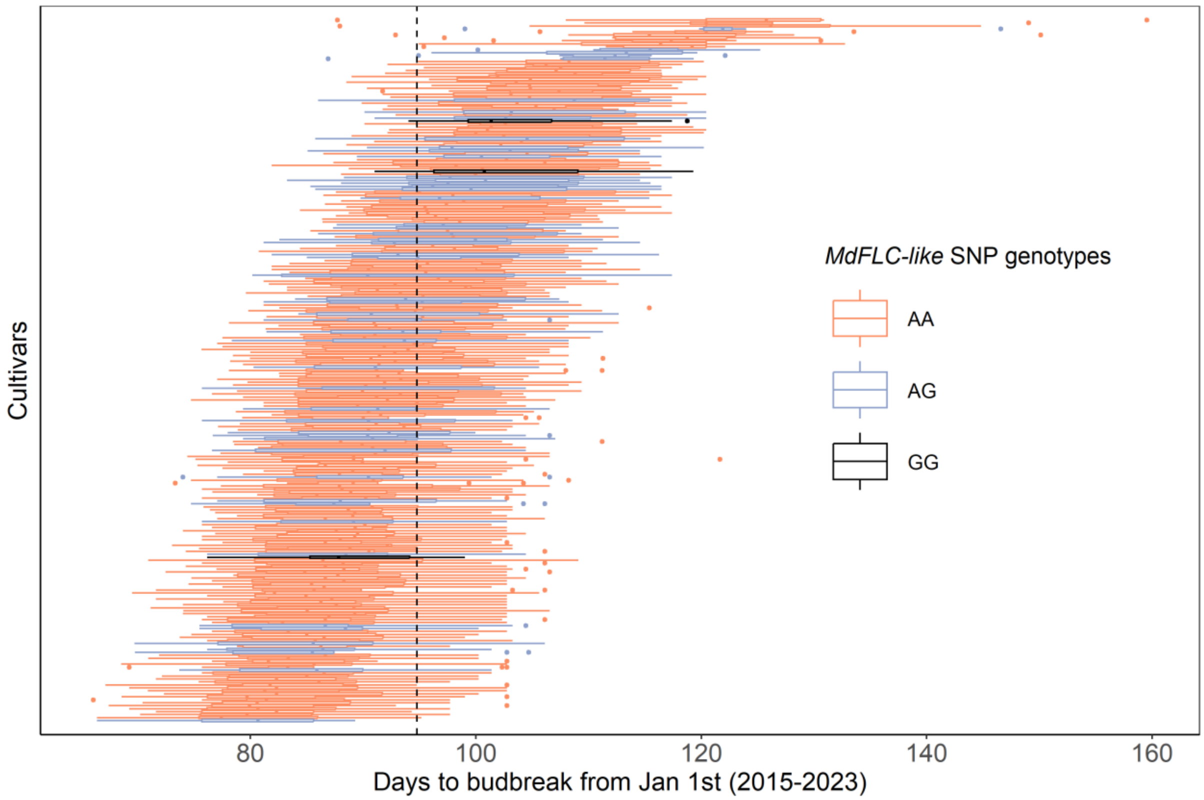

Figure S6. Stacked boxplots of genotypic BLUPs of days to budbreak from January 1st for each year from 2015 to 2023 for each of the 239 cultivars of the core collection (i.e. nine BLUPs per cultivar). Plots are ordered by mean from earliest to latest budbreak. Color indicates the alleles present in the AX.115485819 SNP of the *MdFLC-like* gene, specifically, homozygous for the reference allele, A (orange; 182 cultivars), homozygous for the alternative G allele (black; 3 cultivars) or heterozygous with a copy of each (blue; 52 cultivars). Dashed line is the mean budbreak date across all years and cultivars (94 days).

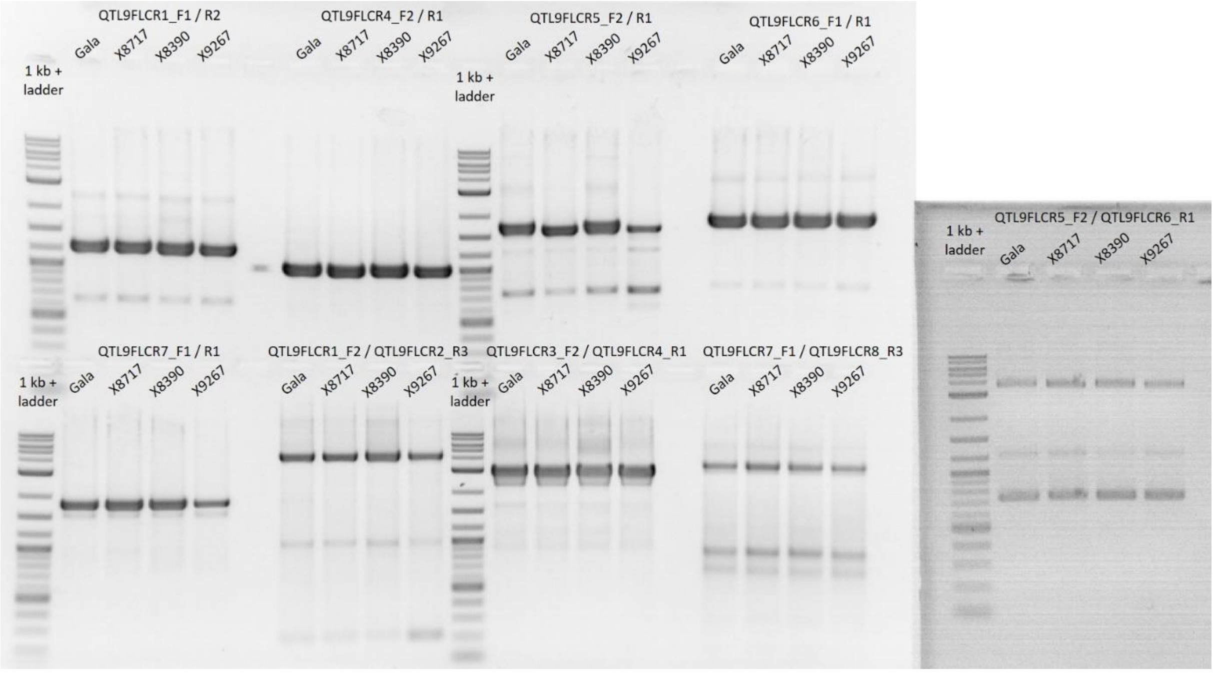

Figure S7. Two agarose electrophoresis gels (1.4 %) visualized under UV light following ethidium bromide staining showing PCR products spanning the length of the *MdFLC-like* gene (MD09G1009100) in Gala and three cultivars homozygous for the T allele of the SChr09_680633 SNP locus (late group). DNA ladder used was BioLabs Quick load® 1 kb Plus DNA ladder. Positions of primers are given in Figure S1 and primer sequences are in Table S3.

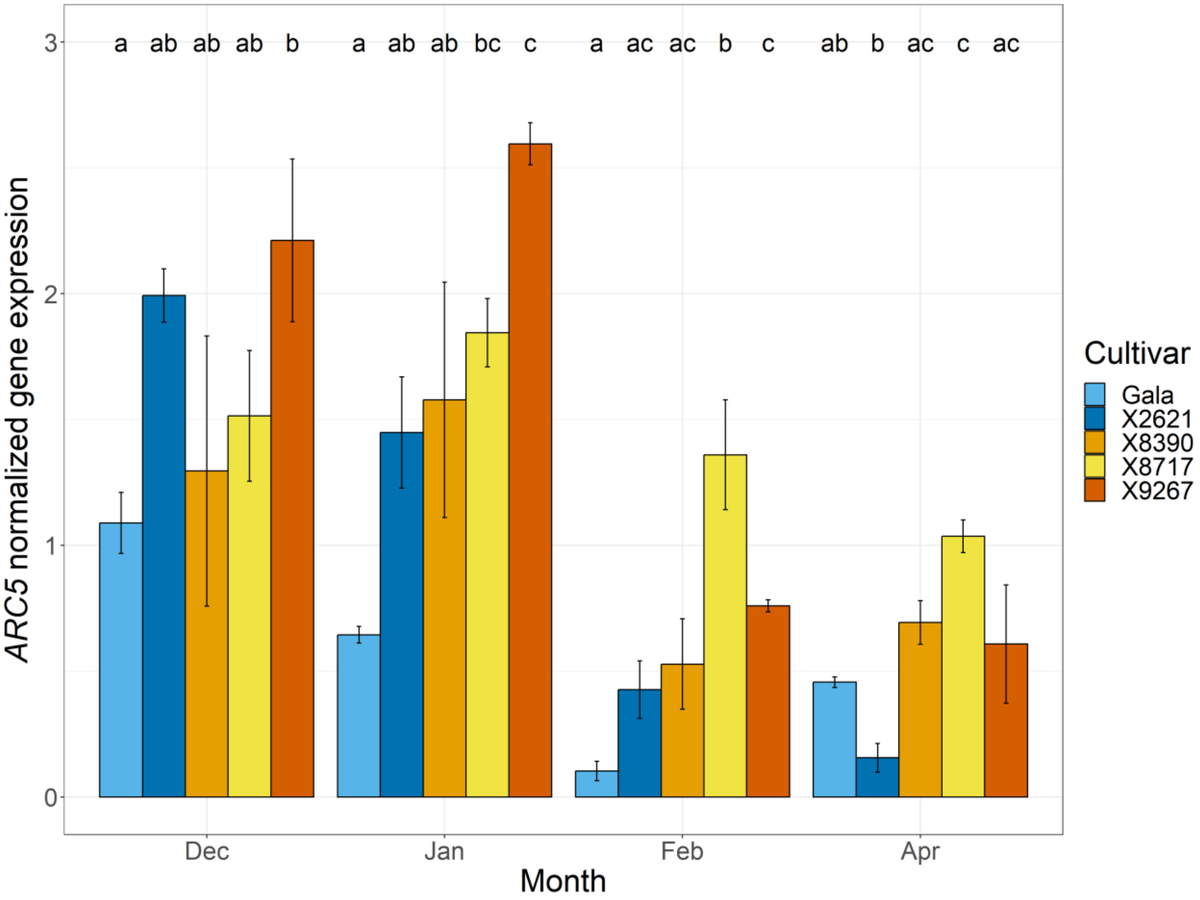

Figure S8. *ARC5*-normalized expression of *MdICE1* in bud tissue of two cultivars homozygous for the A allele of the SChr09_680633 SNP, Gala and X2621 (reference group, blue hues), and three cultivars homozygous for the T allele, X8390, X8717 and X9267 (late group, yellow-red hues). Sampling was carried out monthly from December 2022 to February 2023 and in April 2023. Bars represent mean of three biological replicates. Error bars represent the standard error of the mean. Different letters within a month indicate a significant difference (p-value ≤ 0.05) between cultivars.

Table S7. Results of the CUPSAT analysis showing the predicted effect of polymorphisms at the Phe156 position on the MdPRX10 protein.

Amino Acid Mutations

Amino acid	Overall Stability	Torsion*	Predicted ΔΔG (kcal/mol)
GLY	Destabilising	Unfavourable	-1.73
ALA	Destabilising	Favourable	-4.48
VAL	Destabilising	Favourable	-3.79
LEU	Destabilising	Unfavourable	-4.93
ILE	Destabilising	Unfavourable	-4.04
MET	Destabilising	Favourable	-1.55
PRO	Destabilising	Unfavourable	-5.29
TRP	Destabilising	Unfavourable	-4.35
SER	Destabilising	Favourable	-4.42
THR	Destabilising	Unfavourable	-1.68
GLN	Destabilising	Favourable	-4.67
LYS	Stabilising	Favourable	3.93
TYR	Destabilising	Favourable	-2.56
ASN	Destabilising	Unfavourable	-1.97
CYS	Destabilising	Favourable	-5.74
GLU	Destabilising	Unfavourable	-2.24
ASP	Destabilising	Unfavourable	-6.8
ARG	Destabilising	Favourable	-1.07
HIS	Stabilising	Favourable	2.44

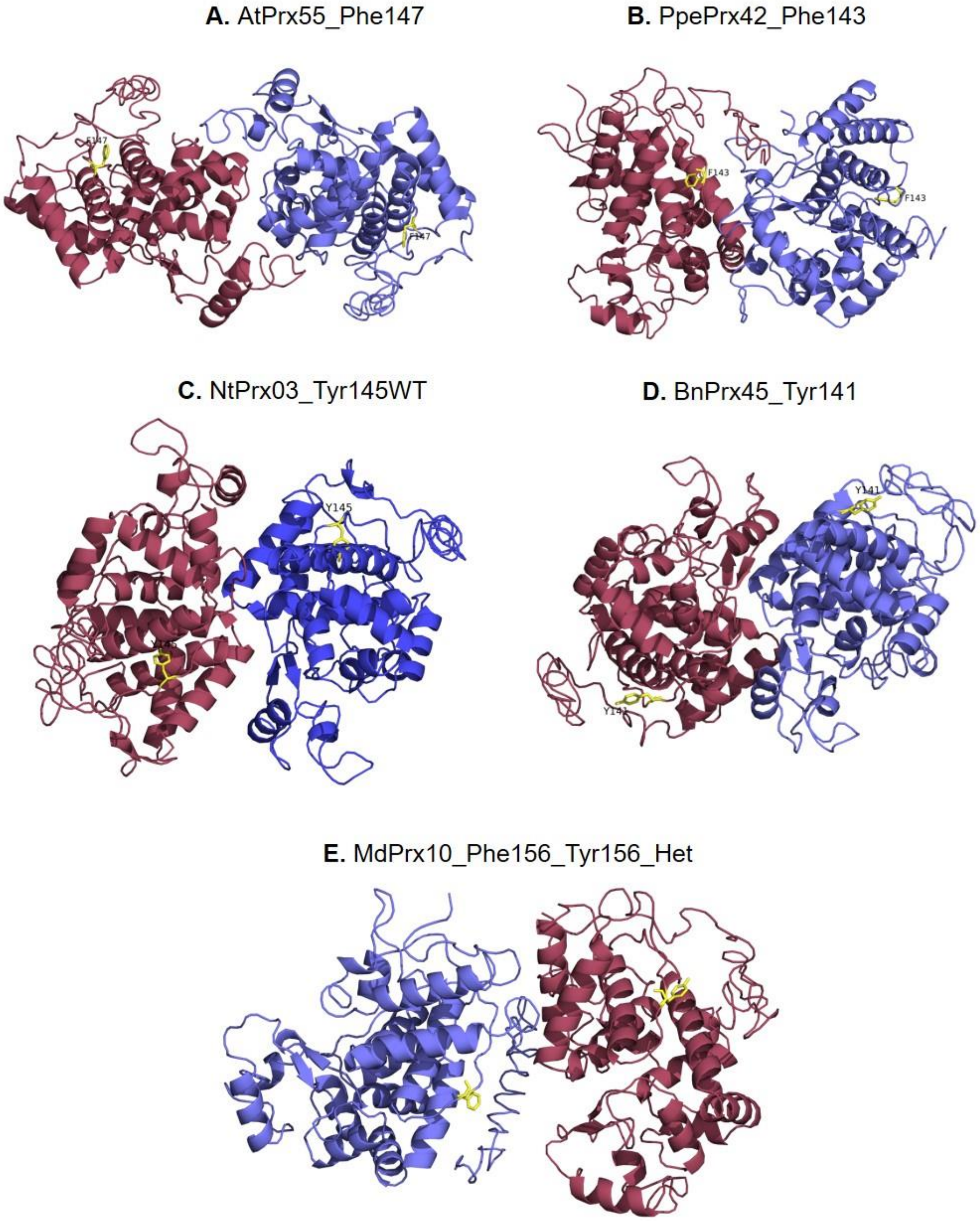

Figure S9. Simulations of protein-protein docking between Class III peroxidases from different species. **A**, AtPRX55 from *A. thaliana* (NP_001332223.1), **B**, PpePRX42 from *Prunus persica* (XP_007216155.2), **C**, NtPRX03 from *Nicotiana tabacum* (XP_009783800.1) and **D**, BnPRX45 from *Brassica napus* (XP_013596126.1). The proteins contain either Phe (**A**, **B**) or Tyr (**C**, **D**) at the corresponding 156 position in MdPRX10. The structure of the MdPRX10 Phe156/Tyr156 heterodimer is shown in (**E**).
